# LGR5+ Intestinal Stem Cells Display Sex-Dependent Radiosensitivity

**DOI:** 10.3390/cells13010046

**Published:** 2023-12-25

**Authors:** Ryan C. Zitter, Rishi Man Chugh, Payel Bhanja, Bruce F. Kimler, Subhrajit Saha

**Affiliations:** 1 Department of Radiation Oncology, University of Kansas Medical Center, Kansas City, KS 66160, USA; ryan.zitter@nm.org (R.C.Z.); rchugh@kumc.edu (R.M.C.); pbhanja@kumc.edu (P.B.); bkimler@kumc.edu (B.F.K.); 2 Department of Cancer Biology, University of Kansas Medical Center, Kansas City, KS 66160, USA

**Keywords:** LGR5+, radiosensitivity, oxidative stress, ISCs

## Abstract

Tissue radiosensitivity plays a critical role in the overall outcome of radiation therapy. Identifying characteristics that predict how a patient may respond to radiotherapy enables clinicians to maximize the therapeutic window. Limited clinical data have suggested a difference in male and female radiotherapy outcomes. Radiotherapy for gastrointestinal malignancy is still a challenge due to intestinal sensitivity to radiation toxicity. In this manuscript, we demonstrated sex-specific differences in intestinal epithelial radiosensitivity. In a mouse model of abdominal irradiation, we observed a significant increase in oxidative stress and injury in males compared to females. Lgr5+ve intestinal stem cells from male mice showed higher sensitivity to radiation-induced toxicity. However, sex-specific differences in intestinal radiosensitivity were not dependent on sex hormones, as we demonstrated similar sex-specific radiosensitivity differences in pre-pubescent mice. In an ex vivo study, we found that patient-derived intestinal organoid (PID) from males showed higher sensitivity to radiation compared to females as evident from loss of budding crypts, organoid size, and membrane integrity. Transcriptomic analysis of human Lgr5+ intestinal stem cells suggested radiation-induced upregulation of mitochondrial oxidative metabolism in males compared to females, a possible mechanism for radiosensitivity differences.

## 1. Introduction

Radiotherapy continues to play a role in nearly half of all cancer patients’ treatments [1]. While incredible technological advancements have taken place in radiotherapy, nearly all have been focused on anatomical precision. While this has done much in limiting radiation exposure to normal tissue, empiric dosing and fractionation is a cornerstone of the specialty. Personalization of radiotherapy remains, for the most part, in the hands of clinical judgment. Tailoring dose to individual patients is critically important. Pauses in radiotherapy caused by radiotoxicity can be associated with a decrease in local control by as much as 16% [2]. Identifying patients who are inherently radiosensitive and more prone to these toxicities could allow them to receive extended fractionation to minimize toxicity. Conversely, radioresistant individuals may receive a higher dose, giving them a better chance of local control.

The simplest personalization a patient could receive in their therapy design is on the basis of their sex. 

Sex-based differences in radiotoxicity have been found clinically in hepatic [3] and thyroid [4] tissues. Preclinical models have also demonstrated differences in normal tissue radiotoxicity [5,6]. Identifying radiosensitive patients is most important when considering acute radiation effect on the intestines. The intestine is the most radiosensitive among abdominal tissues undergoing abdominal radiation exposure [7]. Nearly all patients receiving abdominal radiotherapy will experience some form of gastrointestinal symptoms [8,9]. This is due to the high radiosensitivity of the intestinal epithelium [10] consisting of intestinal stem cells (ISCs) with a high self-renewal rate. The intestinal microvasculature has also been proposed as a reason for this heightened sensitivity [11]. However, recent reports have largely refuted [12] the importance of intestinal microvasculature, leaving ISCs as a primary suspect for determining intestinal radiosensitivity [13].

The intestinal epithelium is a single cell layer thick and organized into repetitive crypt-villous units, with stem cells residing at the base of the crypt and renewing cells moving upward in the crypt in a conveyer belt fashion [14]. There are two different types of stem cells at the crypt bottom. Bmi1-positive ISCs are long-lived, label-retaining stem cells present at the +4 position of the crypt base and known as reserve stem cells (RSC). These Bmi1+ve ISCs interconvert with more rapidly proliferating LRG5+ve stem cells known as CBCs [15,16]. Loss of the LGR5+ stem cells in the radiation response is catastrophic to the architecture of the epithelium, highlighting their essential role in radiation toxicity [17]. 

Considering the radiosensitivity of LGR5+ ISCs compared to RSCs, we rationalize that sex-dependent differences in intestinal radiosensitivity depends on the LGR5+ population. The pro-growth hormone estrogen is often the cited difference for any apparent radioresistance observed in the clinic [18,19,20] and has been described in preclinical studies as well [21]. However, recent evidence clearly suggests that sex hormones [22] have no influence in ISC homeostasis and repair. In this study, we attempt to determine if there are significant differences in the intestinal radiation response between males and females. We have demonstrated sex-specific differences in LGR5+ ISC radiosensitivity having a distinct transcriptomic profile.

## 2. Materials and Methods

### 2.1. Animals

Eight- to twelve-week-old male and female C57BL6/J mice, Lgr5-eGFP-IRES-CreERT2 mice, Gt (ROSA)26Sortm4(ACTB-tdTomato- EGFP) Luo/J mice, and B6. Cg-Gt (ROSA)26Sortm9(CAG- tdTomato) Hze/J mice were purchased from Jackson laboratories. Lgr5-eGFP-IRES-CreERT2 mice were crossed with Gt (ROSA)26Sortm4(ACTB-tdTomato-EGFP) Luo/J mice (Jackson Laboratories) to generate Lgr5/eGFP-IRES-Cre-ERT2; R26-ACTB-tdTomato-EGFP [23,24]. In Gt (ROSA)26Sortm4(ACTB- tdTomato-EGFP) Luo/J mice tdTomato is constitutively expressed (independent of Cre recombination) in the membrane of all the cells, and therefore allows better visualization of cellular morphology. Lgr5-eGFP-IRES-CreERT2 mice were crossed with B6 Cg-Gt (ROSA)26Sortm9(CAG-tdTomato) Hze/J mice (Jackson Laboratories) to generate the Lgr5-eGFP-IRES-CreERT2; the Rosa26-CAG-tdTomato heterozygote was used for lineage tracing experiments. All the mice were maintained ad libitum, and all the studies were performed under the guidelines and protocols of the Institutional Animal Care and Use Committee of the University of Kansas Medical Center. All the animal experimental protocols were approved by the Institutional Animal Care and Use Committee of the University of Kansas Medical Center (ACUP number 2019-2487).

### 2.2. In Vitro Culture of Intestinal Crypt Organoids

Small intestines from male and female C57BL6/J, Lgr5-eGFP-IRES-CreERT2; R26-ACTB-tdTomato-EGFP mice and non-malignant colon tissue from human surgical specimens were used for crypt isolation and development of the ex vivo organoid culture [25,26]. Human colon tissue samples were obtained from the University of Kansas Medical Center Biospecimen Repository under an IRB-approved protocol (HSC#5929). Tissue samples were thoroughly cleaned with cold PBS followed by scraping and chopping of tissue into 5 mm pieces, followed by thorough washing with cold PBS, and then incubation at room temperature (mouse tissue) or on ice (human tissue) in a gentle dissociation buffer (Stemcell Technologies, Vancouver, BC, Canada, Cat No. 100-0485) for 20–30 min with gentle shaking. For mouse tissue, the dissociation buffer was subsequently replaced with cold 0.1% BSA in PBS 4 times and vigorously pipetted followed by passing through a 70 μm filter to obtain 1–4 fractions of the supernatants enriched in crypt stem cells. Human tissue required an additional step of pipetting in ice-cold DMEM with 1% BSA to liberate crypts from the tissue. The mouse and human tissue isolated crypts were centrifuged, resuspended in a small volume of PBS containing 0.1% BSA, and were embedded in Matrigel (Corning, Glendale, CA, USA, Cat No. 356231) and cultured in respective intestinal organoid Medium (Stemcell Technologies) with their respective supplements and gentamycin (50 mg/mL). The quantification of organoids per well was performed using an EVOS Microscope (Thermo Fisher Scientific, Waltham, MA, USA). Images of organoids were acquired using fluorescent (Nikon 80i, Melville, NY, USA) and confocal (Nikon, A1RMP) microscopy. 

### 2.3. Irradiation Procedure

Abdominal irradiation (AIR) was performed on mice anesthetized with 87.5 mg/kg of ketamine and 12.5 mg/kg of xylazine using a small animal radiation research platform (XenX; Xstrahl, Suwanee, GA, USA) (0.67 mm Cu HVL) at a dose rate of 2.26 Gy/min at 220 kVp and 13 mA. A 3 cm^2^ area of the mice containing the gastrointestinal tract (GI) was irradiated, thus shielding the upper thorax, head, and neck, as well as the lower and upper extremities, and protecting a significant portion of the bone marrow. This radiation exposure model predominantly induces radiation-induced gastrointestinal injury. For partial body irradiation (PBI), the mouse was irradiated at a dose rate of 2.82 Gy/min at 220 kVp and 13 mA by shielding one leg from radiation exposure. In order to achieve a uniform distribution of the radiation dose, a deliberate approach was taken in the radiation delivery process. Specifically, half of the total irradiation dose was administered from the anterior–posterior (AP) direction, and the remaining half was delivered from the posterior–anterior (PA) direction. This strategy helps to mitigate the potential for dose inhomogeneity within the target tissue. The total irradiation time required to administer the intended radiation dose was calculated with respect to dose rate, radiation field size, and fractional depth dose to ensure accurate radiation dosimetry.

### 2.4. Histology

Animals were euthanized when moribund or at 3.5 days after irradiation for time-course experiments, and intestines were collected for histological analysis [27]. The intestinal tissues of experimental animals underwent a thorough wash in PBS to eliminate intestinal contents. Subsequently, the jejunum was fixed using 10% neutral-buffered formalin followed by embedding in paraffin and sectioning into 5 μm-thick sections for hematoxylin and eosin (H&E) and immunohistochemistry (IHC) staining. All the H&E staining was performed at the Pathology Core Facility at the University of Kansas Cancer Center (Kansas City, KS, USA). Histopathological analysis was conducted for the assessment of crypt proliferation rate, villous length, and crypt depth to analyze the structural and cellular alterations within the intestinal tissue morphology.

### 2.5. Crypt Proliferation Rate

To investigate villous cell proliferation, the jejunum portion of the intestinal tissue was processed for paraffin embedding and Ki67 immunohistochemistry. The tissue sections were processed for deparaffinization, rehydration through a series of graded alcohol solutions, and an overnight incubation at room temperature with a monoclonal anti-Ki67 antibody (M7240 MIB-1; Dako, Santa Clara, CA, USA). Nuclear staining was performed using a chromogenic substrate, streptavidin-peroxidase and diaminobenzidine (DAB). In addition, a light counterstaining with hematoxylin was performed for optimal visualization. The identification of murine crypts within the tissue sections was employed using an established histological criterion as previously reported by Potten et al. [28]. To quantify the proliferation rate of the cells, the percentage of Ki67-positive cells was counted over the total cell count within each crypt. A total of 50 crypts was counted for each animal.

### 2.6. Determination of Villous Length and Crypt Depth

The villous length and crypt depth were independently and objectively analyzed and quantitated in a double-blinded manner from coded digital photographs from H&E-stained slides using ImageJ 1.37 software. The crypt depth was measured in pixels from the bottom of the crypt to the crypt villus junction. Villous length was determined by measuring the length from the crypt villus junction to the villous tip. The measurement in pixels was converted to length or depth (in μm) by applying the conversion factor 1.46 pixels/μm.

### 2.7. qPCR Analysis

qPCR was performed to determine the expression of oxidative stress responsive genes, mitochondrial biogenesis genes, and stem cells marker and Wnt target genes in intestinal epithelial cells. Total RNA was isolated from the samples using the RNeasy micro/mini kit from (Qiagen, Germantown, MD, USA). The concentration and purity of the extracted RNA were checked using a NanoDrop spectrometer (Thermo Scientific, Waltham, MA, USA). Then, 1 μg of total RNA was reverse transcribed using RNA to cDNA EcoDry™ Premix (Double Primed) (Takara Bio USA Inc., San Jose, CA, USA) according to the manufacturer’s instructions. qPCR was performed using the QuantStudio™ 7 Flex Real-Time PCR System (Applied Biosystems™, New York, NY, USA) and SYBR Green Supermix (Bio-Rad, Hercules, CA, USA) with specific primers to the target genes in a 20 μL final reaction volume. GAPDH was used as a reference gene for sample normalization. The primer sequences are listed in Supplementary Table S1. The delta-delta threshold cycle (ΔΔCt) method was used to calculate the fold change expression level in the samples. All the gene expression experiments were repeated two times with a minimum number of three biological replicates.

### 2.8. Western Blot Analysis

Total OXPHOS (oxidative phosphorylation) expression was analyzed in irradiated isolated intestinal epithelial cells. Isolated cells were lysed with 1× RIPA buffer (Cell Signaling, Boston, MA, USA) containing a protease and phosphatase inhibitor cocktail (Thermo Fisher Scientific Inc., Carlsbad, CA, USA) to isolate the total protein. The total protein concentration of the samples was determined by the BCA method [29]. For analysis, 30 µg of protein samples with 1× gel loading buffer were separated by SDS-PAGE and transferred to PVDF membranes. After protein transfer, the membrane was blocked with 5% skim milk for one hour at room temperature, followed by overnight incubation at 40C with total OXPHOS antibody cocktail (ab110413, Abcam, Boston, MA, USA). After incubation with primary and secondary antibodies, the membrane was developed with Trident Femto Western HRP substrate (GeneTex, Irvine, CA, USA) and imaged using a ChemiDoc XRS + molecular imager (Bio-Rad, Hercules, CA, USA). β-Actin was used as an internal control for normalization. The western blot experiment was repeated two times with three biological replicates in each experiment.

### 2.9. In Vivo Lineage Tracing Assay

To investigate the role of Lgr5-expressing intestinal stem cells (ISCs) in tissue regeneration under homeostatic conditions and in response to radiation injury, an in vivo lineage tracing assay was performed. Lgr5-eGFP-IRES-CreERT2 mice were crossed with B6. Cg-Gt (ROSA)26Sortm9(CAG-tdTomato) Hze/J mice (Jackson Laboratories) to generate the Lgr5-eGFP- IRES-CreERT2; Rosa26-CAG-tdTomato heterozygote mice. To initiate lineage tracing, adult Cre reporter mice were injected with tamoxifen (Sigma-Aldrich, St. Louis, MO, USA; 9 mg per 40 g body weight, intraperitoneally), allowing for the labeling of Lgr5+ lineages and their subsequent tdTomato (tdT)-positive progeny. This approach enabled the identification and tracking of Lgr5 ISC lineages. For radiation injury studies, mice received 12.5 Gy of AIR, and tissue was harvested on day 10 post irradiation.

### 2.10. LGR5+ Cell Isolation

Human intestinal tissues from males and females (adult non-Hispanic whites) were received from the University of Kansas Medical Center Biorepository core facility (BRCF). All the tissues were collected in the biorepository core facility in strict accordance with the guidelines and approval of the University of Kansas Medical Center Institutional Review Board (IRB number HUS35929). The patients’ confidentiality and anonymity were maintained throughout the study, and all the patient data were de-identified to protect individual privacy. Tissues were dissociated into a single-cell suspension using a gentleMACS Dissociator (Miltenyi Biotec, Inc., San Jose, CA, USA) and maintained at 4 °C throughout preparation. Cell numbers were determined using an automated Cell Counter T20 (Bio-Rad, Hercules, CA, USA). Lgr5-positive cells were isolated using an Anti-LGR5 MicroBeads kit (Miltenyi Biotec, Inc., USA) according to manufacturer’s protocol. In brief, organoid derived cells were incubated with Anti-LGR5 MicroBeads for 20 min in the dark at 4 °C. Finally, cells were passed through a MACS magnetic column in order to capture cells labeled with MicroBeads. 

### 2.11. RNAseq Analysis

Isolated human LGR5+ cells were stored at −80 °C until processed for RNA isolation using a RNeasy micro kit as per manufacturer instruction’s (Qiagen, Germantown, MD, USA). RNA processing, cDNA generation, mRNA-Seq library prep, and library quality control were performed at the UNC Advanced Analytics Core. RNA was cleaned and concentrated using the Zymo RNA Clean & Concentrator kit (Zymo, Irvine, CA, USA Cat # R1013). Full-length cDNA was generated using the TaKaRa SMART-Seq v4 Ultra Low kit (TaKaRa Bio USA Inc., San Jose, CA, USA, Cat # 634889). mRNA-Seq libraries were then prepared using the Illumina Nextera XT DNA Library Preparation kit (Illumina, San Diego, CA, USA Cat # FC-131-1024). Then, 1 ng of cDNA was used as input, and 12 amplification cycles were used during PCR enrichment. Prepared libraries were quantified using the Qubit dsDNA Quantitation High-Sensitivity kit (Thermofisher, Carlsbad, CA, USA Cat # Q32851), and fragment size was assessed using the Agilent Bioanalyzer High-Sensitivity DNA kit (Agilent, Santa Clara, CA, USA Cat # 5067-4626). Libraries were then pooled in an equimolar fashion. The library pool was sequenced at the UNC CRISPR Screening Facility using the Illumina NextSeq 500 instrument. A High-Output 75-cycle flow cell (Illumina, San Diego, CA, USA Cat # 20024906) was used for single-end sequencing with a read length of 76 bp, PhiX concentration of 1%, and loading concentration of 1.65 pM. This sequencing process ensures the acquisition of high-quality data for downstream analysis. The resulting gene expression levels were transformed using a variance stabilizing transformation (VST) in order to compare normalized levels across all the tested samples. Heat maps were created using the online software Morpheus from the Broad Institute. mRNA expression underwent gene enrichment using Gene Ontology Enrichment Analysis with the PANTHER classification system [30].

### 2.12. Serum Exosomal miRNA Analysis

Exosomal miRNA analysis was performed on the serum samples from five of the same male and female patients mentioned before. Serum exosomal miRNA analysis was performed by System Biosciences (System Biosciences LLC, Palo Alto, CA, USA) at their laboratory. In brief, exosome was isolated from serum following the SBI’s ExoQuick Exosome Precipitation method. Exosomal RNA was then extracted from these serum exosomes. Next-Generation Sequencing libraries were prepared and sequenced on an Illumina NextSeq instrument with 1 × 75 bp single-end reads at an approximate depth of 10–15 million reads per sample. RNA or library QC was checked for further analysis. Raw data were analyzed using UCSC’s analysis pipeline (formerly Maverix Biomics, San Mateo, CA, USA). Mouse intestinal sampling regions were chosen at random for digital acquisition for quantitation. Digital image data were evaluated in a blinded manner as to treatment. A two-tailed non-parametric Mann–Whitney test was used to compare differences between experimental groups, with *p* < 0.05 being considered statistically significant. For descriptive purposes, all data are presented as mean ± standard deviation (SD). 

## 3. Results

### 3.1. Female Intestinal Epithelium Is Resistant to Radiation-Induced Toxicity

In this study, our aim was to investigate potential sex-dependent differences in intestinal epithelial cell radiosensitivity. Additionally, we aimed to ascertain whether such differences, if present, could be attributed to differences in the LGR5+ ISCs between males and females. These cells play a pivotal role in maintaining the regenerative capacity of the intestinal lining, where constant cell turnover is essential for tissue homeostasis.

**Figure 1:**
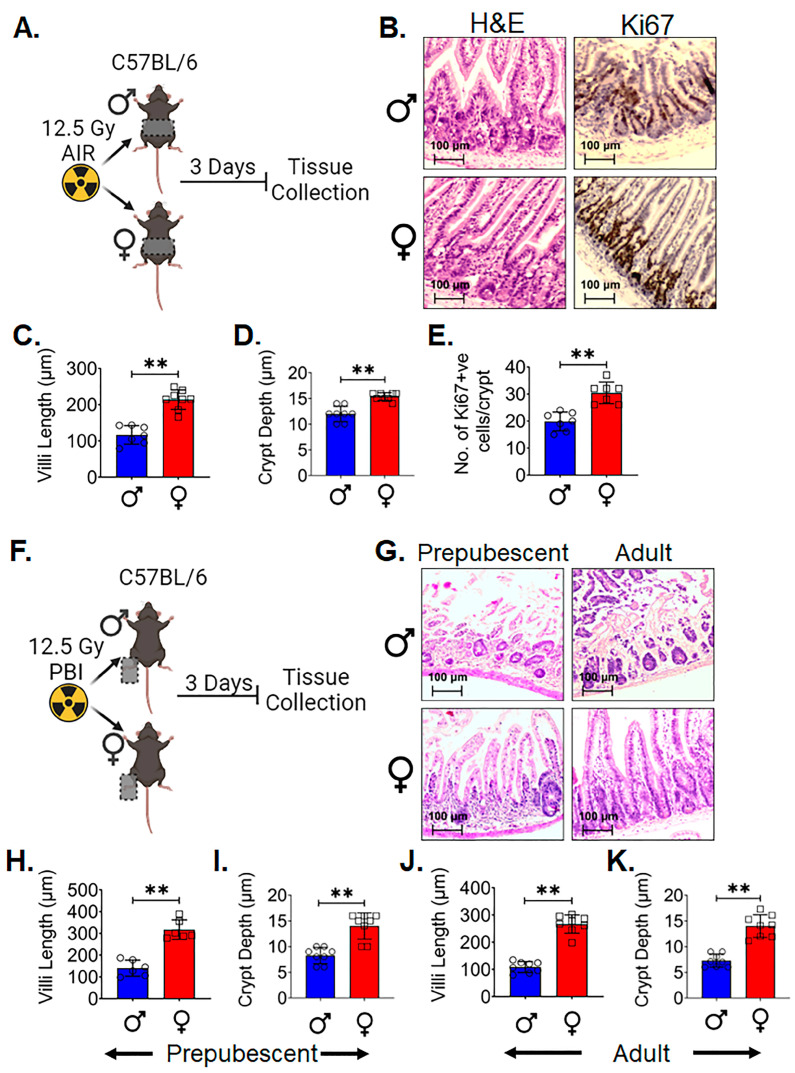
Female intestinal epithelium is resistant to radiation-induced toxicity: (**A**) Schematic representation of the experimental design, (**B**) histological analysis of intestinal tissue. Adult female C57BL/6 mice exhibited significantly increased (**C**) villi length and (**D**) crypt depth compared to males, along with a (**E**) higher number of Ki-67-positive proliferating cells per crypt. (**F**). Schematic representation of the experimental design using prepubescent (4-week-old) and postpubescent (12-week-old) male and female mice to investigate the role of systemic hormonal differences. (**G**–**K**) Histological analysis of intestinal tissue of both age groups showed that female had significantly increased villi length and crypt depth in comparison to males. (*n* = 5 mice per group). Data presented as the mean ± SD. (Significance level, **: *p* < 0.005).

Histopathological analysis of the intestinal tissue [31] acquired three days post 12.5 Gy AIR (Figure 1A) demonstrated significantly higher villi length (*p* < 0.005) (Figure 1B,C) and crypt depth (*p* < 0.005) (Figure 1B,D) in adult female C57BL/6 mice compared to male. A higher number of Ki-67-positive proliferating cells per crypt was observed in female intestinal epithelium (*p* < 0.005) (Figure 1B,E) compared to male. In order to determine if the above finding was dependent on systemic hormonal differences such as estrogen, a separate experiment was performed in both pre- (4-week-old) and postpubescent (12-week-old) mice. Male and female C57BL/6 mice, 4 weeks or 12 weeks old, were exposed to a lethal dose (12.5 Gy) partial body irradiation (PBI), keeping one hind leg out of radiation field for partial bone-marrow sparing. At 3 days post irradiation, the jejunum was collected for histological analysis (Figure 1F). H&E staining of tissues from both age groups showed that female mice had a significant increase in villi length (*p* < 0.005 and *p* < 0.005, respectively) and crypt depth (*p* < 0.005 and *p* < 0.005, respectively) in comparison to the male mice (Figure 1G–K). These data indicate that differences observed in radiosensitivity are independent of major hormonal differences between male and female mice.

### 3.2. LGR5+ ISCs in Females Are More Radioresistant Compared to Males

**Figure 2:**
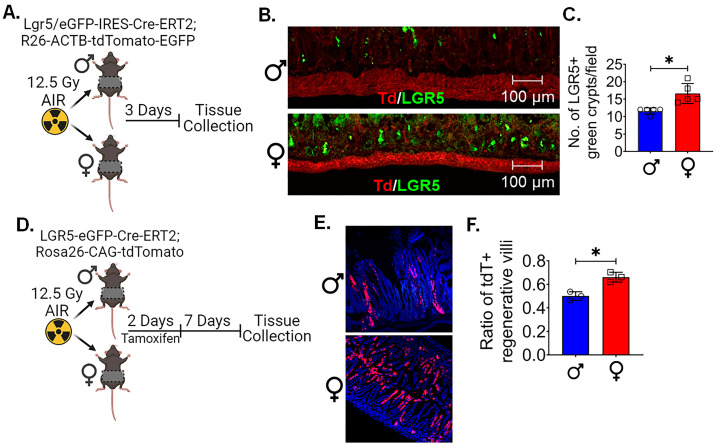
Female LGR5+ cells are radioresistant and possess regenerative potential: (**A**) Schematic representation of experimental design to analyze the survival of LGR5+ cells using Lgr5/eGFP-IRES-Cre-ERT2; R26-ACTB-tdTomato-EGFP mice following irradiation. (**B**,**C**) A histological analysis of tissue acquired three days post irradiation showed a significant difference in the number of crypts containing LGR5+ cells in females compared to males. (**D**) Schematic representation of experimental design to investigate the regenerative capacity of surviving ISCs; a lineage tracing assay was performed using Lgr5-EGFP-ires-CreERT2-R26-CAG-tdT mice following irradiation. (**E**,**F**) Histological analysis showed, following irradiation and tamoxifen treatment, that female intestinal epithelium exhibited a significantly higher number of tdT-positive cells in regenerative villi compared to male mice. (*n* = 5 mice per group). Data presented as the mean ± SD. (Significance level, *: *p* < 0.05).

To examine the radiosensitivity of ISCs, we determined the survival of LGR5+ crypt cells in Lgr5/eGFP-IRES-Cre-ERT2; R26-ACTB-tdTomato-EGFP mice. Age-matched male and female mice were irradiated with 12.5 Gy AIR (Figure 2A). As the regeneration of the intestinal epithelium follows the apoptotic phase at 2–3 days [32], assessing the LGR5+ population 3 days post irradiation would provide an accurate depiction of the surviving fraction. Histological analysis showed a significant difference in the number of crypts containing LGR5+ cells in female mice compared to the male mice (*p* < 0.05) (Figure 2B,C). This suggests that LGR5+ cells in female mice were more radioresistant than in male mice.

### 3.3. Female LGR5+ Cells Are More Capable of Proliferation Following Radiation

After determining that female LGR5+ cells are more resistant to radiation, we wanted to assess if the surviving ISCs were capable of regeneration by performing in vivo lineage tracing assay using Lgr5-EGFP-ires-CreERT2-R26-CAG-tdT mice. In this mouse, tamoxifen-mediated activation of cre-recombinase under the Lgr5 promoter expresses tdTomato in epithelial cells derived from Lgr5-positive ISCs. Therefore, quantification of these tdTomato (tdT)-positive cells in irradiated epithelium in male and female mice determines the regenerative response of Lgr5-positive ISCs. Mice were exposed to 12.5 Gy AIR, followed by tamoxifen treatment. The presence of tdT-positive cells was demonstrated in the crypt epithelium of male and female intestinal tissue (Figure 2D). However, female intestinal epithelium showed a significantly higher number of tdT-positive cells in regenerative villi compared to the male mice (*p* < 0.05) (Figure 2E,F). All this evidence clearly demonstrates the higher regenerative response of female mice LGR5+ ISCs following irradiation compared to males.

### 3.4. Female Intestinal Epithelium Showed Less Oxidative Stress, Mitochondrial Biogenesis and Better Stemness

**Figure 3:**
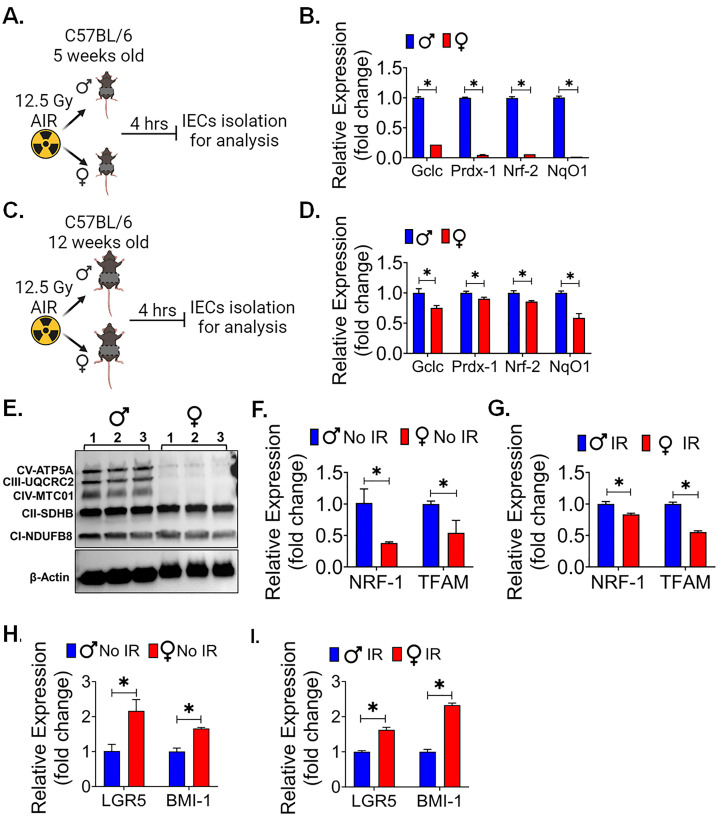
Female intestinal epithelium shows less oxidative stress and mitochondrial biogenesis and better stemness: Schematic representation of experimental design using (**A**) 5-week-old and (**C**) 12-week-old male and female mice for analyzing the expression of oxidative stress genes following radiation exposure. (**B**,**D**) Comparative analysis of oxidative responsive genes expression (Gclc, Prdx-1, Nrf-2, and NqO1) analysis in 5- and 12-week-old mice showed that females had less oxidative stress responsive gene compared to male mice after radiation exposure. (**E**) Irradiated female IECs showed less OXPHOS proteins expression, which plays a crucial role in mitochondrial function. (**F**,**G**) qPCR analysis of mitochondrial biogenesis genes (NRF-1 and TFAM) in non-irradiated and irradiated IECs showed that females had less mitochondrial biogenesis genes expression compared to males. (**H**,**I**) qPCR analysis of major stem cell markers, LGR5 and BMI expression, in non-irradiated and irradiated IECs demonstrated female IECs had a significantly higher expression of both the LGR5 and BMI-1 marker compared to male mice. (*n* = 3 mice per group). Data presented as the mean ± SD. (Significance level, *: *p* < 0.05).

To investigate the sex-specific differences in radiation-induced oxidative stress in intestinal radiosensitivity, 5- and 12-week-old male and female mice were exposed to 12.5 Gy AIR and IECs were isolated 4 hrs after radiation exposure to analyze the reactive oxygen species (ROS) responsive gene (Figure 3A,C). The qPCR analysis of 5-week-old male mice showed a significantly higher expression of oxidative stress genes for *Gclc*, *Prdx-1*, *Nrf-2*, and *NqO1* (*p* < 0.05 for all) in comparison to the female mice (Figure 3B). Interestingly, 12-week-old male mice were also showing the similar pattern of oxidative stress genes expression for *Gclc*, *Prdx-1*, *Nrf-2*, and *NqO1* (*p* < 0.05 for all) in comparison to the female mice (Figure 3D). As a significant amount of radiation-induced DNA damage is transmitted by oxidative stress [33], these findings suggested an explanation for the observed differences in radiation sensitivity. Interestingly, the western blot analysis of IECs also showed a lower expression of some of the OXPHOS proteins (Figure 3E, Supplementary Figure S1) in female mice compared to the male mice IECs. It is well documented that radiation upregulates the expression of genes related to OXPHOS [34] and mitochondrial biogenesis [35]. Our quantitative PCR (qPCR) analysis of IECs from both male and female mice confirmed these findings, indicating that the expression of mitochondrial biogenesis genes, specifically *NRF-1* and *TFAM*, was notably significantly lower in non-irradiated (*p* < 0.05 for each) (Figure 3F) and irradiated (*p* < 0.05 for each) (Figure 3G) female mouse IECs. These results collectively suggest a reduced capacity for mitochondrial biogenesis in female mice.

Prior studies have demonstrated the role of decreased OXPHOS and ROS production in enhancing the stemness in IECs [36,37]. Importantly, ISCs often rely on glycolysis as their primary energy source, which may contribute to the maintenance of their stem cell characteristics [38]. The observed reduction in mitochondrial biogenesis in female IECs may potentially redirect cellular metabolism toward a more glycolytic profile, thereby preserving their stemness [39]. Therefore, we also examined the major stem cells marker *LGR5* and *BMI* expression in IECs. qPCR analysis shows that female IECs had significantly higher expression of *LGR5* and *BMI* in non-irradiated (Figure 3H) and irradiated (Figure 3I) IECs cells compared to the male IECs (*p* < 0.05 for all). All these observations suggest that reduced levels of these oxidative events and mitochondrial biogenesis might support the stemness of ISCs in females and, therefore, demonstrate resistance against radiation compared to males.

### 3.5. Female Intestinal Epithelium Shows Less Radiationinduced DNA Damage

**Figure 4:**
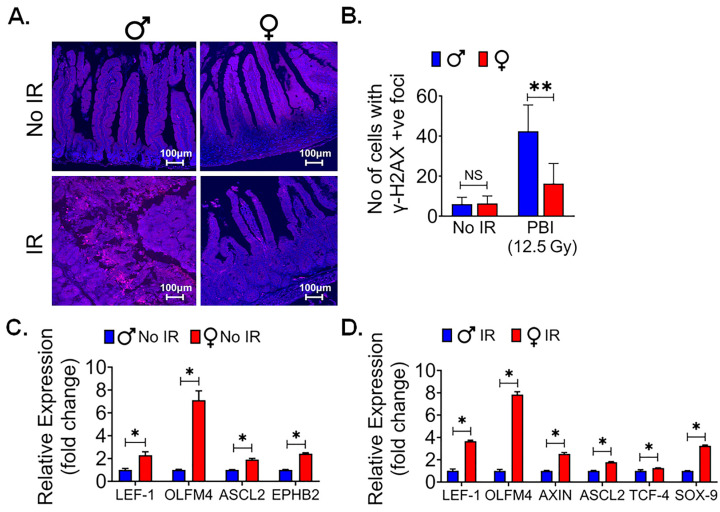
Female intestinal epithelium is resistant to radiation-induced DNA damage: (**A**) Immunofluorescent staining for γ-H2AX in non-irradiated and irradiated male and female mice intestinal tissue. (**B**) No significant differences were observed in γ-H2AX foci between non-irradiated male and female mice, while irradiated male mice intestinal tissue showed a significantly higher number of γ-H2AX foci compared to their female counterparts. (**C**,**D**) The qPCR analysis of β-catenin target genes in non-irradiated and irradiated IECs from both female and male mice revealed a significant upregulation of multiple β-catenin target genes in female IECs compared to male IECs in both non-irradiated and irradiated mice IECs. (*n* = 3 mice per group). Data presented as the mean ± SD. (Significance level, *: *p* < 0.05, **: *p* < 0.005, NS: not significant).

Radiation exposure is known to induce DNA damage in various tissues, including the sensitive intestinal epithelium. Gamma-H2AX (γ-H2AX) is a phosphorylated form of the histone protein H2AX and is known to form distinct foci at sites of DNA double-strand breaks (DSBs), which are a prevalent and critical form of DNA damage triggered by ionizing radiation [40]. To assess and compare the response to radiation-induced DNA damage between male and female mice, particularly following exposure to a significant dose of 12.5 Gy of PBI, immunofluorescent staining for γ-H2AX was performed in the intestinal tissue sections to visualize and quantify the extent of DNA damage in the intestinal epithelium of both male and female mice. This approach allows for a precise assessment of potential sex-based differences in the response to radiation-induced DNA damage within the intestinal tissue. The immunofluorescent staining showed no significant differences for γ-H2AX staining in intestinal tissue between non-irradiated male and female mice (Figure 4A). However, a significantly higher number of γ-H2AX foci was observed in irradiated male mice in comparison to their female counterparts (*p* < 0.005, Figure 4A,B). This finding indicates that, following irradiation, male mice exhibited a greater extent of radiation-induced DNA damage, specifically in the form of γ-H2AX foci formation. These difference in DNA damage may contribute to the observed differences in radiosensitivity between male and female mice.

Canonical WNT signaling is a key for intestinal epithelial repair and regeneration. We reported earlier that pharmacological activation of WNT β-catenin signaling can promote intestinal epithelial repair and regeneration and mitigate radiation-induced intestinal injury [25,41]. To understand the role of WNT β-catenin signaling, we conducted qPCR analysis of WNT target genes in IECs from female and male mice. We found a significant upregulation of multiple β-catenin target genes in female IECs when compared to male IECs, both in non-irradiated (Figure 4C) and irradiated (Figure 4D) conditions (*p* < 0.05 for all). These findings suggest that the activation of WNT β-catenin signaling is more distinct in female IECs, highlighting a potential sex-specific difference in the response to WNT pathway activation in the context of intestinal epithelial radiosensitivity.

### 3.6. Female Ex Vivo Intestinal Organoid Is More RadioResistant

**Figure 5:**
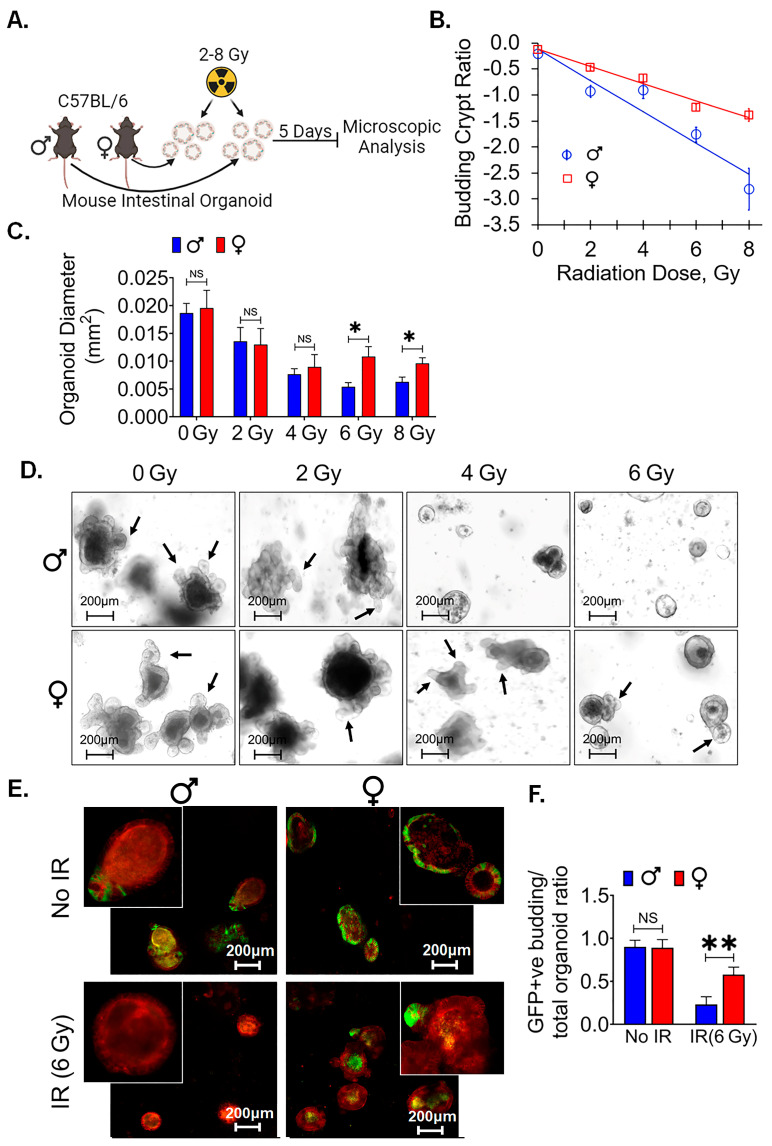
Female ex vivo intestinal organoid culture is more radioresistant: (**A**) Schematic representation of male and female mice intestinal organoids culture and radiation exposure for examining their morphological features. (**B**) A higher budding crypt ratio in female intestinal organoid compared to male following exposure to 0 −8 Gy of irradiation. (**C**,**D**) The assessment of the average cross-sectional area of female organoids, with noticeable increases, especially at higher radiation doses (6 and 8 Gy), when compared to male organoids. No significant differences were detected at radiation doses below 6 Gy. (**E**) Organoids derived from Lgr5/GFP-IRES-Cre-ERT2; R26-ACTB-tdTomato-EGFP knock-in mice. (**F**) No significant differences were observed in the ratio of GFP+ budding organoids to total organoids in non-irradiated male and female organoids. However, within 48 h post irradiation, female mice organoids exhibited a significantly higher GFP+ budding organoid/total organoid ratio compared to male mice organoids. Data presented as the mean ± SD. (Significance level, *: *p* < 0.05, **: *p* < 0.005, NS: not significant).

Organoids have become an established ex vivo model to study intestinal stem cells’ [42] survival and function. To identify the local differences in LGR5+ cells between male and female mice, ex vivo intestinal organoids were cultured, subjected to various radiation doses, and examined for their morphological features (Figure 5A). Specifically, we focused on assessing the organoids’ ability to regenerate, as indicated by the budding phenomenon. Our observations revealed a significant difference in the budding crypt ratio between male and female intestinal organoids when exposed to radiation doses ranging from 0 to 8 Gy (Figure 5B). The exponential decrease in budding crypts with the increasing dose was 80% steeper in organoids derived from males than those derived from females (slopes of 0.30 Gy-1 versus 0.165 Gy-1, *p* < 0.001). Furthermore, we observed a significantly higher average cross-sectional area of female organoids, at radiation doses of 6 and 8 Gy, in comparison to their male counterparts (*p* < 0.05 for each). However, no significant differences were detected at radiation doses below 6 Gy (Figure 5C,D). These data show the morphological disparities within organoids derived from male and female mice exposed to varying radiation doses yielded an intriguing finding. Because a notable difference between the two groups became evident at a radiation dose of 6 Gy, further experiments focused exclusively on the 6 Gy radiation dose.

We repeated this study in organoids derived from Lgr5/GFP-IRES-Cre-ERT2; R26-ACTB-tdTomato-EGFPknock-in mice where LGR5+ ISCs could be detected based on EGFP expression. In non-irradiated conditions, we observed no significant difference in the ratio of GFP+ budding organoids to total organoids between male and female organoids. However, a noticeable sex-based difference emerged within 48 h post irradiation in male organoids. We noticed a reduction in the population of Lgr5+ve ISCs, resulting in a significant loss of GFP+ budding crypts with alterations in the morphology of existing crypts, indicative of the inhibition of ISC growth and differentiation in response to the 6 Gy radiation exposure. In contrast, female mouse organoids exhibited a significantly higher GFP+ budding organoid/total organoid ratio, highlighting their radioresistance property (*p* < 0.005) (Figure 5E,F). These findings highlight that female mice ISCs are more resistant to radiation-induced injury.

### 3.7. Female Intestinal Organoid Mimics the In Vivo Characteristics

**Figure 6:**
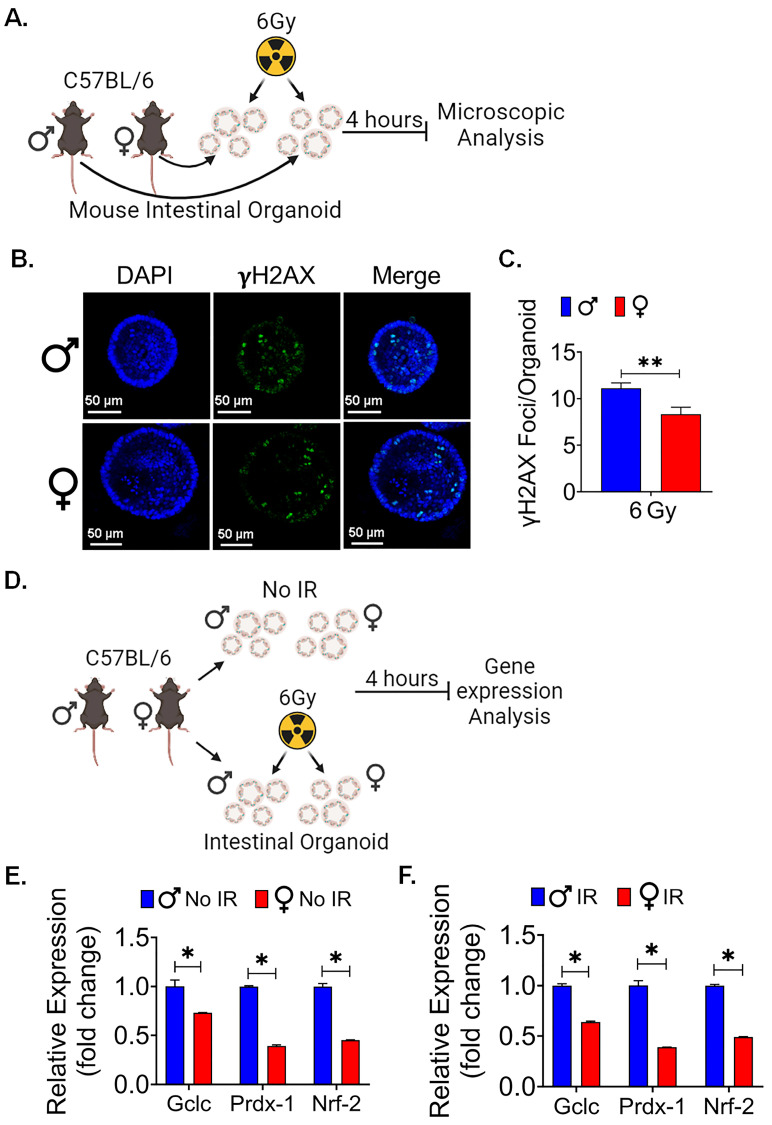
Female intestinal organoids are resistant to radiation-induced DNA damage: (**A**) Schematic representation of male and female mice intestinal organoids culture and radiation exposure for microscopic analysis. (**B**,**C**) A significantly higher number of γH2AX foci was observed in male mice intestinal organoids when compared to female mice, highlighting sex-based differences in the response to DNA damage induced by radiation exposure. (**D**) Schematic representation of male and female mice intestinal organoids culture and radiation exposure for gene expression analysis. (**E**,**F**) The qPCR analysis showed significantly upregulated genes of oxidative stress-responsive gene *Gclc*, *Prdx*, and *Nrf2* expression in non-irradiated and irradiated male organoids when compared to female mice organoids. Data presented as the mean ± SD. (Significance level, *: *p* < 0.05, **: *p* < 0.005).

We further examined the comparison between male and female organoids in DNA damage markers following irradiation (Figure 6A). We observed that the DNA damage marker in mouse intestinal organoid exposed to irradiation showed a significant difference between male and female organoids. At a radiation dose of 6 Gy, we observed a significantly higher number of γH2AX foci in male mice intestinal organoids than in female mice (*p* < 0.005) (Figure 6B,C).

Additionally, when examining oxidative stress gene expression, consistent with our in vivo findings, we found a significant difference in expression profiles. qPCR analysis of oxidative stress markers showed significantly upregulated gene expression of *Gclc*, *Prdx-1*, and *Nrf-2* (*p* < 0.05 for all) in non-irradiated and irradiated male organoids in comparison to the female mice organoids (Figure 6D–F).

All these ex vivo findings collectively suggest that differences in the ability of LGR5+ cells to respond to radiation are not solely attributed to hormonal variations or distal organ interactions encountered in an in vivo setting. Moreover, they pinpoint the male-specific surge in oxidative stress as a post irradiation phenomenon, as male organoids displayed higher basal levels of oxidative stress even in the absence of radiation exposure.

### 3.8. Human Intestinal Organoids Derived from Females Are Radioresistant Compared to Those Derived from Males

**Figure 7:**
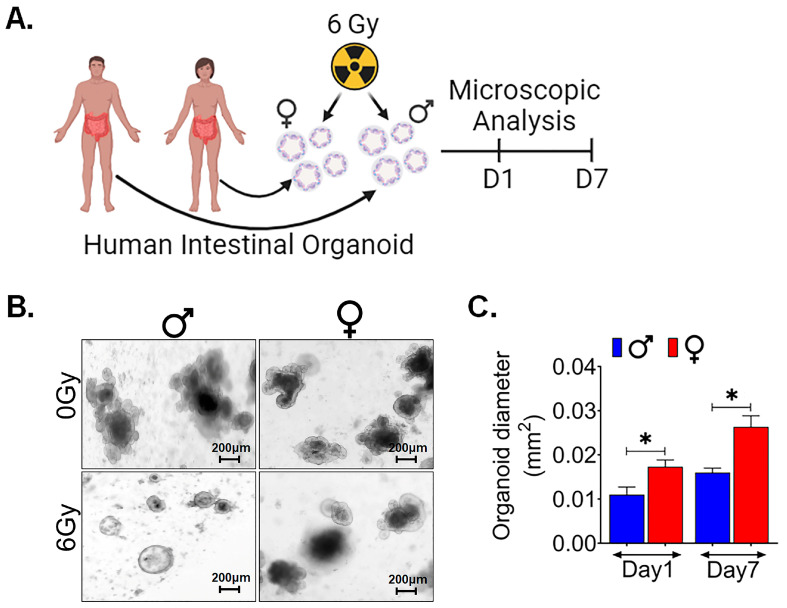
Female intestinal organoids show higher radioresistance: (**A**) Schematic representation of human male and female intestinal crypt organoids culture, radiation exposure for their morphological features by microscopic examining. (**B**,**C**) The assessment of the average cross-sectional area of female organoids was significantly higher at day 1 and 7 following radiation exposure when compared to male organoids, highlighting a sex-based differences in the post-radiation responses. (*n* = 5 human samples per group). Data presented as the mean ± SD. (Significance level, *: *p* < 0.05).

In order to validate the sex-specific differences observed in murine models, we sought to investigate whether these findings could be replicated in human tissues. To accomplish this, we utilized male and female surgical specimens obtained from normal colon regions, positioned at least 10 cm away from malignant sites [43]. From these specimens, we generated ex vivo crypt organoids and subjected them to radiation exposure at a dose level of 6 Gy (Figure 7A). Interestingly, in human male organoids, all budding crypts were found to have disappeared following exposure to 6 Gy, in contrast to the human female organoids (Figure 7B). Female organoids exhibited a significantly larger cross-sectional area at both day 1 (*p* < 0.05) and day 7 (*p* < 0.05) post exposure (Figure 7C). These results are very similar to what we observed in female murine organoids, thereby indicating consistency in outcomes across species.

### 3.9. RNAseq Analysis of Human LGR5+ Cells Shows Sex-Dependent Differences in Mitochondrial Gene Expression

**Figure 8:**
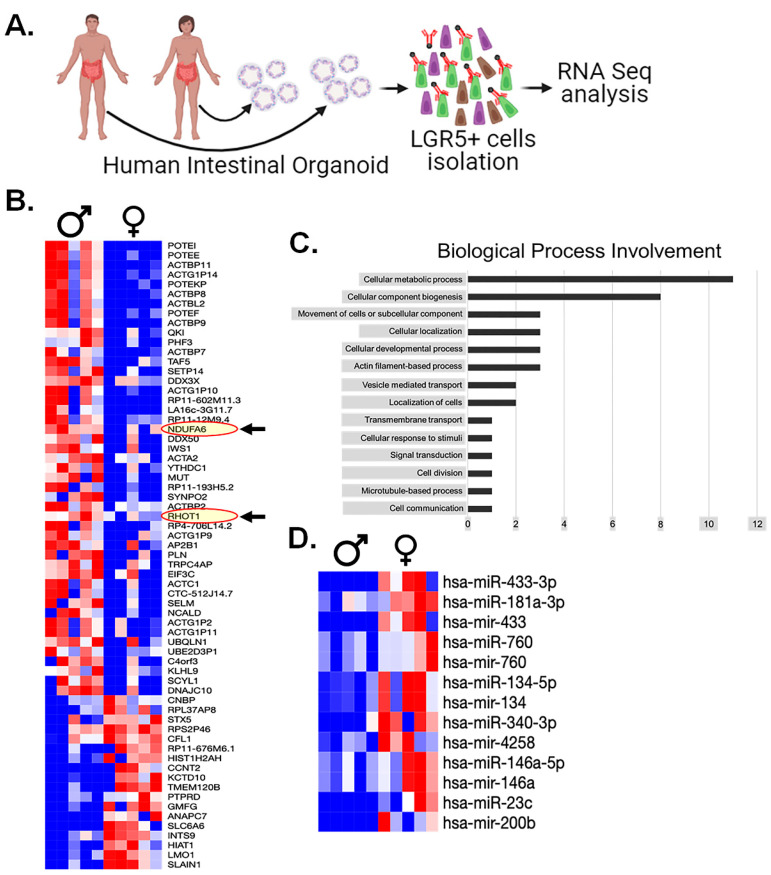
Transcriptomics analysis: (**A**). Experimental design for patient-derived organoids culture, isolation of LGR5+ cells for RNA seq analysis. (**B**) Differential mRNA expression in male and female LGR5+ cells. RNA seq. analysis revealed significant differences in the expression of 55 mRNA genes, highlighting that two mitochondrial proteins, NDUFA6 (an Electron Transport Chain protein) and RHOT1 (a mitochondrial membrane protein), were upregulated in males compared to females (shown in red oval with arrow). (**C**) The gene ontology analysis of differentially expressed genes categorized the biological processes associated with the 55 differentially expressed genes, and (**D**) the miRNA analysis of serum samples showed 13 miRNAs that exhibited statistically significant differences between males and females (*n* = 5 human samples per group).

To gain deeper understanding into the underlying factors contributing to disparities in radiosensitivity of the LGR5+ cell population between males and females, we conducted a comprehensive mRNA expression analysis using patient-derived organoids (PDOs). Specifically, PDOs were generated from tissue samples obtained from 10 patients (five males and five females) (Figure 8A). LGR5+ cells were isolated from intestinal organoids employing the magnetic microbeads method and were processed for RNA sequencing analysis (Figure 8A).

The RNAseq analysis revealed substantial disparities in the expression levels of 55 mRNA genes (Figure 8B). Interestingly, among these findings, we observed the upregulation of two mitochondrial proteins, namely NDUFA6 (an Electron Transport Chain protein) and RHOT1 (a mitochondrial membrane protein) in males compared to females (Figure 8B). To systematically categorize the implications of these mRNA findings, we subjected this gene list to a gene ontology analysis, resulting in the involvement of specific biological processes (Figure 8C).

Remarkably, one of the most notable findings we observed from this analysis is the absence of measurable differences in DDR machinery expression between the sexes. Instead, the predominant category pertains to metabolic processes, supporting our previous observations of oxidative stress. This collective evidence points toward mitochondrial factors as the key determinants underlying our findings between males and females.

### 3.10. miRNA Analysis of Human Serum Highlights Several Biomarkers

Finally, in an attempt to further explain any observed differences in human LGR5+ populations, we performed a miRNA analysis of serum samples from the same male and female patients whose intestinal surgical samples were used to generate the intestinal organoids. The miRNA analysis yielded a collection of 13 miRNAs that exhibited statistically significant distinctions across all males and females studied (Figure 8D). Interestingly, among these, several miRNAs had previously been explored for their potential roles in modulating radiosensitivity. Out of 2079 identified mi-RNA from the plasma of males and females, 37 mi-RNAs were associated with mitochondrial biogenesis (Supplementary Tables S2 and S3). These finding shows that some of these miRNAs may play a role in modulating the mitochondrial biogenesis process and enhancing the response to radiation resistance, especially in females. Understanding the specific roles of these miRNAs is essential for understanding the mechanisms behind the observed sex-based differences in radioresistance.

Nonetheless, hsa-mir-760 displayed significantly higher expression levels in female serum samples (*p* < 0.05). It is noteworthy that previous investigations have linked low hsa-mir-760 expression to increased cell proliferation in colorectal tissue in in vitro [44]. This interesting finding suggests that hsa-mir-760 may play a pivotal role in modulating cell proliferation, with potential implications for the observed differences in radiosensitivity.

Given these findings, it is evident that further research is needed to investigate the individual functions of these miRNAs in the context of mitochondrial biogenesis and their impact on radioresistance. These studies will be crucial in unveiling the intricate molecular mechanisms that underlie the differences in radiosensitivity among human LGR5+ crypt cell populations. The complexity of miRNA regulation and its multifaceted roles necessitate a deeper exploration to decipher the precise functions and contributions of these specific miRNAs in the context of radiosensitivity.

## 4. Discussion

In this study, we investigated the potential disparities in the radiosensitivity of male and female intestinal epithelium, as well as unraveled the underlying mechanisms contributing to these differences. We identified a significant radiosensitivity difference in male and female intestinal epithelium and LGR5+ stem cell populations in both murine and human tissue. Specifically, female LGR5+ ISCs displayed enhanced radioresistance and capacity for proliferation following radiation exposure. This robustness of female LGR5+ ISCs is of particular importance since ISCs play a pivotal role in preserving the homeostasis and regenerative potential of intestinal tissue. Interestingly, our study suggested that the sex-dependent differences in ISC radiosensitivity may be due to sex-dependent differences in mitochondrial function.

RNAseq analysis of Lgr5+ ISCs and serum miRNA analysis provide an understanding of the molecular differences underlying the radioresistance exhibited by females. Notably, biological processes such as oxidative stress, mitochondrial biogenesis, cellular metabolism, and stemness markers are involved in sex-dependent differences in intestinal radiosensitivity. It is well reported that radiation response upregulates the oxidative stress [45,46], which is one of the key factors we also observed in our study. Our analysis of oxidative stress gene expression showed increased oxidative stress in male intestinal epithelial cells following irradiation compared to female. This upregulation in oxidative stress, especially after radiation exposure, is a critical factor contributing to the enhanced sensitivity of male mice to radiation-induced damage compared to female mice. In contrast, female mouse intestinal epithelial cells display lower expression of oxidative phosphorylation (OXPHOS) proteins and genes associated with mitochondrial biogenesis, specifically *NRF-1* and *TFAM*. This observation suggests that female intestinal epithelial cells undergo a metabolic shift away from OXPHOS and towards glycolysis. This metabolic modulation supports the concept that glycolysis supports stemness in cells [47]. This shift potentially enhances the maintenance of stem cell characteristics [48] in female subjects, providing an additional layer of protection against radiation-induced injury. This observation is supported by the notably higher expression of major stem cell markers, including *LGR5* and *BMI1*, in female intestinal epithelial cells. These findings collectively emphasize the stemness maintained by female cells, which contributes to their radioresistance properties.

It is worth highlighting that the modulation of metabolic pathways, including the shift toward glycolysis, has been recognized as a strategy employed by various stem cell populations to sustain their undifferentiated state [49,50]. In our study, considering this concept for the LGR5+ population of intestinal epithelial cells to maintain their stemness and regenerative capacity in the presence of radiation-induced stress was a key factor contributing to the overall radio resistance of the female intestinal epithelium. The immunofluorescent staining for γ-H2AX indicated that male mice exhibited a greater extent of radiation-induced DNA damage. These findings contribute to the overall understanding of why male mice are more susceptible to radiation-induced injury. Moreover, the activation of WNT β-catenin signaling was more significant in female intestinal epithelial cells, indicating a potential sex-specific difference in the response to WNT pathway activation, which is vital for tissue repair and regeneration.

The findings of our study demonstrate convincing evidence for the radioresistance of ex vivo intestinal organoid cultures from female mice. These organoids have emerged as a valuable model for investigating the biology of ISCs, and our experiments provided critical insights into sex-based differences in radiation response. Specifically, our data indicate that female organoids exhibit remarkable radioresistance, with a significantly higher budding crypt ratio. In contrast, male organoids displayed pronounced sensitivity to radiation, as evidenced by the reduction in budding crypts and alterations in crypt morphology following exposure to 6 Gy. Moreover, the use of genetically modified mouse Lgr5/GFP-IRES-Cre-ERT2; R26-ACTB-tdTomato-EGFPknock-in allowed us to visualize and quantify LGR5+ intestinal stem cells based on EGFP expression which shows a sex-specific differences within 48 h post irradiation. These differences can become crucial for considering radiation therapy and offer potential strategies for enhancing the protection of healthy tissues during such treatments.

Our findings using human organoids supported the observation made in murine models, highlighting the resistance to radiation toxicity in female intestinal epithelial cells compared to male. These human organoid models also provided insights into the sex-specific differences in DNA damage markers following irradiation. The RNA sequencing analysis of human LGR5+ cells revealed differences in the expression of several genes, notably the upregulation of mitochondrial proteins in males, suggesting the involvement of mitochondrial factors as key determinants of radiosensitivity. Importantly, this analysis did not show measurable differences in DNA damage response machinery between the sexes. The miRNA analysis of human serum samples identified several miRNAs with statistically significant distinctions between males and females. The identified miRNA may play a critical role in modulating cell proliferation and could contribute to the observed differences in radiosensitivity. It has been reported that female mice displayed a greater increase in G-CSF levels and a lesser increase in miR-34a in GI than male mice after irradiation. MiR-34a is known to increase p53 activation, which leads to apoptosis [51]. It should be borne in mind that G-CSF suppressed miR-34a and increased the Bcl-2/Bax ratio and lowered the MAPK activation in GI [13]. This G-CSF/miR-34a signaling pathway also contributed to the sex-dependent disparity in radiosensitivity in addition to the lower oxidative stress found in the females presented in this study.

In summary, the findings of this study provide a comprehensive understanding of the sex-based differences in radiosensitivity to intestinal epithelium. These findings have important implications for radiation therapy and highlight the need for personalized treatment strategies based on sex-specific differences in radiosensitivity. Further research into the specific roles of the identified genes, proteins, miRNAs, and metabolic pathways will be essential for a deeper understanding of the mechanisms driving these differences and their potential clinical applications.

## Data Availability

Data is contained within the article and Supplementary Material.

## Supplementary Materials

The following supporting information can be downloaded at: https://www.mdpi.com/article/10.3390/cells13010046/s1, Figure S1: Western blot: Total OXPHOS; Table S1: Primer Sequences, Table S2: has-miR identification between male vs. Female, Table S3: mi-RNAs associated with mitochondrial biogenesis.
