# Supplementary material for: LGR5+ Intestinal Stem Cells Display Sex-Dependent Radiosensitivity
Source: Cells. 2023 Dec 25;13(1):46. doi: 10.3390/cells13010046 (PMC10778194; doi:10.3390/cells13010046)

Supplementary Table-S1 Primer Sequences

Gene	Forward Sequence	Reverse Sequence
Gclc	ACACCTGGATGATGCCAACGAG	CCTCCATTGGTCGGAACTCTAC
Prdx-1	TGCCAAGTGATTGGCGCTTCTG	AGCAATGGTGCGCTTGGGATCT
Nrf-2	CAGCATAGAGCAGGACATGGAG	GAACAGCGGTAGTATCAGCCAG
NqO1	GCCGAACACAAGAAGCTGGAAG	GGCAAATCCTGCTACGAGCACT
TFAM	GAGGCAAAGGATGATTCGGCTC	CGAATCCTATCATCTTTAGCAAGC
NRF-1	GGCAACAGTAGCCACATTGGCT	GTCTGGATGGTCATTTCACCGC
LGR5	AGAGCCTGATACCATCTGCAAAC	TGAAGGTCGTCCACACTGTTGC
BMI-1	ACTACACGCTAATGGACATTGCC	CTCTCCAGCATTCGTCAGTCCA
LEF-1	ACTGTCAGGCGACACTTCCATG	GTGCTCCTGTTTGACCTGAGGT
OLFM4	GCCTCCAAAAGTGACCTTGTGC	TGCGTGTGCTGGTGGAAAAGAG
AXIN-1	GTCCAGTGATGCTGACACGCTA	GCCCATTGACTTGGATACTCTCC
ASCL2	TTTCCTGTGCCGCACCAGAACT	CAGCGACTCCAGACGAGGTGG
TCF-4	CCTCCAATCCTTCAACTCCTGTG	TCCAAACGGTCTTCGATTCGGC
SOX-	CACACGTCAAGCGACCCATGAA	TCTTCTCGCTCTCGTTCAGCAG
GAPDH	ACCACAGTCCATGCCATCAC	TCCACCACCCTGTTGCTGTA

Supplementary Table-S2

					Female Sample ID					Male Sample ID					
gene_id	chrom	start	stop	strand	_22564_re	_22676_re	_25114_re	_26512_re	_28700_re	_24793_re	_25097_re	_26534_re	_27046_re	_27133_re	type
hsa-miR-320a-3p	chr8	22102488	22102509	-	183797	111607	951241	71618	171395	60179	195518	6391	362529	255166	miRNA
hsa-mir-320a	chr8	22102479	22102550	-	183797	111607	951241	71618	171395	60179	195518	6391	362529	255168	miRNA
hsa-mir-486-2	chr8	41517962	41518025	+	92655	98852	1497030	64970	106967	58978	557526	7967	1206118	246375	miRNA
hsa-mir-486-1	chr8	41517959	41518026	-	92059	98626	1496759	64745	106665	58652	557063	7994	1205459	246398	miRNA
hsa-miR-486-5p	chr8	41517962	41517983	+	91357	98370	1492567	64706	105440	58411	553311	7924	1200321	244956	miRNA
hsa-miR-21-5p	chr17	57918634	57918655	+	60486	134044	398795	66378	232010	81158	66058	7852	100529	176555	miRNA
hsa-mir-21	chr17	57918627	57918698	+	60486	134110	399014	66438	232058	81245	66058	7893	100601	176688	miRNA
hsa-miR-103a-3p	chr20	3898188	3898210	+	32856	35875	167298	17024	64682	23242	56118	2682	127267	93965	miRNA
hsa-mir-103a-1	chr5	1.68E+08	1.68E+08	-	32856	35875	167298	17024	64682	23242	56118	2682	127267	93965	miRNA
hsa-mir-103a-2	chr20	3898141	3898218	+	32844	35859	167307	17015	64672	23221	56163	2678	127277	93941	miRNA
hsa-mir-423	chr17	28444097	28444190	+	30944	9513	133265	8693	20621	8804	46929	1096	87391	24616	miRNA
hsa-miR-221-3p	chrX	45605608	45605630	-	30256	27524	178393	12521	68030	15198	14783	2139	22676	55479	miRNA
hsa-mir-221	chrX	45605585	45605694	-	30256	27524	178393	12521	68036	15198	14783	2139	22676	55479	miRNA
hsa-miR-423-5p	chr17	28444113	28444135	+	29936	8659	131630	8406	18520	7729	46523	935	86103	23314	miRNA
hsa-mir-25	chr7	99691183	99691266	-	20535	15026	237555	11826	56007	13668	69558	1461	153994	80348	miRNA
hsa-miR-25-3p	chr7	99691194	99691215	-	20383	15026	237187	11826	55935	13615	69380	1461	153505	80267	miRNA
hsa-miR-26b-5p	chr2	2.19E+08	2.19E+08	+	19253	20503	13676	7714	55368	12673	14058	1016	12600	37177	miRNA
hsa-mir-26b	chr2	2.19E+08	2.19E+08	+	19253	20503	13676	7714	55375	12673	14058	1016	12600	37177	miRNA
hsa-miR-107	chr10	91352513	91352535	-	18383	15062	158278	8066	20035	10882	50985	1621	120891	64140	miRNA
hsa-mir-107	chr10	91352504	91352584	-	18383	15062	158278	8066	20046	10882	50985	1621	120891	64140	miRNA
hsa-mir-24-2	chr19	13947101	13947173	-	17512	13489	25083	10428	51012	10694	7857	1664	8566	25972	miRNA
hsa-miR-24-3p	chr19	13947103	13947124	-	17416	13372	25014	10415	50765	10677	7847	1663	8548	25782	miRNA
hsa-mir-24-1	chr9	97848303	97848370	+	17416	13372	25014	10415	50765	10677	7847	1663	8548	25782	miRNA
hsa-mir-16-2	chr3	1.6E+08	1.6E+08	+	14976	9780	254165	7595	34810	10476	91015	1320	161638	56189	miRNA
hsa-miR-16-5p	chr13	50623163	50623184	-	14109	8753	241044	7009	33493	9826	86641	1172	154996	52947	miRNA
hsa-mir-16-1	chr13	50623109	50623197	-	14074	8728	240172	6996	33423	9813	86319	1168	154545	52795	miRNA
hsa-mir-92a-1	chr13	92003568	92003645	+	12278	18446	176752	11916	28057	11733	56222	2525	134776	38959	miRNA
hsa-miR-122-5p	chr18	56118320	56118341	+	12224	6174	38500	14008	170604	22219	26454	278	2150	29190	miRNA
hsa-mir-122	chr18	56118306	56118390	+	12224	6174	38500	14008	170615	22219	26454	278	2150	29190	miRNA
hsa-miR-451a	chr17	27188421	27188442	-	11493	7472	161405	5997	11913	6674	52936	1133	97842	36653	miRNA
hsa-mir-451a	chr17	27188387	27188458	-	11493	7490	161943	5997	11960	6715	53224	1133	98027	36749	miRNA
hsa-miR-92a-3p	chr13	92003615	92003636	+	11245	17860	176211	11432	27076	11328	55790	2212	134292	38250	miRNA
hsa-mir-92a-2	chrX	1.33E+08	1.33E+08	-	11245	17860	176211	11432	27076	11328	55790	2212	134292	38250	miRNA
hsa-miR-93-5p	chr7	99691438	99691460	-	11151	8267	31441	4172	30723	6119	15136	901	32874	44289	miRNA
hsa-mir-93	chr7	99691391	99691470	-	11151	8267	31470	4172	30773	6122	15136	901	32923	44289	miRNA
hsa-mir-26a-2	chr12	58218392	58218475	-	11107	10441	6403	4722	25541	7098	3274	2032	4022	13699	miRNA
hsa-miR-26a-5p	chr12	58218441	58218462	-	10980	10384	6334	4671	25318	7087	3230	2035	4006	13564	miRNA
hsa-mir-26a-1	chr3	38010895	38010971	+	10980	10384	6334	4671	25318	7087	3230	2035	4006	13564	miRNA
hsa-miR-30d-5p	chr8	1.36E+08	1.36E+08	-	10666	10849	44500	7093	30162	7174	13687	1002	22087	20981	miRNA
hsa-mir-30d	chr8	1.36E+08	1.36E+08	-	10666	10849	44500	7093	30179	7174	13733	1002	22133	20981	miRNA
hsa-mir-101-1	chr1	65524117	65524191	-	10490	18119	137478	9591	17758	9397	40081	1596	112528	48655	miRNA
hsa-miR-101-3p	chr1	65524125	65524145	-	10475	18199	137855	9583	18003	9400	40321	1595	113109	49105	miRNA
hsa-mir-101-2	chr9	4850297	4850375	+	10475	18199	137855	9583	18003	9400	40321	1595	113127	49105	miRNA
hsa-miR-22-3p	chr17	1617208	1617229	-	10344	5223	122394	9284	14822	7600	36460	3454	48263	21988	miRNA
hsa-mir-22	chr17	1617197	1617281	-	10344	5249	122710	9284	15015	7747	36504	3454	48493	22165	miRNA
hsa-miR-199a-3p	chr1	1.72E+08	1.72E+08	-	9103	8091	15252	7508	37062	7990	3622	1178	4011	12797	miRNA
hsa-miR-199b-3p	chr9	1.31E+08	1.31E+08	-	9103	8091	15252	7508	37062	7990	3622	1178	4011	12797	miRNA
hsa-mir-199a-1	chr19	10928102	10928172	-	9103	8091	15275	7508	37112	7990	3622	1178	4011	12797	miRNA
hsa-mir-199b	chr9	1.31E+08	1.31E+08	-	9103	8091	15262	7508	37083	7990	3622	1179	4011	12797	miRNA
hsa-mir-199a-2	chr1	1.72E+08	1.72E+08	-	9095	8087	15263	7500	37118	7984	3617	1177	4004	12788	miRNA
hsa-miR-15a-5p	chr13	50623303	50623324	-	8466	5307	145692	2808	9405	5025	44651	456	75022	27517	miRNA
hsa-mir-15a	chr13	50623255	50623337	-	8466	5307	145693	2808	9405	5025	44651	456	75022	27517	miRNA
hsa-mir-19b-1	chr13	92003446	92003532	+	7657	6242	27054	3600	6182	3656	8758	794	24588	20467	miRNA
hsa-miR-19b-3p	chr13	92003499	92003521	+	7519	6246	27064	3602	6185	3658	8768	794	24603	20474	miRNA
hsa-mir-19b-2	chrX	1.33E+08	1.33E+08	-	7519	6246	27064	3602	6185	3658	8768	794	24603	20474	miRNA
hsa-mir-223	chrX	65238712	65238821	+	6847	3652	5882	2310	31045	2640	1710	365	675	11119	miRNA
hsa-mir-140	chr16	69966984	69967083	+	6639	2721	21613	2636	8251	2964	6562	539	15737	10812	miRNA
hsa-miR-140-3p	chr16	69967045	69967065	+	6379	2537	20910	2493	7549	2670	6238	519	15582	9908	miRNA
hsa-miR-223-3p	chrX	65238779	65238800	+	6252	3322	5271	1804	28389	2420	1661	365	604	9842	miRNA
hsa-mir-181a-1	chr1	1.99E+08	1.99E+08	-	6249	4850	21697	3257	12645	4425	4598	1209	9670	9696	miRNA
hsa-mir-181a-2	chr9	1.27E+08	1.27E+08	+	6239	4904	21695	3306	12745	4396	4661	1205	9724	9795	miRNA
hsa-miR-320b	chr1	1.17E+08	1.17E+08	+	6198	3627	104593	2540	5972	2155	6732	290	12002	6355	miRNA
hsa-miR-320b-2	chr1	2.24E+08	2.24E+08	-	6198	3627	104593	2540	5972	2155	6732	290	12002	6355	miRNA
hsa-miR-181a-5p	chr1	1.99E+08	1.99E+08	-	6174	4772	21608	3221	12535	4396	4553	1205	9646	9636	miRNA
hsa-miR-378a-3p	chr5	1.49E+08	1.49E+08	+	6151	2476	14184	1908	7393	1381	4741	412	9025	7858	miRNA
hsa-mir-378a	chr5	1.49E+08	1.49E+08	+	6151	2476	14184	1908	7399	1381	4741	412	9044	7858	miRNA
hsa-mir-320b-1	chr1	1.17E+08	1.17E+08	+	6023	3666	104048	2617	5904	2211	6343	293	11706	6232	miRNA
hsa-mir-106b	chr7	99691616	99691697	-	5137	2790	20635	2456	5322	1845	8149	378	17182	13634	miRNA
hsa-miR-185-5p	chr22	20020676	20020697	+	5031	1921	20952	1359	4995	2095	13784	186	19605	7551	miRNA
hsa-mir-185	chr22	20020662	20020743	+	5031	1989	21068	1446	5080	2132	13821	187	19672	7640	miRNA
hsa-mir-142	chr17	56408593	56408679	-	4782	3789	17784	2695	8384	3078	8239	959	9284	9294	miRNA
hsa-mir-30e	chr1	41220027	41220118	+	4750	2417	29341	3506	7853	3028	9148	653	12674	8386	miRNA
hsa-miR-30e-5p	chr1	41220043	41220064	+	4650	2254	29137	3391	7347	3021	9039	437	12535	8039	miRNA
hsa-miR-142-5p	chr17	56408644	56408664	-	4291	3416	16981	2263	7082	2328	7765	666	8564	7397	miRNA
hsa-mir-144	chr17	27188551	27188636	-	4290	7090	42779	3278	4803	2243	19327	69	14328	19207	miRNA
hsa-mir-191	chr3	49058051	49058142	-	3745	659	989	1343	2106	1274	327	1181	792	2164	miRNA
hsa-mir-151a	chr8	1.42E+08	1.42E+08	-	3693	1902	5691	2593	6506	2167	1348	554	2659	4278	miRNA
hsa-mir-148a	chr7	25989539	25989606	-	3685	2083	8299	1896	6062	1331	2222	273	3807	3872	miRNA
hsa-miR-106b-3p	chr7	99691625	99691646	-	3657	2037	14896	2025	3683	1413	6029	349	12313	9831	miRNA
hsa-miR-191-5p	chr3	49058105	49058127	-	3648	642	965	1221	2092	1274	327	1181	792	2128	miRNA
hsa-miR-146a-5p	chr5	1.6E+08	1.6E+08	+	3606	1402	2811	2100	3752	1680	691	1090	1503	2152	miRNA

hsa-mir-146a	chr5	1.6E+08	1.6E+08	+	3606	1402	2811	2100	3753	1680	691	1090	1503	2152	miRNA
hsa-miR-744-5p	chr17	11985226	11985247	+	3592	3228	2038	1734	7571	2984	1534	203	1763	4673	miRNA
hsa-mir-744	chr17	11985216	11985313	+	3592	3228	2038	1736	7607	2984	1534	203	1763	4673	miRNA
hsa-miR-155-5p	chr21	26946295	26946318	+	3496	2498	981	1941	1980	1070	1561	1755	1686	4357	miRNA
hsa-mir-155	chr21	26946292	26946356	+	3496	2498	981	1941	1980	1070	1561	1755	1686	4357	miRNA
hsa-miR-148a-3p	chr7	25989542	25989563	-	3491	2033	8242	1896	5866	1315	2222	273	3807	3752	miRNA
hsa-miR-17-5p	chr13	92002872	92002894	+	3326	2210	2813	921	6237	1324	2176	0	3940	6084	miRNA
hsa-mir-17	chr13	92002859	92002942	+	3326	2281	4753	976	6373	1385	2748	0	4548	6531	miRNA
hsa-miR-106a-5p	chrX	1.33E+08	1.33E+08	-	3311	1863	2737	843	538	1287	2106	0	4606	5921	miRNA
hsa-mir-106a	chrX	1.33E+08	1.33E+08	-	3311	1863	2778	843	538	1288	2106	0	4656	5921	miRNA
hsa-miR-143-3p	chr5	1.49E+08	1.49E+08	+	3224	4147	7371	2518	8158	1612	1582	509	1296	5449	miRNA
hsa-mir-143	chr5	1.49E+08	1.49E+08	+	3224	4147	7371	2518	8176	1612	1582	509	1296	5449	miRNA
hsa-miR-144-3p	chr17	27188566	27188585	-	3103	2148	30370	1917	2240	1507	13072	69	11111	13596	miRNA
hsa-miR-98-5p	chrX	53583260	53583281	-	2768	7743	7568	2785	16207	3658	4703	425	4072	10895	miRNA
hsa-mir-98	chrX	53583184	53583302	-	2768	7743	7568	2785	16207	3658	4703	425	4083	10895	miRNA
hsa-miR-151a-3p	chr8	1.42E+08	1.42E+08	-	2696	1531	4857	2291	4984	1702	1212	486	2358	3647	miRNA
hsa-mir-27a	chr19	13947254	13947331	-	1799	2281	5591	1024	3614	965	1570	366	709	2399	miRNA
hsa-miR-20a-5p	chr13	92003326	92003348	+	1761	2529	6823	974	6079	1420	4282	53	9122	9455	miRNA
hsa-mir-20a	chr13	92003319	92003389	+	1761	2529	6823	974	6086	1420	4282	53	9122	9455	miRNA
hsa-miR-27a-3p	chr19	13947261	13947281	-	1726	2281	5591	1024	3569	965	1570	366	699	2360	miRNA
hsa-mir-320c-1	chr18	19263479	19263550	+	1539	940	15269	700	2458	576	1742	208	3994	1713	miRNA
hsa-miR-106b-5p	chr7	99691666	99691686	-	1480	753	5739	431	1639	432	2120	29	4869	3803	miRNA
hsa-miR-320c	chr18	19263520	19263539	+	1375	988	15493	755	2472	595	1786	209	3957	1777	miRNA
hsa-mir-320c-2	chr18	21901639	21901710	+	1375	988	15493	755	2472	595	1786	209	3957	1777	miRNA
hsa-mir-126	chr9	1.4E+08	1.4E+08	+	1340	3319	4469	2181	6713	3444	2629	410	2527	2953	miRNA
hsa-miR-146b-5p	chr10	1.04E+08	1.04E+08	+	1227	622	277	698	972	401	481	697	203	848	miRNA
hsa-mir-146b	chr10	1.04E+08	1.04E+08	+	1227	622	299	698	1001	443	493	697	249	848	miRNA
hsa-miR-27b-3p	chr9	97847787	97847807	+	1226	1672	5368	1235	3161	882	1400	565	1225	1852	miRNA
hsa-mir-27b	chr9	97847727	97847823	+	1226	1672	5368	1235	3215	882	1400	565	1250	1896	miRNA
hsa-miR-144-5p	chr17	27188601	27188622	-	1187	4942	12409	1361	2563	736	6255	0	3239	5611	miRNA
hsa-mir-30a	chr6	72113254	72113324	-	1180	2077	8516	1196	3270	1525	2332	1096	719	2906	miRNA
hsa-miR-30a-5p	chr6	72113298	72113319	-	1178	2020	8387	1195	3144	1483	2316	1026	696	2839	miRNA
hsa-miR-4508	chr15	23807254	23807270	-	1169	1546	6329	815	109	802	4774	35	1186	4270	miRNA
hsa-mir-4508	chr15	23807209	23807278	-	1169	1546	6329	815	109	802	4774	35	1186	4270	miRNA
hsa-miR-432-5p	chr14	1.01E+08	1.01E+08	+	1144	751	288	3224	4671	1106	189	92	681	138	miRNA
hsa-mir-432	chr14	1.01E+08	1.01E+08	+	1144	751	288	3224	4671	1106	189	92	681	138	miRNA
hsa-miR-12136	chr1	568048	568065	-	1106	761	216	1252	1940	243	246	0	341	2886	miRNA
hsa-mir-12136	chr1	567995	568065	-	1106	777	216	1252	1940	243	246	0	341	2886	miRNA
hsa-miR-340-5p	chr5	1.79E+08	1.79E+08	-	1056	831	1247	247	908	887	389	0	622	1512	miRNA
hsa-mir-340	chr5	1.79E+08	1.79E+08	-	1056	893	1297	321	977	926	389	0	622	1513	miRNA
hsa-mir-28	chr3	1.88E+08	1.88E+08	+	1026	919	941	879	2271	727	216	530	466	1471	miRNA
hsa-miR-28-3p	chr3	1.88E+08	1.88E+08	+	1016	695	785	762	1423	587	195	482	310	1107	miRNA
hsa-miR-423-3p	chr17	28444149	28444171	+	1008	854	1635	287	2101	1075	406	161	1288	1302	miRNA
hsa-miR-151a-5p	chr8	1.42E+08	1.42E+08	-	997	371	834	302	1522	465	136	68	301	631	miRNA
hsa-miR-3615	chr17	72744802	72744822	+	994	1227	5491	217	338	253	1274	74	2963	4235	miRNA
hsa-mir-3615	chr17	72744752	72744838	+	994	1227	5491	217	338	253	1274	74	2963	4235	miRNA
hsa-miR-134-5p	chr14	1.02E+08	1.02E+08	+	965	149	432	859	946	282	0	90	49	118	miRNA
hsa-mir-134	chr14	1.02E+08	1.02E+08	+	965	149	432	859	946	282	0	90	49	118	miRNA
hsa-mir-320d-1	chr13	41301952	41302023	-	957	613	6926	449	1880	252	1101	140	2851	1203	miRNA
hsa-miR-320d	chr13	41301964	41301982	-	915	569	6120	425	1825	234	899	139	2703	1022	miRNA
hsa-mir-320d-2	chrX	1.4E+08	1.4E+08	-	915	569	6120	425	1825	234	899	139	2703	1022	miRNA
hsa-miR-130a-3p	chr11	57408725	57408746	+	901	628	8540	334	764	534	1227	142	590	798	miRNA
hsa-mir-130a	chr11	57408671	57408759	+	901	628	8540	334	777	534	1228	142	590	798	miRNA
hsa-miR-186-5p	chr1	71533364	71533385	-	896	418	1816	499	948	668	1026	188	1223	1729	miRNA
hsa-mir-186	chr1	71533314	71533399	-	896	418	1816	499	948	668	1026	188	1223	1729	miRNA
hsa-miR-16-2-3p	chr3	1.6E+08	1.6E+08	+	867	1027	13121	586	1317	650	4374	148	6642	3242	miRNA
hsa-miR-126-5p	chr9	1.4E+08	1.4E+08	+	839	1996	3452	1479	2199	1840	1637	365	1720	1721	miRNA
hsa-mir-425	chr3	49057581	49057667	-	709	539	3610	469	1374	511	1963	249	2441	1539	miRNA
hsa-miR-486-3p	chr8	41517961	41517981	-	703	235	4106	39	1152	241	3628	70	4979	1328	miRNA
hsa-mir-130b	chr22	22007593	22007674	+	679	503	8757	467	940	365	1321	286	1647	1138	miRNA
hsa-miR-99b-5p	chr19	52195871	52195892	+	664	38	128	0	19	127	0	57	16	41	miRNA
hsa-mir-99b	chr19	52195865	52195934	+	664	80	160	149	97	178	0	57	40	95	miRNA
hsa-mir-941-2	chr20	62550858	62550929	+	648	130	264	0	371	0	202	78	340	0	miRNA
hsa-mir-941-3	chr20	62550914	62550985	+	648	130	264	0	371	0	202	78	340	0	miRNA
hsa-mir-941-4	chr20	62551109	62551180	+	648	130	264	0	371	0	202	78	340	0	miRNA
hsa-mir-941-5	chr20	62551221	62551292	+	648	130	264	0	371	0	202	78	340	0	miRNA
hsa-miR-409-3p	chr14	1.02E+08	1.02E+08	+	614	415	228	2084	3160	740	0	0	193	208	miRNA
hsa-mir-409	chr14	1.02E+08	1.02E+08	+	614	415	228	2084	3160	740	0	0	193	208	miRNA
hsa-mir-1307	chr10	1.05E+08	1.05E+08	-	600	207	1046	370	554	421	284	220	454	521	miRNA
hsa-miR-130b-3p	chr22	22007643	22007664	+	599	418	8712	351	740	219	1286	286	1620	1138	miRNA
hsa-miR-223-5p	chrX	65238737	65238758	+	595	330	611	506	2656	220	49	0	71	1277	miRNA
hsa-miR-378c	chr10	1.33E+08	1.33E+08	-	579	301	1846	238	572	87	700	66	1582	606	miRNA
hsa-mir-378c	chr10	1.33E+08	1.33E+08	-	579	301	1846	238	572	87	700	66	1582	606	miRNA
hsa-mir-23a	chr19	13947401	13947473	-	575	541	3601	398	2202	338	325	1	206	810	miRNA
hsa-miR-23a-3p	chr19	13947409	13947429	-	562	541	3596	370	2193	338	325	1	206	810	miRNA
hsa-miR-4446-3p	chr3	1.13E+08	1.13E+08	+	550	123	35	60	418	289	49	34	63	181	miRNA
hsa-mir-4446	chr3	1.13E+08	1.13E+08	+	550	123	35	60	418	289	49	34	63	181	miRNA
hsa-miR-1307-3p	chr10	1.05E+08	1.05E+08	-	545	186	731	311	445	355	208	189	320	324	miRNA
hsa-miR-15b-5p	chr3	1.6E+08	1.6E+08	+	544	532	4039	437	1423	160	956	0	3027	891	miRNA
hsa-miR-19a-3p	chr13	92003193	92003215	+	544	1716	5707	866	1312	558	2509	272	5289	3705	miRNA
hsa-mir-15b	chr3	1.6E+08	1.6E+08	+	544	597	4467	548	1541	200	1120	0	3393	891	miRNA
hsa-mir-19a	chr13	92003145	92003226	+	544	1716	5707	866	1312	558	2509	272	5289	3706	miRNA
hsa-miR-10b-5p	chr2	1.77E+08	1.77E+08	+	537	1952	14590	1188	885	1544	3444	300	817	1123	miRNA

hsa-mir-10b	chr2	1.77E+08	1.77E+08	+	537	1952	14622	1188	885	1544	3444	300	817	1123	miRNA
hsa-miR-181b-5p	chr1	1.99E+08	1.99E+08	-	520	677	2087	484	1293	788	596	175	1173	1456	miRNA
hsa-mir-181b-2	chr9	1.27E+08	1.27E+08	+	520	677	2088	484	1293	788	596	175	1173	1456	miRNA
hsa-miR-425-5p	chr3	49057632	49057654	-	518	297	2623	184	824	327	1322	160	1590	1228	miRNA
hsa-miR-328-3p	chr16	67236230	67236251	-	505	135	52	228	328	38	76	31	122	435	miRNA
hsa-mir-328	chr16	67236224	67236298	-	505	135	52	228	328	38	76	31	122	435	miRNA
hsa-miR-126-3p	chr9	1.4E+08	1.4E+08	+	501	1323	1017	702	4514	1604	992	45	807	1232	miRNA
hsa-miR-222-3p	chrX	45606442	45606462	-	494	1177	1251	190	2830	1079	269	184	496	1768	miRNA
hsa-miR-222	chrX	45606421	45606530	-	494	1177	1251	190	2830	1079	269	184	496	1768	miRNA
hsa-mir-345	chr14	1.01E+08	1.01E+08	+	493	84	782	2	612	245	256	42	313	436	miRNA
hsa-miR-142-3p	chr17	56408606	56408628	-	491	373	803	432	1302	750	474	293	720	1897	miRNA
hsa-miR-345-5p	chr14	1.01E+08	1.01E+08	+	487	78	763	0	597	244	252	42	300	428	miRNA
hsa-mir-148b	chr12	54731000	54731098	+	467	725	3131	227	1389	611	981	386	2257	1700	miRNA
hsa-mir-181b-1	chr1	1.99E+08	1.99E+08	-	466	660	2060	484	1213	565	596	174	1172	1385	miRNA
hsa-miR-1246	chr2	1.77E+08	1.77E+08	-	440	779	391	1601	644	1019	476	1165	201	1080	miRNA
hsa-mir-1246	chr2	1.77E+08	1.77E+08	-	440	779	391	1601	644	1019	476	1165	201	1080	miRNA
hsa-miR-629-5p	chr15	70371766	70371786	-	428	225	4494	351	705	180	888	24	2196	979	miRNA
hsa-mir-629	chr15	70371711	70371807	-	428	225	4511	351	705	180	888	24	2196	979	miRNA
hsa-miR-148b-3p	chr12	54731062	54731083	+	425	696	3086	227	1320	611	981	271	2257	1700	miRNA
hsa-miR-10a-5p	chr17	46657266	46657288	-	423	577	3039	133	191	330	278	172	95	426	miRNA
hsa-mir-10a	chr17	46657200	46657309	-	423	577	3159	133	203	330	334	172	95	426	miRNA
hsa-mir-7-1	chr9	86584663	86584772	-	420	430	1593	270	843	178	975	0	1174	1578	miRNA
hsa-miR-484	chr16	15737158	15737179	+	414	292	1913	136	846	210	739	48	1803	810	miRNA
hsa-mir-484	chr16	15737151	15737229	+	414	292	1913	136	846	210	739	48	1803	810	miRNA
hsa-miR-182-5p	chr7	1.29E+08	1.29E+08	-	412	1111	2649	772	809	412	1097	339	1164	1493	miRNA
hsa-mir-182	chr7	1.29E+08	1.29E+08	-	412	1130	2649	772	821	412	1097	339	1164	1493	miRNA
hsa-miR-548j-5p	chr22	26951240	26951261	-	411	26	114	0	106	43	0	0	30	0	miRNA
hsa-mir-548j	chr22	26951178	26951289	-	411	26	114	0	106	44	0	0	30	0	miRNA
hsa-miR-29a-3p	chr7	1.31E+08	1.31E+08	-	398	123	1102	127	475	83	214	44	109	374	miRNA
hsa-mir-29a	chr7	1.31E+08	1.31E+08	-	398	123	1102	127	475	83	214	44	109	374	miRNA
hsa-miR-127-3p	chr14	1.01E+08	1.01E+08	+	389	30	0	179	104	93	0	0	11	9	miRNA
hsa-mir-127	chr14	1.01E+08	1.01E+08	+	389	44	0	246	129	93	0	39	11	57	miRNA
hsa-miR-542-3p	chrX	1.34E+08	1.34E+08	-	384	147	154	114	106	60	0	0	48	182	miRNA
hsa-mir-542	chrX	1.34E+08	1.34E+08	-	384	147	188	114	112	60	0	0	48	182	miRNA
hsa-mir-361	chrX	85158641	85158712	-	382	379	864	196	901	137	308	0	484	452	miRNA
hsa-miR-382-5p	chr14	1.02E+08	1.02E+08	+	377	353	517	2250	2599	535	162	56	201	303	miRNA
hsa-mir-382	chr14	1.02E+08	1.02E+08	+	377	373	551	2255	2705	535	205	56	217	303	miRNA
hsa-miR-660-5p	chrX	49777864	49777885	+	363	498	6724	652	687	521	1015	74	3162	1171	miRNA
hsa-mir-660	chrX	49777849	49777945	+	363	498	6724	652	694	521	1071	74	3186	1171	miRNA
hsa-mir-532	chrX	49767754	49767844	+	362	595	3402	511	781	146	1004	0	2337	1332	miRNA
hsa-miR-152-3p	chr17	46114540	46114560	-	357	233	384	337	990	291	140	117	127	582	miRNA
hsa-mir-152	chr17	46114527	46114613	-	357	233	384	337	990	291	140	117	127	583	miRNA
hsa-miR-378i	chr22	42319275	42319295	-	349	30	360	148	196	86	91	3	373	347	miRNA
hsa-mir-378i	chr22	42319226	42319301	-	349	30	360	148	196	86	91	3	373	347	miRNA
hsa-miR-192-5p	chr11	64658675	64658695	-	338	502	2938	627	2682	1019	2102	153	1625	1508	miRNA
hsa-mir-192	chr11	64658609	64658718	-	338	502	2938	627	2682	1019	2102	153	1625	1508	miRNA
hsa-miR-301a-3p	chr17	57228510	57228532	-	329	359	152	176	625	256	67	0	47	321	miRNA
hsa-miR-421	chrX	73438227	73438249	-	329	309	2762	220	767	176	854	39	1376	1093	miRNA
hsa-mir-301a	chr17	57228497	57228582	-	329	359	152	176	625	256	67	0	47	321	miRNA
hsa-mir-421	chrX	73438212	73438296	-	329	309	2762	220	767	176	854	39	1376	1093	miRNA
hsa-miR-449c-5p	chr5	54468141	54468165	-	326	190	90	72	92	208	92	0	102	149	miRNA
hsa-mir-449c	chr5	54468090	54468181	-	326	190	90	72	93	208	92	0	102	149	miRNA
hsa-miR-7-5p	chr15	89155087	89155110	+	325	411	1542	270	764	178	905	0	1152	1578	miRNA
hsa-mir-7-2	chr15	89155056	89155165	+	325	409	1552	270	747	168	905	0	1153	1578	miRNA
hsa-mir-7-3	chr19	4770682	4770791	+	325	407	1541	270	746	168	905	0	1152	1578	miRNA
hsa-mir-941-1	chr20	62550802	62550873	+	325	65	132	0	206	0	101	39	170	39	miRNA
hsa-miR-941	chr20	62550848	62550870	+	324	65	132	0	186	0	101	39	170	0	miRNA
hsa-miR-181d-5p	chr19	13985724	13985746	+	319	96	57	76	256	64	33	0	0	233	miRNA
hsa-mir-181d	chr19	13985689	13985825	+	319	96	57	76	256	64	33	0	0	233	miRNA
hsa-miR-342-5p	chr14	1.01E+08	1.01E+08	+	294	54	219	20	206	161	141	0	77	214	miRNA
hsa-mir-342	chr14	1.01E+08	1.01E+08	+	294	54	250	134	232	201	223	0	95	214	miRNA
hsa-miR-10395-5p	chr19	12814461	12814478	-	290	58	0	0	0	0	0	121	61	225	miRNA
hsa-mir-10395	chr19	12814415	12814478	-	290	58	0	0	3	0	0	121	61	225	miRNA
hsa-miR-769-5p	chr19	46522219	46522240	+	289	94	173	109	80	107	146	33	53	202	miRNA
hsa-mir-769	chr19	46522190	46522307	+	289	115	173	109	80	107	146	33	84	202	miRNA
hsa-mir-654	chr14	1.02E+08	1.02E+08	+	280	23	39	299	376	110	0	81	34	45	miRNA
hsa-miR-532-5p	chrX	49767773	49767794	+	270	541	2785	484	729	111	745	0	2079	1144	miRNA
hsa-mir-576	chr4	1.1E+08	1.1E+08	+	262	161	574	40	263	0	292	0	75	175	miRNA
hsa-miR-140-5p	chr16	69967006	69967027	+	260	184	703	143	701	294	324	20	155	904	miRNA
hsa-mir-324	chr17	7126616	7126698	-	260	106	1190	257	305	20	567	7	713	708	miRNA
hsa-miR-324-5p	chr17	7126662	7126683	-	257	17	489	199	155	2	239	7	258	269	miRNA
hsa-miR-375-3p	chr2	2.2E+08	2.2E+08	-	256	102	947	0	448	133	85	0	39	92	miRNA
hsa-miR-584-5p	chr5	1.48E+08	1.48E+08	-	256	419	2413	389	860	556	352	90	482	462	miRNA
hsa-mir-375	chr2	2.2E+08	2.2E+08	-	256	102	947	0	448	133	85	0	39	92	miRNA
hsa-mir-584	chr5	1.48E+08	1.48E+08	-	256	419	2413	389	860	556	352	90	482	462	miRNA
hsa-miR-1908-5p	chr11	61582681	61582701	-	252	19	221	0	284	122	101	0	266	79	miRNA
hsa-mir-1908	chr11	61582633	61582712	-	252	19	221	0	284	122	101	0	266	79	miRNA
hsa-mir-374a	chrX	73507121	73507192	-	250	92	89	0	98	74	47	0	20	140	miRNA
hsa-mir-128-1	chr2	1.36E+08	1.36E+08	+	246	580	5156	320	1575	575	1746	52	2724	1323	miRNA
hsa-mir-125a	chr19	52196507	52196592	+	232	71	85	26	91	42	79	0	27	84	miRNA
hsa-mir-625	chr14	65937820	65937904	+	232	448	121	10	593	127	196	0	153	173	miRNA
hsa-mir-454	chr17	57215119	57215233	-	222	187	236	131	959	92	187	0	254	438	miRNA
hsa-mir-4732	chr17	27188673	27188748	-	212	173	3017	50	209	364	1495	0	2753	1084	miRNA

hsa-miR-10395-3p	chr19	12814415	12814435	-	200	48	0	0	3	0	0	116	54	168	miRNA
hsa-miR-654-5p	chr14	1.02E+08	1.02E+08	+	198	7	18	165	115	0	0	81	4	45	miRNA
hsa-miR-181c-5p	chr19	13985539	13985560	+	197	115	297	89	235	158	205	1	83	203	miRNA
hsa-mir-181c	chr19	13985513	13985622	+	197	138	366	145	290	205	205	1	83	295	miRNA
hsa-miR-148a-5p	chr7	25989580	25989601	-	194	50	57	0	196	16	0	0	0	120	miRNA
hsa-mir-183	chr7	1.29E+08	1.29E+08	-	194	19	71	0	6	0	170	0	66	46	miRNA
hsa-miR-361-3p	chrX	85158646	85158668	-	192	187	417	81	597	129	180	0	249	364	miRNA
hsa-miR-425-3p	chr3	49057592	49057613	-	191	242	987	285	550	184	641	89	851	311	miRNA
hsa-miR-10527-5p	chr12	64217443	64217465	+	190	43	281	0	110	63	81	0	390	197	miRNA
hsa-miR-361-5p	chrX	85158686	85158707	-	190	192	447	115	304	8	128	0	235	88	miRNA
hsa-mir-10527	chr12	64217443	64217507	+	190	43	281	0	110	63	81	0	390	197	miRNA
hsa-miR-454-3p	chr17	57215148	57215170	-	189	146	157	131	872	92	155	0	116	425	miRNA
hsa-miR-370-3p	chr14	1.01E+08	1.01E+08	+	188	0	0	0	47	0	0	0	0	0	miRNA
hsa-mir-370	chr14	1.01E+08	1.01E+08	+	188	0	0	0	91	0	0	0	0	0	miRNA
hsa-mir-339	chr7	1062569	1062662	-	184	185	770	132	301	75	258	0	175	554	miRNA
hsa-miR-203a-3p	chr14	1.05E+08	1.05E+08	+	182	68	16	183	167	106	112	0	38	265	miRNA
hsa-mir-203a	chr14	1.05E+08	1.05E+08	+	182	68	16	183	188	106	112	0	58	265	miRNA
hsa-miR-128-3p	chr2	1.36E+08	1.36E+08	+	181	519	5121	319	1325	500	1714	52	2684	1184	miRNA
hsa-miR-339-5p	chr7	1062626	1062648	-	181	98	448	132	211	75	1	0	110	401	miRNA
hsa-mir-128-2	chr3	35785968	35786051	+	181	519	5121	319	1325	500	1714	52	2684	1184	miRNA
hsa-miR-424-3p	chrX	1.34E+08	1.34E+08	-	176	111	743	30	412	189	171	0	366	201	miRNA
hsa-miR-576-3p	chr4	1.1E+08	1.1E+08	+	176	161	488	40	222	0	142	0	75	132	miRNA
hsa-mir-424	chrX	1.34E+08	1.34E+08	-	176	138	1275	85	696	220	205	0	388	376	miRNA
hsa-miR-4433b-3p	chr2	64567903	64567923	-	172	273	124	84	462	107	80	0	152	156	miRNA
hsa-mir-4433b	chr2	64567881	64567982	-	172	395	124	116	531	133	80	0	183	186	miRNA
hsa-miR-374a-5p	chrX	73507160	73507181	-	168	15	0	0	48	0	47	0	0	0	miRNA
hsa-miR-410-3p	chr14	1.02E+08	1.02E+08	+	168	69	66	80	237	60	187	0	51	58	miRNA
hsa-mir-410	chr14	1.02E+08	1.02E+08	+	168	69	66	80	237	60	187	0	51	58	miRNA
hsa-miR-5010-5p	chr17	40666226	40666247	+	167	41	788	41	67	97	473	0	631	130	miRNA
hsa-mir-5010	chr17	40666206	40666325	+	167	41	788	41	67	97	473	0	631	130	miRNA
hsa-miR-1843	chr1	1.76E+08	1.76E+08	-	166	146	226	292	345	153	217	0	203	397	miRNA
hsa-miR-877-5p	chr6	30552109	30552128	+	166	0	28	0	53	45	0	0	35	0	miRNA
hsa-mir-1843	chr1	1.76E+08	1.76E+08	-	166	156	226	334	378	153	217	0	203	397	miRNA
hsa-mir-877	chr6	30552109	30552194	+	166	33	28	0	55	45	0	0	35	0	miRNA
hsa-miR-652-3p	chrX	1.09E+08	1.09E+08	+	163	200	1751	113	673	36	435	49	1109	596	miRNA
hsa-mir-652	chrX	1.09E+08	1.09E+08	+	163	200	1751	113	673	60	435	49	1109	596	miRNA
hsa-miR-125a-3p	chr19	52196559	52196580	+	160	28	85	26	45	0	79	0	27	0	miRNA
hsa-miR-3168	chr13	41675212	41675228	-	159	82	127	550	55	177	175	349	151	276	miRNA
hsa-mir-3168	chr13	41675155	41675236	-	159	82	127	550	55	178	175	349	151	317	miRNA
hsa-mir-3976	chr18	5840694	5840832	+	159	19	91	210	161	177	122	0	43	313	miRNA
hsa-miR-33b-5p	chr17	17717211	17717230	-	158	18	986	160	60	66	351	41	485	114	miRNA
hsa-mir-33b	chr17	17717150	17717245	-	158	33	986	160	76	66	351	41	485	114	miRNA
hsa-mir-1273h	chr16	24214437	24214552	+	155	43	32	0	242	87	34	0	20	226	miRNA
hsa-miR-625-3p	chr14	65937871	65937892	+	154	366	0	10	308	79	62	0	90	89	miRNA
hsa-miR-25-5p	chr7	99691233	99691253	-	152	0	368	0	72	53	178	0	489	81	miRNA
hsa-miR-483-5p	chr11	2155411	2155432	-	152	27	5747	159	684	372	967	93	167	202	miRNA
hsa-mir-483	chr11	2155364	2155439	-	152	27	5747	159	694	372	967	93	169	202	miRNA
hsa-miR-433-3p	chr14	1.01E+08	1.01E+08	+	148	75	15	120	156	0	0	0	0	0	miRNA
hsa-mir-433	chr14	1.01E+08	1.01E+08	+	148	75	15	120	162	0	0	0	0	0	miRNA
hsa-miR-431-5p	chr14	1.01E+08	1.01E+08	+	143	88	28	176	435	147	0	0	43	0	miRNA
hsa-miR-671-3p	chr7	1.51E+08	1.51E+08	+	143	61	28	0	70	102	63	0	19	29	miRNA
hsa-mir-431	chr14	1.01E+08	1.01E+08	+	143	88	28	176	435	147	0	0	43	0	miRNA
hsa-mir-671	chr7	1.51E+08	1.51E+08	+	143	61	51	0	92	102	63	0	19	42	miRNA
hsa-miR-4732-3p	chr17	27188686	27188706	-	141	35	799	50	128	2	288	0	1114	438	miRNA
hsa-miR-1291	chr12	49048277	49048300	-	134	0	0	0	0	0	0	0	21	0	miRNA
hsa-mir-1291	chr12	49048227	49048313	-	134	0	0	0	0	0	0	0	21	0	miRNA
hsa-miR-20b-5p	chrX	1.33E+08	1.33E+08	-	126	356	1480	108	719	45	813	0	2359	1750	miRNA
hsa-miR-29c-3p	chr1	2.08E+08	2.08E+08	-	126	101	1242	199	128	22	191	0	596	154	miRNA
hsa-mir-20b	chrX	1.33E+08	1.33E+08	-	126	356	1480	108	719	45	813	0	2377	1772	miRNA
hsa-mir-29c	chr1	2.08E+08	2.08E+08	-	126	101	1320	199	148	22	232	0	596	154	miRNA
hsa-miR-1273h-3p	chr16	24214507	24214528	+	117	0	0	0	146	0	0	0	20	80	miRNA
hsa-mir-4258	chr1	1.55E+08	1.55E+08	+	111	75	36	112	26	0	33	0	12	39	miRNA
hsa-miR-4306	chr13	1E+08	1E+08	+	110	17	600	0	48	25	449	0	354	75	miRNA
hsa-mir-4306	chr13	1E+08	1E+08	+	110	17	600	0	48	25	449	0	354	75	miRNA
hsa-miR-493-5p	chr14	1.01E+08	1.01E+08	+	107	252	60	576	1479	232	0	86	68	40	miRNA
hsa-mir-493	chr14	1.01E+08	1.01E+08	+	107	255	60	685	1544	232	0	86	76	40	miRNA
hsa-miR-183-3p	chr7	1.29E+08	1.29E+08	-	103	0	23	0	0	0	0	0	0	0	miRNA
hsa-miR-30e-3p	chr1	41220085	41220106	+	100	163	204	115	506	7	109	216	139	347	miRNA
hsa-miR-766-5p	chrX	1.19E+08	1.19E+08	-	97	53	69	66	98	59	0	0	91	83	miRNA
hsa-mir-766	chrX	1.19E+08	1.19E+08	-	97	53	69	66	118	59	0	0	108	83	miRNA
hsa-miR-193a-5p	chr17	29887035	29887056	+	95	38	173	0	204	31	84	0	34	73	miRNA
hsa-miR-7-1-3p	chr9	86584686	86584707	-	95	19	51	0	79	0	70	0	22	0	miRNA
hsa-mir-193a	chr17	29887015	29887102	+	95	38	173	0	204	31	84	0	34	73	miRNA
hsa-miR-3138	chr4	10080246	10080269	-	94	19	349	0	119	145	155	0	67	19	miRNA
hsa-mir-3138	chr4	10080235	10080316	-	94	19	349	0	119	145	155	0	67	19	miRNA
hsa-miR-323a-3p	chr14	1.01E+08	1.01E+08	+	93	55	111	177	371	105	54	0	17	44	miRNA
hsa-miR-377-5p	chr14	1.02E+08	1.02E+08	+	93	0	0	0	70	14	0	0	0	0	miRNA
hsa-mir-323a	chr14	1.01E+08	1.01E+08	+	93	55	111	177	375	105	55	0	17	44	miRNA
hsa-mir-377	chr14	1.02E+08	1.02E+08	+	93	0	0	0	70	14	0	0	0	0	miRNA
hsa-miR-191-3p	chr3	49058064	49058085	-	92	17	24	122	14	0	0	0	0	36	miRNA
hsa-miR-532-3p	chrX	49767810	49767831	+	92	54	617	27	52	35	259	0	258	188	miRNA
hsa-miR-9-5p	chr1	1.56E+08	1.56E+08	-	92	62	0	0	59	0	0	0	0	51	miRNA
hsa-mir-9-1	chr1	1.56E+08	1.56E+08	-	92	62	0	0	59	0	0	0	0	51	miRNA

hsa-mir-9-2	chr5	87962671	87962757	-	92	62	0	0	59	0	0	0	0	51	miRNA
hsa-mir-9-3	chr15	89911248	89911337	+	92	62	0	0	59	0	0	0	0	51	miRNA
hsa-miR-183-5p	chr7	1.29E+08	1.29E+08	-	91	19	48	0	6	0	170	0	66	46	miRNA
hsa-miR-1468-5p	chrX	63005935	63005955	-	89	1	27	0	26	0	0	0	38	0	miRNA
hsa-mir-1468	chrX	63005882	63005967	-	89	1	27	0	27	0	0	0	38	0	miRNA
hsa-miR-1278	chr1	1.93E+08	1.93E+08	+	88	0	43	0	8	0	0	0	5	0	miRNA
hsa-mir-1278	chr1	1.93E+08	1.93E+08	+	88	0	43	0	8	0	0	0	5	0	miRNA
hsa-miR-1976	chr1	26881065	26881084	+	87	0	45	0	10	0	19	0	46	145	miRNA
hsa-miR-335-5p	chr7	1.3E+08	1.3E+08	+	87	152	458	0	334	199	194	48	174	95	miRNA
hsa-mir-1976	chr1	26881033	26881084	+	87	0	168	0	10	24	19	0	122	145	miRNA
hsa-mir-335	chr7	1.3E+08	1.3E+08	+	87	160	492	19	346	237	194	48	174	146	miRNA
hsa-miR-1255b-5p	chr1	1.68E+08	1.68E+08	+	86	69	500	1	56	40	272	0	224	200	miRNA
hsa-miR-150-3p	chr19	50004054	50004075	-	86	42	37	57	48	0	0	0	35	184	miRNA
hsa-miR-296-3p	chr20	57392681	57392702	-	86	0	49	0	7	0	0	0	23	0	miRNA
hsa-miR-3605-5p	chr1	33798050	33798072	-	86	0	248	0	94	1	107	0	225	21	miRNA
hsa-miR-576-5p	chr4	1.1E+08	1.1E+08	+	86	0	86	0	41	0	150	0	0	43	miRNA
hsa-miR-6740-5p	chr1	2.02E+08	2.02E+08	+	86	0	156	0	8	0	0	0	0	0	miRNA
hsa-mir-1255b-1	chr4	36427988	36428050	-	86	69	500	1	56	40	272	0	224	200	miRNA
hsa-mir-1255b-2	chr1	1.68E+08	1.68E+08	+	86	68	501	0	57	40	274	0	224	201	miRNA
hsa-mir-150	chr19	50004042	50004125	-	86	69	148	110	167	59	0	0	72	184	miRNA
hsa-mir-296	chr20	57392670	57392749	-	86	0	50	0	9	0	0	0	43	0	miRNA
hsa-mir-3605	chr1	33797994	33798093	-	86	22	282	0	125	1	144	0	249	106	miRNA
hsa-mir-548ae-2	chr5	57825870	57825936	-	86	40	747	63	83	129	317	0	559	299	miRNA
hsa-mir-6740	chr1	2.02E+08	2.02E+08	+	86	0	156	0	8	0	0	0	0	0	miRNA
hsa-miR-190b-5p	chr1	1.54E+08	1.54E+08	-	85	0	66	64	69	0	35	0	43	160	miRNA
hsa-miR-548ae-5p	chr5	57825912	57825931	-	85	40	747	63	83	129	317	0	559	299	miRNA
hsa-mir-190b	chr1	1.54E+08	1.54E+08	-	85	0	66	64	69	0	35	0	43	160	miRNA
hsa-miR-24-2-5p	chr19	13947140	13947161	-	84	102	39	0	163	0	0	0	0	162	miRNA
hsa-miR-6852-5p	chr9	35710713	35710733	-	83	62	42	0	179	56	0	0	107	39	miRNA
hsa-mir-6852	chr9	35710673	35710738	-	83	62	42	0	193	56	0	0	130	40	miRNA
hsa-miR-1270	chr19	20510129	20510151	-	82	0	720	209	72	5	244	0	229	1	miRNA
hsa-miR-374a-3p	chrX	73507130	73507151	-	82	77	89	0	50	74	0	0	20	140	miRNA
hsa-miR-654-3p	chr14	1.02E+08	1.02E+08	+	82	16	21	134	261	110	0	0	30	0	miRNA
hsa-mir-1270	chr19	20510081	20510163	-	82	0	720	209	72	5	244	0	229	1	miRNA
hsa-miR-210-3p	chr11	568112	568133	-	81	135	1503	97	211	180	304	56	837	554	miRNA
hsa-mir-210	chr11	568089	568198	-	81	135	1503	97	211	180	304	56	837	554	miRNA
hsa-mir-7703	chr14	24612698	24612774	-	81	9	28	30	2	124	20	41	0	1	miRNA
hsa-miR-130b-5p	chr22	22007605	22007625	+	80	85	45	116	200	146	35	0	27	0	miRNA
hsa-miR-212-3p	chr17	1953584	1953604	-	80	0	0	0	21	0	0	0	0	0	miRNA
hsa-mir-212	chr17	1953565	1953674	-	80	0	0	0	21	28	0	0	0	0	miRNA
hsa-miR-338-3p	chr17	79099687	79099708	-	79	0	39	0	48	0	0	0	0	60	miRNA
hsa-miR-6503-5p	chr11	59976591	59976613	-	79	0	0	0	12	0	0	0	0	0	miRNA
hsa-mir-338	chr17	79099683	79099749	-	79	0	39	0	85	0	0	0	0	60	miRNA
hsa-mir-6503	chr11	59976544	59976629	-	79	0	27	0	20	0	0	48	0	0	miRNA
hsa-miR-204-3p	chr9	73424909	73424929	-	78	0	89	0	24	33	0	0	20	0	miRNA
hsa-miR-3617-5p	chr20	44333789	44333810	-	78	6	21	0	29	0	0	0	0	39	miRNA
hsa-miR-625-5p	chr14	65937834	65937854	+	78	82	121	0	285	48	134	0	63	84	miRNA
hsa-mir-204	chr9	73424891	73425000	-	78	19	89	0	36	33	0	0	20	0	miRNA
hsa-mir-3617	chr20	44333741	44333819	-	78	6	21	0	29	0	0	0	0	39	miRNA
hsa-miR-3145-5p	chr6	1.39E+08	1.39E+08	-	77	0	0	0	0	0	0	0	0	0	miRNA
hsa-miR-376b-3p	chr14	1.02E+08	1.02E+08	+	77	0	0	0	0	0	0	0	0	0	miRNA
hsa-mir-3145	chr6	1.39E+08	1.39E+08	-	77	0	0	0	0	0	0	0	0	0	miRNA
hsa-mir-376b	chr14	1.02E+08	1.02E+08	+	77	0	0	0	1	0	0	0	0	0	miRNA
hsa-miR-1287-5p	chr10	1E+08	1E+08	-	76	23	169	123	100	0	1	0	81	327	miRNA
hsa-miR-2115-3p	chr3	48357871	48357892	-	76	50	31	0	67	0	0	0	0	61	miRNA
hsa-mir-1287	chr10	1E+08	1E+08	-	76	23	169	123	100	0	1	0	81	327	miRNA
hsa-mir-2115	chr3	48357850	48357949	-	76	50	31	0	67	0	0	0	0	61	miRNA
hsa-miR-4511	chr15	66011635	66011656	-	75	0	50	0	0	18	0	0	31	76	miRNA
hsa-mir-4511	chr15	66011584	66011670	-	75	0	50	0	0	18	0	0	36	76	miRNA
hsa-miR-181a-3p	chr1	1.99E+08	1.99E+08	-	73	76	89	34	99	28	43	0	23	57	miRNA
hsa-miR-27a-5p	chr19	13947301	13947322	-	73	0	0	0	45	0	0	0	10	39	miRNA
hsa-miR-29b-2-5p	chr1	2.08E+08	2.08E+08	-	73	0	125	0	26	0	102	0	75	80	miRNA
hsa-miR-6877-5p	chr9	1.36E+08	1.36E+08	+	73	59	73	0	54	0	99	0	107	0	miRNA
hsa-mir-29b-2	chr1	2.08E+08	2.08E+08	-	73	0	146	2	27	0	142	0	92	80	miRNA
hsa-mir-6877	chr9	1.36E+08	1.36E+08	+	73	59	73	0	54	0	99	0	107	0	miRNA
hsa-miR-125a-5p	chr19	52196521	52196544	+	72	43	0	0	46	42	0	0	0	84	miRNA
hsa-mir-378d-1	chr4	5925002	5925055	-	72	62	208	23	148	16	181	12	316	142	miRNA
hsa-miR-4732-5p	chr17	27188718	27188740	-	71	138	2218	0	81	362	1207	0	1639	646	miRNA
hsa-miR-378d	chr4	5925006	5925025	-	70	56	208	23	127	15	180	13	244	141	miRNA
hsa-mir-378d-2	chr8	94928250	94928347	-	70	56	208	23	127	15	180	13	244	141	miRNA
hsa-miR-760	chr1	94312436	94312455	+	69	66	155	67	103	0	63	0	44	42	miRNA
hsa-mir-760	chr1	94312388	94312467	+	69	66	155	67	103	0	63	0	44	42	miRNA
hsa-miR-3909	chr22	35731703	35731724	+	67	23	0	0	20	0	0	0	17	0	miRNA
hsa-mir-3909	chr22	35731633	35731751	+	67	23	0	0	20	0	0	0	17	0	miRNA
hsa-miR-6722-3p	chr9	1.4E+08	1.4E+08	-	66	0	0	0	0	0	0	0	0	0	miRNA
hsa-mir-6722	chr9	1.4E+08	1.4E+08	-	66	0	0	0	0	0	0	0	0	0	miRNA
hsa-miR-181a-2-3p	chr9	1.27E+08	1.27E+08	+	65	132	87	85	209	0	108	0	78	159	miRNA
hsa-miR-1260a	chr14	77732574	77732591	+	64	7	0	0	114	2	0	0	0	77	miRNA
hsa-miR-6741-5p	chr1	2.26E+08	2.26E+08	-	64	0	25	0	42	22	0	0	13	0	miRNA
hsa-mir-1260a	chr14	77732561	77732633	+	64	7	0	0	114	2	0	0	0	77	miRNA
hsa-mir-6741	chr1	2.26E+08	2.26E+08	-	64	9	25	0	47	22	39	0	13	46	miRNA
hsa-miR-1294	chr5	1.54E+08	1.54E+08	+	61	62	945	0	112	0	257	0	336	111	miRNA
hsa-miR-543	chr14	1.01E+08	1.01E+08	+	61	41	34	199	348	36	1	0	50	52	miRNA

hsa-mir-1294	chr5	1.54E+08	1.54E+08	+	61	62	945	0	112	1	258	0	336	111	miRNA
hsa-mir-543	chr14	1.01E+08	1.01E+08	+	61	41	34	199	348	36	1	0	50	52	miRNA
hsa-miR-4286	chr8	10524498	10524514	+	59	24	0	0	32	0	0	0	0	29	miRNA
hsa-mir-4286	chr8	10524488	10524580	+	59	24	0	0	32	0	0	0	0	29	miRNA
hsa-miR-548ay-5p	chr3	32547834	32547854	-	58	62	861	63	105	129	361	0	601	272	miRNA
hsa-miR-548d-5p	chr17	65467656	65467677	-	58	62	861	63	105	129	361	0	601	272	miRNA
hsa-mir-548ay	chr3	32547775	32547881	-	58	62	861	63	105	129	361	0	601	273	miRNA
hsa-mir-548d-1	chr8	1.24E+08	1.24E+08	-	58	62	861	63	105	129	361	0	601	272	miRNA
hsa-mir-548d-2	chr17	65467605	65467701	-	58	62	861	63	105	129	361	0	601	272	miRNA
hsa-miR-320e	chr19	47212551	47212568	-	57	68	689	22	641	21	158	1	1453	213	miRNA
hsa-mir-320e	chr19	47212539	47212611	-	57	68	689	22	641	21	158	1	1453	213	miRNA
hsa-miR-1307-5p	chr10	1.05E+08	1.05E+08	-	55	21	315	58	109	66	76	31	134	197	miRNA
hsa-miR-92b-3p	chr1	1.55E+08	1.55E+08	+	54	288	2426	412	465	227	1043	144	2225	657	miRNA
hsa-mir-92b	chr1	1.55E+08	1.55E+08	+	54	304	2502	412	471	228	1099	144	2319	657	miRNA
hsa-miR-6743-5p	chr11	209341	209362	+	53	0	0	0	6	0	0	0	23	0	miRNA
hsa-mir-6743	chr11	209336	209406	+	53	0	0	0	6	0	0	0	23	0	miRNA
hsa-mir-3180-1	chr16	15005077	15005170	+	51	17	12	0	8	1	15	1	12	0	miRNA
hsa-mir-3180-2	chr16	16403736	16403823	+	51	17	12	0	8	1	15	1	12	0	miRNA
hsa-mir-3180-3	chr16	18496035	18496128	-	51	17	12	0	8	1	15	1	12	0	miRNA
hsa-miR-6855-5p	chr9	1.33E+08	1.33E+08	+	50	0	15	0	1	0	9	0	10	10	miRNA
hsa-mir-6855	chr9	1.33E+08	1.33E+08	+	50	0	15	0	1	0	9	0	10	10	miRNA
hsa-mir-6779	chr17	37071232	37071295	+	47	38	66	65	1	85	53	223	27	44	miRNA
hsa-miR-1292-5p	chr20	2633425	2633449	+	46	0	36	0	2	0	8	0	23	0	miRNA
hsa-miR-5187-5p	chr1	1.61E+08	1.61E+08	+	46	13	37	43	30	1	27	0	50	144	miRNA
hsa-mir-1292	chr20	2633423	2633488	+	46	0	36	0	2	0	8	0	23	0	miRNA
hsa-mir-5187	chr1	1.61E+08	1.61E+08	+	46	14	37	43	30	1	27	0	50	144	miRNA
hsa-mir-770	chr14	1.01E+08	1.01E+08	+	46	0	0	0	0	0	0	0	0	0	miRNA
hsa-miR-1-3p	chr18	19408976	19408997	-	44	196	551	49	132	166	23	10	33	48	miRNA
hsa-miR-3180	chr16	2186077	2186095	-	44	0	12	0	8	0	14	0	12	0	miRNA
hsa-miR-3180-3p	chr16	15005138	15005159	+	44	0	12	0	8	1	14	0	12	0	miRNA
hsa-mir-1-1	chr20	61151513	61151583	+	44	196	551	49	132	166	23	10	33	48	miRNA
hsa-mir-1-2	chr18	19408965	19409049	-	44	205	552	49	135	166	23	10	33	54	miRNA
hsa-mir-3180-4	chr16	15248707	15248859	-	44	17	12	0	11	0	14	0	12	0	miRNA
hsa-mir-3180-5	chr16	2185978	2186130	-	44	17	12	0	11	0	14	0	12	0	miRNA
hsa-miR-148b-5p	chr12	54731024	54731045	+	42	29	45	0	69	0	0	115	0	0	miRNA
hsa-miR-4443	chr3	48238062	48238078	+	42	4	23	14	21	1	38	0	46	37	miRNA
hsa-mir-4443	chr3	48238054	48238106	+	42	4	23	14	21	1	38	0	46	37	miRNA
hsa-miR-1273h-5p	chr16	24214469	24214489	+	38	43	32	0	96	87	34	0	0	146	miRNA
hsa-miR-6515-5p	chr19	13051300	13051320	+	36	27	34	0	24	0	0	0	1	115	miRNA
hsa-mir-6515	chr19	13051298	13051354	+	36	27	34	0	24	0	0	0	1	115	miRNA
hsa-miR-34a-5p	chr1	9211794	9211815	-	35	75	428	40	173	137	172	0	50	199	miRNA
hsa-miR-6511a-5p	chr16	15019798	15019820	+	35	17	1	0	4	0	0	0	0	0	miRNA
hsa-miR-6511b-5p	chr16	2156721	2156744	-	35	17	1	0	4	0	0	0	0	0	miRNA
hsa-mir-34a	chr1	9211727	9211836	-	35	75	428	40	175	137	172	0	50	199	miRNA
hsa-mir-6511a-1	chr16	15019794	15019860	+	35	58	36	0	11	0	39	0	35	0	miRNA
hsa-mir-6511a-2	chr16	16418445	16418511	+	35	58	36	0	11	0	39	0	35	0	miRNA
hsa-mir-6511a-3	chr16	16462733	16462799	+	35	58	36	0	11	0	39	0	35	0	miRNA
hsa-mir-6511a-4	chr16	18437870	18437936	-	35	58	36	0	11	0	39	0	35	0	miRNA
hsa-mir-6511b-1	chr16	2156670	2156754	-	35	21	1	0	4	0	1	0	0	0	miRNA
hsa-mir-6511b-2	chr16	15227932	15228002	-	35	21	1	0	4	0	1	0	0	0	miRNA
hsa-miR-454-5p	chr17	57215189	57215210	-	33	41	79	0	87	0	32	0	138	13	miRNA
hsa-miR-6816-5p	chr22	20102249	20102269	-	33	0	0	1	30	8	0	0	2	0	miRNA
hsa-mir-6816	chr22	20102209	20102274	-	33	0	0	1	30	8	0	0	2	0	miRNA
hsa-miR-151b	chr14	1.01E+08	1.01E+08	-	31	75	608	53	116	44	115	1	86	135	miRNA
hsa-miR-6842-5p	chr8	27290892	27290913	+	31	0	4	0	12	0	0	0	0	0	miRNA
hsa-mir-151b	chr14	1.01E+08	1.01E+08	-	31	75	608	53	116	44	115	1	86	135	miRNA
hsa-mir-6842	chr8	27290887	27290951	+	31	7	57	0	18	46	0	0	14	74	miRNA
hsa-miR-206	chr6	52009199	52009220	+	30	4	978	64	20	29	7	0	14	37	miRNA
hsa-mir-206	chr6	52009147	52009232	+	30	4	978	64	20	29	7	0	14	37	miRNA
hsa-miR-30c-5p	chr1	41222972	41222994	+	29	531	402	0	825	98	322	0	329	324	miRNA
hsa-mir-30c-1	chr1	41222956	41223044	+	29	564	448	62	862	98	322	0	338	376	miRNA
hsa-mir-30c-2	chr6	72086663	72086734	-	29	547	402	0	850	98	322	0	329	324	miRNA
hsa-miR-4516	chr16	2183121	2183137	+	26	13	200	1	19	15	91	0	77	2	miRNA
hsa-mir-1284	chr3	71591121	71591240	-	26	0	0	0	0	0	0	0	0	13	miRNA
hsa-mir-4516	chr16	2183120	2183205	+	26	13	200	1	19	15	91	0	77	2	miRNA
hsa-miR-1537-3p	chr1	2.36E+08	2.36E+08	-	25	0	29	0	0	0	0	0	19	0	miRNA
hsa-mir-1537	chr1	2.36E+08	2.36E+08	-	25	0	29	0	0	0	0	0	19	0	miRNA
hsa-miR-378e	chr5	1.69E+08	1.69E+08	+	24	9	61	4	52	7	18	2	102	34	miRNA
hsa-mir-378e	chr5	1.69E+08	1.69E+08	+	24	9	61	4	52	7	18	2	102	34	miRNA
hsa-miR-3691-3p	chr6	5148480	5148501	-	23	0	10	0	0	0	0	0	0	0	miRNA
hsa-mir-3691	chr6	5148467	5148556	-	23	0	10	0	3	0	37	0	0	0	miRNA
hsa-miR-1180-3p	chr17	19247826	19247847	-	21	93	1343	189	178	1	616	0	1371	629	miRNA
hsa-mir-1180	chr17	19247819	19247887	-	21	93	1343	189	178	1	616	0	1371	629	miRNA
hsa-mir-200a	chr1	1103243	1103332	+	20	0	248	0	50	75	68	0	0	30	miRNA
hsa-miR-200a-3p	chr1	1103296	1103317	+	19	0	214	0	50	74	68	0	0	30	miRNA
hsa-mir-4710	chr14	1.05E+08	1.05E+08	-	17	9	20	49	13	45	44	0	8	13	miRNA
hsa-miR-4510	chr15	36219064	36219085	+	16	32	89	16	60	20	42	2	28	72	miRNA
hsa-mir-4510	chr15	36219057	36219124	+	16	32	89	16	60	20	42	2	28	72	miRNA
hsa-miR-4710	chr14	1.05E+08	1.05E+08	-	15	9	20	49	13	45	44	0	8	13	miRNA
hsa-miR-23a-5p	chr19	13947444	13947465	-	13	0	5	28	9	0	0	0	0	0	miRNA
hsa-miR-4429	chr2	11680778	11680797	-	12	11	87	2	29	11	19	0	73	30	miRNA
hsa-miR-4727-3p	chr17	36982124	36982145	+	12	0	0	0	0	0	0	0	0	0	miRNA
hsa-mir-4429	chr2	11680731	11680803	-	12	11	87	2	29	11	19	0	73	30	miRNA

hsa-mir-4727	chr17	36982091	36982145	+	12	0	0	0	0	0	0	0	0	0	miRNA
hsa-miR-379-5p	chr14	1.01E+08	1.01E+08	+	11	125	0	238	551	118	0	0	23	133	miRNA
hsa-mir-379	chr14	1.01E+08	1.01E+08	+	11	125	0	238	565	118	0	0	23	133	miRNA
hsa-miR-28-5p	chr3	1.88E+08	1.88E+08	+	10	224	156	117	848	139	21	48	156	364	miRNA
hsa-miR-548ap-5p	chr15	86368886	86368904	+	10	0	4	0	0	0	0	0	0	0	miRNA
hsa-mir-548ap	chr15	86368866	86368961	+	10	0	4	0	0	0	0	0	0	0	miRNA
hsa-miR-345-3p	chr14	1.01E+08	1.01E+08	+	6	6	19	2	15	1	4	0	13	8	miRNA
hsa-miR-452-5p	chrX	1.51E+08	1.51E+08	-	6	48	184	0	29	0	0	34	41	0	miRNA
hsa-mir-452	chrX	1.51E+08	1.51E+08	-	6	78	190	0	29	0	0	34	41	0	miRNA
hsa-miR-6876-3p	chr8	25202967	25202990	+	5	0	0	0	0	0	0	0	0	0	miRNA
hsa-mir-4741	chr18	20513312	20513401	+	5	12	0	0	7	0	0	0	0	0	miRNA
hsa-mir-6876	chr8	25202918	25202990	+	5	0	0	0	13	0	0	0	0	0	miRNA
hsa-miR-1827	chr12	1.01E+08	1.01E+08	+	4	8	14	2	8	4	4	0	2	9	miRNA
hsa-miR-6073	chr11	15991101	15991120	-	4	1	1	0	0	2	2	0	0	2	miRNA
hsa-mir-1827	chr12	1.01E+08	1.01E+08	+	4	8	14	2	8	4	4	0	2	9	miRNA
hsa-mir-6073	chr11	15991079	15991167	-	4	1	1	0	0	2	2	0	0	2	miRNA
hsa-mir-6507	chr10	1.01E+08	1.01E+08	-	4	0	1	1	0	1	3	0	0	0	miRNA
hsa-miR-1290	chr1	19223572	19223590	-	3	2	2	13	2	9	79	22	2	16	miRNA
hsa-miR-324-3p	chr17	7126627	7126649	-	3	89	701	58	150	18	328	0	455	439	miRNA
hsa-miR-339-3p	chr7	1062591	1062613	-	3	86	322	0	90	0	257	0	65	153	miRNA
hsa-miR-4291	chr9	96581649	96581664	+	3	0	0	0	0	0	0	0	0	0	miRNA
hsa-miR-4485-3p	chr11	10529824	10529843	-	3	5	4	0	12	3	1	1	6	6	miRNA
hsa-miR-548ad-5p	chr2	35696486	35696505	+	3	40	789	63	73	129	316	0	558	287	miRNA
hsa-miR-6782-5p	chr17	42285178	42285202	-	3	0	1	1	1	5	5	0	4	2	miRNA
hsa-mir-1290	chr1	19223565	19223642	-	3	2	2	13	2	9	79	22	2	20	miRNA
hsa-mir-4291	chr9	96581639	96581703	+	3	0	0	0	0	0	0	0	0	0	miRNA
hsa-mir-4485	chr11	10529817	10529873	-	3	9	4	0	12	3	1	1	6	6	miRNA
hsa-mir-5091	chr4	13629489	13629581	+	3	0	0	0	2	0	0	0	20	0	miRNA
hsa-mir-548ad	chr2	35696471	35696552	+	3	40	789	63	73	129	316	0	558	287	miRNA
hsa-mir-5687	chr5	54804678	54804754	-	3	1	0	0	0	0	0	0	0	0	miRNA
hsa-mir-6782	chr17	42285139	42285207	-	3	0	2	1	1	5	5	1	4	2	miRNA
hsa-miR-23b-3p	chr9	97847547	97847569	+	2	80	190	1	418	35	31	1	15	43	miRNA
hsa-miR-23c	chrX	20035219	20035240	-	2	0	3	1	4	0	0	0	0	0	miRNA
hsa-miR-30a-3p	chr6	72113257	72113278	-	2	57	129	1	126	42	16	70	23	67	miRNA
hsa-miR-378h	chr5	1.54E+08	1.54E+08	+	2	0	10	0	9	0	1	0	6	3	miRNA
hsa-miR-4299	chr11	11678208	11678225	-	2	0	1	0	6	0	0	0	1	0	miRNA
hsa-miR-4300	chr11	81601793	81601810	-	2	16	1	2	0	1	0	0	0	1	miRNA
hsa-miR-4466	chr6	1.57E+08	1.57E+08	-	2	0	0	1	6	5	0	0	1	1	miRNA
hsa-miR-4500	chr13	88270926	88270942	-	2	1	10	0	5	0	0	0	2	10	miRNA
hsa-miR-514a-5p	chrX	1.46E+08	1.46E+08	-	2	1	0	2	14	3	0	0	0	5	miRNA
hsa-miR-6131	chr5	10478219	10478237	+	2	1	0	1	2	1	2	0	3	5	miRNA
hsa-miR-6134	chrX	28513692	28513710	-	2	9	23	3	14	5	9	0	8	13	miRNA
hsa-miR-7704	chr2	1.77E+08	1.77E+08	+	2	0	0	3	0	29	0	0	0	0	miRNA
hsa-mir-23b	chr9	97847490	97847586	+	2	80	190	1	418	35	31	1	15	43	miRNA
hsa-mir-23c	chrX	20035206	20035305	-	2	0	3	1	4	0	0	0	20	0	miRNA
hsa-mir-378h	chr5	1.54E+08	1.54E+08	+	2	0	10	0	9	0	1	0	6	3	miRNA
hsa-mir-4299	chr11	11678198	11678269	-	2	0	1	0	6	0	0	0	1	0	miRNA
hsa-mir-4300	chr11	81601783	81601878	-	2	16	1	2	0	1	0	0	0	1	miRNA
hsa-mir-4466	chr6	1.57E+08	1.57E+08	-	2	0	0	1	6	5	0	0	1	1	miRNA
hsa-mir-4500	chr13	88270920	88270995	-	2	1	10	0	5	0	0	0	2	10	miRNA
hsa-mir-4517	chr16	28969904	28969982	+	2	0	0	0	0	0	0	0	0	1	miRNA
hsa-mir-514a-1	chrX	1.46E+08	1.46E+08	-	2	1	0	2	14	3	0	0	0	5	miRNA
hsa-mir-514a-2	chrX	1.46E+08	1.46E+08	-	2	1	0	2	14	3	0	0	0	5	miRNA
hsa-mir-514a-3	chrX	1.46E+08	1.46E+08	-	2	1	0	2	14	3	0	0	0	5	miRNA
hsa-mir-6131	chr5	10478149	10478257	+	2	1	0	1	2	1	2	0	3	5	miRNA
hsa-mir-6134	chrX	28513672	28513780	-	2	9	23	3	14	5	9	0	8	13	miRNA
hsa-mir-7704	chr2	1.77E+08	1.77E+08	+	2	0	0	3	0	29	0	0	0	0	miRNA
hsa-miR-10401-5p	chrUn_gl0(	105999	106018	+	1	0	8	38	3	0	1	0	0	0	miRNA
hsa-miR-125b-1-3p	chr11	1.22E+08	1.22E+08	-	1	0	0	0	0	0	0	0	0	0	miRNA
hsa-miR-1263	chr3	1.64E+08	1.64E+08	-	1	0	0	0	0	0	0	0	0	0	miRNA
hsa-miR-1289	chr5	1.33E+08	1.33E+08	-	1	0	0	0	0	0	0	0	1	0	miRNA
hsa-miR-150-5p	chr19	50004089	50004110	-	1	27	111	53	119	59	0	0	37	0	miRNA
hsa-miR-200a-5p	chr1	1103258	1103279	+	1	0	34	0	0	1	0	0	0	0	miRNA
hsa-miR-218-1-3p	chr4	20529965	20529986	+	1	0	0	0	0	0	0	0	0	0	miRNA
hsa-miR-218-5p	chr5	1.68E+08	1.68E+08	-	1	0	113	0	4	0	0	0	0	0	miRNA
hsa-miR-30b-5p	chr8	1.36E+08	1.36E+08	-	1	78	29	0	146	47	44	0	24	1	miRNA
hsa-miR-3124-5p	chr1	2.49E+08	2.49E+08	+	1	0	0	0	31	0	39	0	1	17	miRNA
hsa-miR-33a-5p	chr22	42296953	42296973	+	1	81	158	85	27	0	0	45	44	129	miRNA
hsa-miR-378b	chr3	10371946	10371964	+	1	2	4	2	0	0	0	0	3	2	miRNA
hsa-miR-4318	chr18	35237152	35237168	+	1	0	1	1	0	0	0	0	0	0	miRNA
hsa-miR-4433a-3p	chr2	64567943	64567963	+	1	0	0	0	0	0	0	0	0	0	miRNA
hsa-miR-4448	chr3	1.84E+08	1.84E+08	+	1	0	0	0	0	0	0	0	0	0	miRNA
hsa-miR-4658	chr7	99754234	99754256	-	1	0	0	0	0	0	0	0	0	0	miRNA
hsa-miR-4664-3p	chr8	1.45E+08	1.45E+08	-	1	0	0	0	0	0	0	0	0	0	miRNA
hsa-miR-4682	chr10	1.22E+08	1.22E+08	+	1	0	0	0	0	0	0	0	0	0	miRNA
hsa-miR-4693-3p	chr11	1.04E+08	1.04E+08	+	1	0	0	0	6	0	0	0	0	0	miRNA
hsa-miR-548ae-3p	chr5	57825874	57825894	-	1	0	0	0	0	0	0	0	0	0	miRNA
hsa-miR-548au-5p	chr9	96357123	96357143	+	1	9	236	0	17	39	41	0	75	45	miRNA
hsa-miR-550a-3p	chr7	32772653	32772674	+	1	0	48	27	15	0	27	0	128	99	miRNA
hsa-miR-603	chr10	24564674	24564695	+	1	0	0	0	0	0	0	0	0	0	miRNA
hsa-miR-6124	chr11	12185239	12185258	+	1	0	0	1	0	0	0	0	0	0	miRNA
hsa-miR-615-3p	chr12	54427794	54427815	+	1	0	0	2	0	0	1	0	1	2	miRNA
hsa-miR-6729-5p	chr1	12089220	12089241	+	1	0	0	0	0	0	0	0	10	0	miRNA

hsa-miR-6753-5p	chr11	67812261	67812282	+	1	0	0	4	1	0	25	0	0	0	miRNA
hsa-miR-6760-5p	chr12	1.12E+08	1.12E+08	+	1	0	0	0	0	0	0	0	0	0	miRNA
hsa-miR-6773-5p	chr16	68267375	68267397	-	1	0	0	0	0	0	0	0	0	0	miRNA
hsa-miR-6787-5p	chr17	80194549	80194570	+	1	0	0	0	0	0	0	0	0	0	miRNA
hsa-miR-761	chr1	52302047	52302068	-	1	0	0	1	1	3	0	0	0	0	miRNA
hsa-miR-765	chr1	1.57E+08	1.57E+08	-	1	0	16	2	0	0	2	0	0	47	miRNA
hsa-mir-10401	chrUn_gl0(	105999	106054	+	1	0	8	38	3	0	1	0	0	0	miRNA
hsa-mir-125b-1	chr11	1.22E+08	1.22E+08	-	1	0	76	0	7	14	0	0	0	0	miRNA
hsa-mir-1263	chr3	1.64E+08	1.64E+08	-	1	0	0	0	0	0	0	0	0	0	miRNA
hsa-mir-1286	chr22	20236657	20236734	-	1	0	0	0	7	0	0	0	0	0	miRNA
hsa-mir-1289-2	chr5	1.33E+08	1.33E+08	-	1	0	0	0	0	0	0	0	1	0	miRNA
hsa-mir-218-1	chr4	20529898	20530007	+	1	0	113	0	4	0	0	0	0	0	miRNA
hsa-mir-218-2	chr5	1.68E+08	1.68E+08	-	1	0	113	0	4	0	0	0	0	0	miRNA
hsa-mir-30b	chr8	1.36E+08	1.36E+08	-	1	95	118	72	321	90	173	0	73	48	miRNA
hsa-mir-3124	chr1	2.49E+08	2.49E+08	+	1	0	0	0	31	0	39	0	1	17	miRNA
hsa-mir-33a	chr22	42296948	42297016	+	1	81	158	85	42	0	0	45	44	129	miRNA
hsa-mir-3648-1	chrUn_gl0(	107909	108088	+	1	0	0	0	0	0	22	0	0	0	miRNA
hsa-mir-3648-2	chr21	9825832	9826011	+	1	0	0	0	0	0	22	0	20	0	miRNA
hsa-mir-378b	chr3	10371913	10371969	+	1	2	4	2	9	0	0	0	3	2	miRNA
hsa-mir-4309	chr14	1.03E+08	1.03E+08	+	1	0	1	0	3	3	0	0	0	0	miRNA
hsa-mir-4318	chr18	35237098	35237178	+	1	0	1	1	0	0	0	0	0	0	miRNA
hsa-mir-4433a	chr2	64567893	64567973	+	1	0	0	0	0	0	0	0	0	0	miRNA
hsa-mir-4448	chr3	1.84E+08	1.84E+08	+	1	16	0	0	0	0	0	0	0	0	miRNA
hsa-mir-4449	chr4	53578849	53578914	+	1	12	0	0	2	0	11	0	20	0	miRNA
hsa-mir-4658	chr7	99754228	99754292	-	1	0	0	0	0	0	0	0	0	0	miRNA
hsa-mir-4664	chr8	1.45E+08	1.45E+08	-	1	0	0	0	0	0	0	0	0	0	miRNA
hsa-mir-4682	chr10	1.22E+08	1.22E+08	+	1	0	0	0	0	0	0	0	0	0	miRNA
hsa-mir-4693	chr11	1.04E+08	1.04E+08	+	1	0	0	0	6	0	0	0	0	0	miRNA
hsa-mir-4706	chr14	65511406	65511487	+	1	0	0	1	0	0	0	0	17	0	miRNA
hsa-mir-4751	chr19	50436321	50436394	+	1	0	0	0	0	0	0	0	0	0	miRNA
hsa-mir-548au	chr9	96357123	96357176	+	1	9	243	0	17	39	41	0	75	45	miRNA
hsa-mir-550a-2	chr7	32772593	32772689	+	1	0	106	28	55	0	27	0	181	193	miRNA
hsa-mir-5692c-1	chr5	1.35E+08	1.35E+08	-	1	0	1	0	15	0	2	0	0	0	miRNA
hsa-mir-603	chr10	24564614	24564710	+	1	0	0	0	0	0	0	0	0	0	miRNA
hsa-mir-6124	chr11	12185230	12185314	+	1	0	0	1	0	0	0	0	0	0	miRNA
hsa-mir-615	chr12	54427734	54427829	+	1	0	0	2	9	0	1	0	1	2	miRNA
hsa-mir-628	chr15	55665138	55665232	-	1	0	29	0	14	0	0	0	13	50	miRNA
hsa-mir-6729	chr1	12089215	12089279	+	1	0	0	0	0	0	0	0	10	0	miRNA
hsa-mir-6753	chr11	67812261	67812424	+	1	0	0	4	3	0	29	0	0	0	miRNA
hsa-mir-6760	chr12	1.12E+08	1.12E+08	+	1	0	0	0	0	0	0	0	0	0	miRNA
hsa-mir-6773	chr16	68267329	68267402	-	1	0	0	0	0	0	0	0	0	0	miRNA
hsa-mir-6787	chr17	80194544	80194604	+	1	0	0	0	0	0	0	0	0	0	miRNA
hsa-mir-6884	chr17	38182585	38182662	-	1	0	57	0	0	0	0	0	37	0	miRNA
hsa-mir-7111	chr6	35438285	35438356	+	1	0	0	0	0	0	0	0	0	0	miRNA
hsa-mir-761	chr1	52302016	52302074	-	1	0	0	1	1	3	0	0	0	0	miRNA
hsa-mir-765	chr1	1.57E+08	1.57E+08	-	1	0	17	2	0	0	2	0	0	47	miRNA
hsa-mir-9902-1	chr12	1.23E+08	1.23E+08	-	1	0	0	0	0	0	0	0	0	0	miRNA
hsa-mir-9902-2	chr12	1.23E+08	1.23E+08	-	1	0	0	0	0	0	0	0	0	0	miRNA
hsa-miR-100-5p	chr11	1.22E+08	1.22E+08	-	0	8	346	0	0	1	2	124	1	82	miRNA
hsa-miR-101-2-5p	chr9	4850308	4850329	+	0	0	0	0	0	0	0	0	18	0	miRNA
hsa-miR-101-5p	chr1	65524160	65524181	-	0	29	671	0	46	24	136	0	365	156	miRNA
hsa-miR-10226	chr17	46110258	46110279	+	0	0	0	0	0	0	1	1	0	0	miRNA
hsa-miR-10393-3p	chr15	45010070	45010091	+	0	0	0	0	18	0	0	0	15	0	miRNA
hsa-miR-10393-5p	chr15	45010042	45010066	+	0	0	0	60	26	2	1	0	0	0	miRNA
hsa-miR-10396a-3p	chr21	9826478	9826498	+	0	0	0	35	0	0	0	0	0	1	miRNA
hsa-miR-10397-5p	chr5	10402476	10402497	+	0	1	0	0	0	0	0	0	0	0	miRNA
hsa-miR-10399-3p	chr7	1.39E+08	1.39E+08	-	0	32	68	8	149	40	72	69	28	52	miRNA
hsa-miR-10399-5p	chr7	1.39E+08	1.39E+08	-	0	0	0	0	20	0	0	0	0	0	miRNA
hsa-miR-106a-3p	chrX	1.33E+08	1.33E+08	-	0	0	41	0	0	1	0	0	50	0	miRNA
hsa-miR-10a-3p	chr17	46657226	46657247	-	0	0	120	0	12	0	56	0	0	0	miRNA
hsa-miR-10b-3p	chr2	1.77E+08	1.77E+08	+	0	0	32	0	0	0	0	0	0	0	miRNA
hsa-miR-11400	chr7	1.42E+08	1.42E+08	-	0	171	49	53	297	38	22	0	19	82	miRNA
hsa-miR-11401	chr16	87887512	87887531	+	0	0	35	0	0	0	0	0	195	0	miRNA
hsa-miR-1179	chr15	89151352	89151372	+	0	0	30	57	15	0	0	0	0	0	miRNA
hsa-miR-1185-1-3p	chr14	1.02E+08	1.02E+08	+	0	14	0	57	37	0	0	0	10	0	miRNA
hsa-miR-1185-2-3p	chr14	1.02E+08	1.02E+08	+	0	14	0	0	18	0	0	0	1	0	miRNA
hsa-miR-1199-5p	chr19	14184193	14184212	+	0	0	0	0	0	0	1	0	0	0	miRNA
hsa-miR-1207-3p	chr8	1.29E+08	1.29E+08	+	0	23	0	0	0	0	0	0	0	0	miRNA
hsa-miR-12114	chr22	50845885	50845903	+	0	0	0	0	0	0	0	0	24	0	miRNA
hsa-miR-12120	chrY	15591069	15591094	+	0	0	0	0	0	56	22	0	0	0	miRNA
hsa-miR-12125	chr5	1.68E+08	1.68E+08	+	0	1	1	1	0	0	1	0	0	1	miRNA
hsa-miR-12135	chr15	73592303	73592320	+	0	0	0	0	6	0	0	0	0	0	miRNA
hsa-miR-122-3p	chr18	56118356	56118377	+	0	0	0	0	11	0	0	0	0	0	miRNA
hsa-miR-1224-5p	chr3	1.84E+08	1.84E+08	+	0	24	102	0	9	0	37	0	43	0	miRNA
hsa-miR-1226-3p	chr3	47891098	47891119	+	0	0	0	0	6	0	0	0	0	0	miRNA
hsa-miR-1226-5p	chr3	47891045	47891070	+	0	0	1	0	0	0	0	0	21	0	miRNA
hsa-miR-1229-3p	chr5	1.79E+08	1.79E+08	-	0	0	0	0	12	0	0	0	0	0	miRNA
hsa-miR-1229-5p	chr5	1.79E+08	1.79E+08	-	0	0	0	0	0	0	0	1	0	0	miRNA
hsa-miR-122b-5p	chr18	56118353	56118375	-	0	0	1	0	0	0	0	0	0	1	miRNA
hsa-miR-1236-5p	chr6	31924695	31924716	-	0	0	0	0	0	0	22	0	40	0	miRNA
hsa-miR-1237-5p	chr11	64136123	64136143	+	0	1	0	0	0	0	0	0	0	0	miRNA
hsa-miR-124-3p	chr20	61809904	61809925	+	0	0	0	0	0	0	0	0	0	3	miRNA
hsa-miR-1247-5p	chr14	1.02E+08	1.02E+08	-	0	0	0	0	0	0	0	0	18	0	miRNA

hsa-miR-1248	chr3	1.87E+08	1.87E+08	+	0	0	0	0	10	35	0	0	0	0	miRNA
hsa-miR-1250-3p	chr17	79107028	79107046	-	0	0	0	0	0	0	0	0	0	1	miRNA
hsa-miR-1250-5p	chr17	79107065	79107085	-	0	13	0	0	6	0	0	0	0	0	miRNA
hsa-miR-1255a	chr4	1.02E+08	1.02E+08	-	0	0	44	0	0	0	1	0	1	0	miRNA
hsa-miR-1258	chr2	1.81E+08	1.81E+08	-	0	0	0	0	7	0	0	0	0	0	miRNA
hsa-miR-125b-2-3p	chr21	17962610	17962631	+	0	40	112	0	3	0	0	118	0	0	miRNA
hsa-miR-125b-5p	chr11	1.22E+08	1.22E+08	-	0	0	76	0	7	14	0	0	0	0	miRNA
hsa-miR-1260b	chr11	96074611	96074629	+	0	7	0	0	172	1	0	0	1	6	miRNA
hsa-miR-1262	chr1	68649259	68649280	-	0	20	0	0	13	0	0	0	7	0	miRNA
hsa-miR-1268b	chr17	78072630	78072649	+	0	0	0	0	0	0	0	0	1	0	miRNA
hsa-miR-127-5p	chr14	1.01E+08	1.01E+08	+	0	14	0	67	24	0	0	39	0	48	miRNA
hsa-miR-1275	chr6	33967795	33967811	-	0	0	31	2	16	26	24	0	0	0	miRNA
hsa-miR-1277-5p	chrX	1.18E+08	1.18E+08	+	0	0	0	1	0	0	0	0	0	0	miRNA
hsa-miR-128-1-5p	chr2	1.36E+08	1.36E+08	+	0	0	10	0	15	0	0	0	19	23	miRNA
hsa-miR-1285-3p	chr2	70480065	70480086	-	0	23	142	0	0	0	0	0	91	0	miRNA
hsa-miR-1285-5p	chr7	91833381	91833401	-	0	0	15	0	0	0	0	0	0	0	miRNA
hsa-miR-1288-5p	chr17	16185342	16185364	+	0	18	0	0	0	0	0	0	0	0	miRNA
hsa-miR-129-1-3p	chr7	1.28E+08	1.28E+08	+	0	0	0	0	1	0	0	0	0	0	miRNA
hsa-miR-129-5p	chr11	43602958	43602978	+	0	0	1	0	8	0	2	0	0	0	miRNA
hsa-miR-1295a	chr1	1.71E+08	1.71E+08	-	0	0	0	0	14	0	0	0	0	0	miRNA
hsa-miR-1295b-3p	chr1	1.71E+08	1.71E+08	+	0	0	0	0	0	0	1	0	0	0	miRNA
hsa-miR-1296-5p	chr10	65132772	65132793	-	0	0	0	0	6	0	0	0	0	0	miRNA
hsa-miR-1299	chr9	69002239	69002260	-	0	0	240	0	0	0	153	0	61	0	miRNA
hsa-miR-1301-3p	chr2	25551520	25551543	-	0	0	88	0	12	0	41	0	4	20	miRNA
hsa-miR-1303	chr5	1.54E+08	1.54E+08	+	0	0	26	0	5	24	16	0	27	14	miRNA
hsa-miR-1304-3p	chr11	93466857	93466878	-	0	49	118	138	166	0	0	0	77	111	miRNA
hsa-miR-1306-3p	chr22	20073635	20073652	+	0	0	22	0	39	0	0	0	5	70	miRNA
hsa-miR-1306-5p	chr22	20073595	20073616	+	0	20	0	0	14	0	0	0	51	0	miRNA
hsa-miR-130a-5p	chr11	57408685	57408706	+	0	0	0	0	13	0	1	0	0	0	miRNA
hsa-miR-132-3p	chr17	1953223	1953244	-	0	53	180	35	115	25	30	3	97	60	miRNA
hsa-miR-132-5p	chr17	1953259	1953280	-	0	0	0	0	0	0	1	0	0	0	miRNA
hsa-miR-1321	chrX	85090837	85090854	+	0	0	0	0	0	0	0	2	0	0	miRNA
hsa-miR-133a-3p	chr18	19405673	19405694	-	0	36	42	1	59	89	0	0	46	103	miRNA
hsa-miR-133a-5p	chr18	19405710	19405731	-	0	0	21	0	0	0	0	0	0	0	miRNA
hsa-miR-133b	chr6	52013786	52013807	+	0	0	0	0	0	2	0	0	2	4	miRNA
hsa-miR-136-3p	chr14	1.01E+08	1.01E+08	+	0	24	10	93	31	0	0	0	0	29	miRNA
hsa-miR-136-5p	chr14	1.01E+08	1.01E+08	+	0	0	0	0	7	0	0	0	0	0	miRNA
hsa-miR-137-3p	chr1	98511647	98511669	-	0	0	0	0	9	0	0	1	0	0	miRNA
hsa-miR-138-1-3p	chr3	44155766	44155787	+	0	0	0	0	0	0	0	0	3	0	miRNA
hsa-miR-139-3p	chr11	72326110	72326132	-	0	25	11	41	48	0	17	0	7	0	miRNA
hsa-miR-139-5p	chr11	72326146	72326168	-	0	45	56	0	300	92	40	0	5	196	miRNA
hsa-miR-141-3p	chr12	7073318	7073339	+	0	0	119	0	10	0	0	38	0	0	miRNA
hsa-miR-143-5p	chr5	1.49E+08	1.49E+08	+	0	0	0	0	18	0	0	0	0	0	miRNA
hsa-miR-145-3p	chr5	1.49E+08	1.49E+08	+	0	0	30	0	0	27	29	0	6	0	miRNA
hsa-miR-145-5p	chr5	1.49E+08	1.49E+08	+	0	0	278	0	15	2	41	0	28	23	miRNA
hsa-miR-146a-3p	chr5	1.6E+08	1.6E+08	+	0	0	0	0	1	0	0	0	0	0	miRNA
hsa-miR-146b-3p	chr10	1.04E+08	1.04E+08	+	0	0	22	0	29	42	12	0	46	0	miRNA
hsa-miR-147b-3p	chr15	45725296	45725316	+	0	0	32	0	6	0	0	0	0	0	miRNA
hsa-miR-149-3p	chr2	2.41E+08	2.41E+08	+	0	0	0	0	0	0	1	0	0	0	miRNA
hsa-miR-152-5p	chr17	46114576	46114598	-	0	0	0	0	0	0	0	0	0	1	miRNA
hsa-miR-153-3p	chr2	2.2E+08	2.2E+08	-	0	0	0	0	0	0	0	0	0	23	miRNA
hsa-miR-1538	chr16	69599711	69599733	-	0	4	0	0	5	0	0	0	25	0	miRNA
hsa-miR-1539	chr18	47013772	47013792	+	0	0	0	0	0	0	1	0	0	0	miRNA
hsa-miR-154-5p	chr14	1.02E+08	1.02E+08	+	0	0	9	75	45	0	0	0	26	0	miRNA
hsa-miR-15a-3p	chr13	50623266	50623287	-	0	0	1	0	0	0	0	0	0	0	miRNA
hsa-miR-15b-3p	chr3	1.6E+08	1.6E+08	+	0	65	428	111	118	40	164	0	366	0	miRNA
hsa-miR-16-1-3p	chr13	50623121	50623142	-	0	0	0	0	4	0	17	0	24	0	miRNA
hsa-miR-17-3p	chr13	92002909	92002930	+	0	71	1940	55	136	61	572	0	608	447	miRNA
hsa-miR-181b-2-3p	chr9	1.27E+08	1.27E+08	+	0	0	1	0	0	0	0	0	0	0	miRNA
hsa-miR-181b-3p	chr1	1.99E+08	1.99E+08	-	0	0	0	0	7	0	0	0	0	0	miRNA
hsa-miR-181c-3p	chr19	13985577	13985598	+	0	23	69	56	55	47	0	0	0	92	miRNA
hsa-miR-182-3p	chr7	1.29E+08	1.29E+08	-	0	0	0	0	12	0	0	0	0	0	miRNA
hsa-miR-184	chr15	79502182	79502203	+	0	0	0	0	7	0	0	0	0	0	miRNA
hsa-miR-185-3p	chr22	20020711	20020732	+	0	68	116	87	85	37	37	1	67	89	miRNA
hsa-miR-188-5p	chrX	49768123	49768143	+	0	0	33	0	11	0	0	0	0	0	miRNA
hsa-miR-18a-3p	chr13	92003051	92003073	+	0	0	81	23	24	0	86	0	85	35	miRNA
hsa-miR-18a-5p	chr13	92003010	92003032	+	0	268	446	0	404	77	122	0	514	357	miRNA
hsa-miR-18b-3p	chrX	1.33E+08	1.33E+08	-	0	18	16	0	0	0	0	0	0	0	miRNA
hsa-miR-18b-5p	chrX	1.33E+08	1.33E+08	-	0	74	364	0	54	1	122	0	362	164	miRNA
hsa-miR-190a-5p	chr15	63116170	63116191	+	0	61	0	0	13	0	21	0	19	15	miRNA
hsa-miR-1910-3p	chr16	85775237	85775256	-	0	0	2	0	0	0	0	0	0	0	miRNA
hsa-miR-1910-5p	chr16	85775275	85775295	-	0	0	23	0	0	0	0	0	0	0	miRNA
hsa-miR-193b-3p	chr16	14397874	14397895	+	0	0	0	0	13	0	9	0	0	0	miRNA
hsa-miR-193b-5p	chr16	14397837	14397858	+	0	0	196	32	41	12	32	0	0	36	miRNA
hsa-miR-194-3p	chr11	64658840	64658861	-	0	25	0	0	16	0	0	0	0	40	miRNA
hsa-miR-194-5p	chr1	2.2E+08	2.2E+08	-	0	20	195	17	110	0	1	0	18	0	miRNA
hsa-miR-195-3p	chr17	6920947	6920968	-	0	33	46	0	5	1	0	46	0	0	miRNA
hsa-miR-195-5p	chr17	6920986	6921006	-	0	26	12	0	2	1	2	0	9	4	miRNA
hsa-miR-196a-5p	chr12	54385546	54385567	+	0	24	55	0	25	0	38	0	0	0	miRNA
hsa-miR-196b-5p	chr7	27209147	27209168	-	0	101	792	59	207	49	99	0	190	274	miRNA
hsa-miR-197-3p	chr1	1.1E+08	1.1E+08	+	0	35	0	0	111	21	4	0	5	128	miRNA
hsa-miR-197-5p	chr1	1.1E+08	1.1E+08	+	0	0	133	0	31	0	97	0	66	9	miRNA
hsa-miR-199a-5p	chr1	1.72E+08	1.72E+08	-	0	0	23	0	50	0	0	0	0	0	miRNA

hsa-miR-199b-5p	chr9	1.31E+08	1.31E+08	-	0	0	10	0	21	0	0	0	0	0	miRNA
hsa-miR-19a-5p	chr13	92003158	92003179	+	0	0	0	0	0	0	0	0	0	1	miRNA
hsa-miR-200b-3p	chr1	1102540	1102561	+	0	22	0	64	13	0	0	0	0	0	miRNA
hsa-miR-200b-5p	chr1	1102504	1102525	+	0	0	38	0	10	0	0	0	0	0	miRNA
hsa-miR-200c-3p	chr12	7072905	7072927	+	0	43	58	99	218	122	59	0	14	66	miRNA
hsa-miR-202-3p	chr10	1.35E+08	1.35E+08	-	0	0	0	0	0	3	0	0	7	0	miRNA
hsa-miR-203a-5p	chr14	1.05E+08	1.05E+08	+	0	0	0	0	21	0	0	0	0	0	miRNA
hsa-miR-204-5p	chr9	73424947	73424968	-	0	19	0	0	12	0	0	0	0	0	miRNA
hsa-miR-205-5p	chr1	2.1E+08	2.1E+08	+	0	19	129	61	23	0	38	0	28	62	miRNA
hsa-miR-208b-3p	chr14	23887206	23887227	-	0	0	159	0	0	0	47	0	0	0	miRNA
hsa-miR-20a-3p	chr13	92003362	92003383	+	0	0	0	0	7	0	0	0	0	0	miRNA
hsa-miR-20b-3p	chrX	1.33E+08	1.33E+08	-	0	0	0	0	0	0	0	0	18	22	miRNA
hsa-miR-21-3p	chr17	57918672	57918692	+	0	66	219	60	48	87	0	41	72	133	miRNA
hsa-miR-2110	chr10	1.16E+08	1.16E+08	-	0	22	106	24	69	26	68	0	91	44	miRNA
hsa-miR-2116-3p	chr15	59463391	59463411	-	0	0	0	0	0	0	0	0	0	19	miRNA
hsa-miR-212-5p	chr17	1953622	1953644	-	0	0	0	0	0	28	0	0	0	0	miRNA
hsa-miR-214-3p	chr1	1.72E+08	1.72E+08	-	0	0	23	0	0	0	0	0	0	0	miRNA
hsa-miR-214-5p	chr1	1.72E+08	1.72E+08	-	0	0	0	0	7	0	0	0	0	0	miRNA
hsa-miR-215-5p	chr1	2.2E+08	2.2E+08	-	0	2	68	5	18	46	10	2	5	6	miRNA
hsa-miR-216b-3p	chr2	56227859	56227882	-	0	1	18	1	0	1	18	2	0	2	miRNA
hsa-miR-217-5p	chr2	56210155	56210177	-	0	0	57	0	0	0	0	44	0	60	miRNA
hsa-miR-219a-1-3p	chr6	33175673	33175694	+	0	0	0	0	18	0	0	0	0	0	miRNA
hsa-miR-219b-3p	chr9	1.31E+08	1.31E+08	+	0	1	0	0	0	1	0	1	0	0	miRNA
hsa-miR-22-5p	chr17	1617246	1617267	-	0	26	316	0	193	147	44	0	230	177	miRNA
hsa-miR-221-5p	chrX	45605649	45605670	-	0	0	0	0	6	0	0	0	0	0	miRNA
hsa-miR-224-5p	chrX	1.51E+08	1.51E+08	-	0	0	15	0	31	0	0	0	0	0	miRNA
hsa-miR-2276-3p	chr13	24736608	24736629	+	0	0	0	0	0	0	49	0	0	0	miRNA
hsa-miR-2277-3p	chr5	92956416	92956436	-	0	10	62	0	6	0	0	0	0	24	miRNA
hsa-miR-2355-3p	chr2	2.08E+08	2.08E+08	-	0	0	0	0	22	0	0	0	15	0	miRNA
hsa-miR-2355-5p	chr2	2.08E+08	2.08E+08	-	0	0	0	0	4	0	0	0	0	0	miRNA
hsa-miR-2392	chr14	1.01E+08	1.01E+08	+	0	0	0	0	1	0	0	0	0	1	miRNA
hsa-miR-2467-5p	chr2	2.4E+08	2.4E+08	-	0	0	0	0	0	0	0	95	0	0	miRNA
hsa-miR-26a-2-3p	chr12	58218403	58218424	-	0	22	34	0	3	0	0	0	0	0	miRNA
hsa-miR-26b-3p	chr2	2.19E+08	2.19E+08	+	0	0	0	0	7	0	0	0	0	0	miRNA
hsa-miR-27b-5p	chr9	97847745	97847766	+	0	0	0	0	48	0	0	0	25	44	miRNA
hsa-miR-296-5p	chr20	57392716	57392736	-	0	0	1	0	2	0	0	0	20	0	miRNA
hsa-miR-299-3p	chr14	1.01E+08	1.01E+08	+	0	0	0	0	28	0	0	0	22	0	miRNA
hsa-miR-29b-1-5p	chr7	1.31E+08	1.31E+08	-	0	0	0	0	21	0	0	0	0	19	miRNA
hsa-miR-29b-3p	chr1	2.08E+08	2.08E+08	-	0	0	21	2	1	0	40	0	17	0	miRNA
hsa-miR-29c-5p	chr1	2.08E+08	2.08E+08	-	0	0	78	0	20	0	41	0	0	0	miRNA
hsa-miR-301b-3p	chr22	22007314	22007336	+	0	0	0	0	10	59	0	0	0	0	miRNA
hsa-miR-302e	chr11	7256002	7256018	+	0	0	0	0	0	28	0	0	24	130	miRNA
hsa-miR-3064-5p	chr17	62496935	62496955	-	0	0	0	0	0	0	0	0	30	0	miRNA
hsa-miR-3065-3p	chr17	79099726	79099748	+	0	0	0	0	3	58	0	0	0	0	miRNA
hsa-miR-3065-5p	chr17	79099686	79099708	+	0	0	23	0	7	0	0	0	0	0	miRNA
hsa-miR-3074-3p	chr9	97848306	97848327	-	0	0	0	0	0	2	0	0	0	0	miRNA
hsa-miR-3074-5p	chr9	97848345	97848365	-	0	0	0	0	8	0	0	0	13	37	miRNA
hsa-miR-30b-3p	chr8	1.36E+08	1.36E+08	-	0	17	89	72	175	43	129	0	49	47	miRNA
hsa-miR-30c-1-3p	chr1	41223011	41223032	+	0	33	45	62	37	0	0	0	9	52	miRNA
hsa-miR-30c-2-3p	chr6	72086667	72086688	-	0	16	0	0	25	0	0	0	0	0	miRNA
hsa-miR-30d-3p	chr8	1.36E+08	1.36E+08	-	0	0	0	0	17	0	46	0	46	0	miRNA
hsa-miR-31-3p	chr9	21512120	21512141	-	0	0	0	0	0	25	0	0	0	0	miRNA
hsa-miR-31-5p	chr9	21512157	21512177	-	0	26	25	0	37	0	0	0	3	78	miRNA
hsa-miR-3115	chr1	23370803	23370822	+	0	0	0	0	24	0	0	0	0	0	miRNA
hsa-miR-3117-3p	chr1	67094168	67094188	+	0	0	0	0	6	0	0	0	0	0	miRNA
hsa-miR-3120-3p	chr1	1.72E+08	1.72E+08	+	0	50	57	0	117	0	0	0	0	15	miRNA
hsa-miR-3122	chr1	2.12E+08	2.12E+08	+	0	0	0	0	0	0	0	0	6	0	miRNA
hsa-miR-3127-5p	chr2	97464025	97464047	+	0	0	95	0	7	0	0	0	95	0	miRNA
hsa-miR-3128	chr2	1.78E+08	1.78E+08	-	0	0	21	0	0	0	0	0	0	0	miRNA
hsa-miR-3129-5p	chr2	1.9E+08	1.9E+08	-	0	0	0	0	0	0	37	0	0	0	miRNA
hsa-miR-3130-5p	chr2	2.08E+08	2.08E+08	+	0	0	14	0	1	0	0	0	74	42	miRNA
hsa-miR-3131	chr2	2.2E+08	2.2E+08	-	0	0	38	0	0	0	0	0	0	0	miRNA
hsa-miR-3135a	chr3	20179066	20179087	+	0	0	33	0	0	0	0	0	0	0	miRNA
hsa-miR-3135b	chr6	32717729	32717750	-	0	1	0	0	0	0	0	1	0	1	miRNA
hsa-miR-3136-5p	chr3	69098155	69098177	-	0	0	36	0	6	0	0	0	21	0	miRNA
hsa-miR-3139	chr4	1.44E+08	1.44E+08	+	0	0	0	0	0	0	29	0	0	0	miRNA
hsa-miR-3143	chr6	27115408	27115432	+	0	20	66	0	0	0	1	24	93	0	miRNA
hsa-miR-3146	chr7	19744989	19745010	-	0	0	0	0	11	0	0	0	0	0	miRNA
hsa-miR-3150a-3p	chr8	96085190	96085211	+	0	0	0	0	14	0	0	0	17	0	miRNA
hsa-miR-3150b-3p	chr8	96085152	96085172	-	0	0	34	0	0	0	0	0	0	0	miRNA
hsa-miR-3153	chr9	91927189	91927211	+	0	0	0	0	0	0	0	0	9	1	miRNA
hsa-miR-3154	chr9	1.31E+08	1.31E+08	-	0	0	64	0	8	0	0	0	0	94	miRNA
hsa-miR-3157-3p	chr10	97824078	97824099	-	0	0	0	0	1	0	0	0	0	0	miRNA
hsa-miR-3157-5p	chr10	97824126	97824147	-	0	0	12	0	10	0	0	0	12	0	miRNA
hsa-miR-3158-3p	chr10	1.03E+08	1.03E+08	-	0	0	128	0	19	0	0	0	19	122	miRNA
hsa-miR-3160-3p	chr11	46473365	46473386	-	0	0	0	0	11	0	0	0	0	0	miRNA
hsa-miR-3164	chr11	68850653	68850674	+	0	0	71	0	12	0	0	0	23	0	miRNA
hsa-miR-3165	chr11	71783318	71783339	-	0	0	0	0	9	0	0	0	19	0	miRNA
hsa-miR-3173-3p	chr14	95604260	95604281	-	0	0	53	0	0	0	1	0	11	0	miRNA
hsa-miR-3173-5p	chr14	95604298	95604319	-	0	0	0	0	24	0	0	0	14	0	miRNA
hsa-miR-3174	chr15	90549996	90550018	+	0	0	0	0	22	0	0	0	0	0	miRNA
hsa-miR-3176	chr16	593336	593354	+	0	0	0	0	4	0	0	0	0	0	miRNA
hsa-miR-3178	chr16	2581980	2581996	-	0	1	1	0	0	0	0	0	0	0	miRNA

hsa-miR-3179	chr16	14995416	14995437	+	0	0	0	0	3	0	0	0	0	0	miRNA
hsa-miR-3180-5p	chr16	15005094	15005118	+	0	17	0	0	0	0	0	0	0	0	miRNA
hsa-miR-3182	chr16	83541954	83541970	+	0	0	0	1	3	1	0	0	1	0	miRNA
hsa-miR-3185	chr17	46801808	46801830	-	0	1	0	0	0	0	0	0	0	0	miRNA
hsa-miR-3186-5p	chr17	79418178	79418199	-	0	0	0	0	8	0	0	0	0	0	miRNA
hsa-miR-3187-3p	chr19	813627	813646	+	0	0	35	0	8	0	0	0	49	0	miRNA
hsa-miR-3190-3p	chr19	47730245	47730267	+	0	1	0	0	11	0	0	0	0	0	miRNA
hsa-miR-3191-3p	chr19	47730209	47730231	-	0	6	0	0	0	7	5	0	21	0	miRNA
hsa-miR-3192-3p	chr20	18451306	18451326	+	0	0	0	0	13	0	0	0	0	0	miRNA
hsa-miR-3192-5p	chr20	18451268	18451290	+	0	20	16	0	13	0	0	0	0	0	miRNA
hsa-miR-3193	chr20	30194989	30195010	+	0	0	0	0	0	1	0	0	0	0	miRNA
hsa-miR-3194-5p	chr20	50069485	50069505	-	0	0	0	0	0	0	0	0	11	0	miRNA
hsa-miR-3198	chr22	18246956	18246977	-	0	0	0	1	15	0	0	0	0	0	miRNA
hsa-miR-3199	chr22	28316523	28316545	+	0	0	19	0	0	0	0	0	0	0	miRNA
hsa-miR-32-3p	chr9	1.12E+08	1.12E+08	-	0	49	82	0	35	0	29	0	29	89	miRNA
hsa-miR-32-5p	chr9	1.12E+08	1.12E+08	-	0	22	504	0	30	0	100	0	103	98	miRNA
hsa-miR-3200-3p	chr22	31127597	31127618	+	0	0	47	0	13	0	163	0	107	0	miRNA
hsa-miR-3200-5p	chr22	31127556	31127577	+	0	0	0	1	0	0	0	0	0	0	miRNA
hsa-miR-3202	chrX	1.53E+08	1.53E+08	+	0	0	0	16	0	2	23	0	0	0	miRNA
hsa-miR-320a-5p	chr8	22102520	22102541	-	0	1	0	0	15	0	0	0	0	0	miRNA
hsa-miR-323a-5p	chr14	1.01E+08	1.01E+08	+	0	0	0	0	4	0	0	0	0	0	miRNA
hsa-miR-323b-3p	chr14	1.02E+08	1.02E+08	+	0	88	74	218	314	35	0	0	41	0	miRNA
hsa-miR-326	chr11	75046152	75046171	-	0	0	0	0	55	0	32	0	0	43	miRNA
hsa-miR-329-3p	chr14	1.01E+08	1.01E+08	+	0	0	0	61	0	0	0	0	0	0	miRNA
hsa-miR-329-5p	chr14	1.01E+08	1.01E+08	+	0	0	0	0	4	0	0	0	0	0	miRNA
hsa-miR-330-3p	chr19	46142267	46142289	-	0	0	30	0	105	45	0	0	0	4	miRNA
hsa-miR-330-5p	chr19	46142307	46142328	-	0	51	53	0	16	0	0	0	0	72	miRNA
hsa-miR-331-3p	chr12	95702256	95702276	+	0	0	37	0	3	0	0	0	0	0	miRNA
hsa-miR-331-5p	chr12	95702221	95702242	+	0	0	3	0	5	0	0	0	31	0	miRNA
hsa-miR-335-3p	chr7	1.3E+08	1.3E+08	+	0	8	34	19	12	38	0	0	0	51	miRNA
hsa-miR-337-3p	chr14	1.01E+08	1.01E+08	+	0	0	0	61	20	41	0	0	0	0	miRNA
hsa-miR-338-5p	chr17	79099723	79099744	-	0	0	0	0	37	0	0	0	0	0	miRNA
hsa-miR-33a-3p	chr22	42296993	42297014	+	0	0	0	0	15	0	0	0	0	0	miRNA
hsa-miR-33b-3p	chr17	17717171	17717192	-	0	0	0	0	10	0	0	0	0	0	miRNA
hsa-miR-340-3p	chr5	1.79E+08	1.79E+08	-	0	62	50	74	69	39	0	0	0	1	miRNA
hsa-miR-342-3p	chr14	1.01E+08	1.01E+08	+	0	0	31	114	26	40	82	0	18	0	miRNA
hsa-miR-346	chr10	88024504	88024526	-	0	1	0	0	0	0	0	0	0	0	miRNA
hsa-miR-34a-3p	chr1	9211752	9211773	-	0	0	0	0	2	0	0	0	0	0	miRNA
hsa-miR-34c-3p	chr11	1.11E+08	1.11E+08	+	0	0	47	0	0	0	0	0	0	0	miRNA
hsa-miR-34c-5p	chr11	1.11E+08	1.11E+08	+	0	73	26	55	23	0	0	10	34	0	miRNA
hsa-miR-3605-3p	chr1	33798006	33798028	-	0	22	34	0	31	0	37	0	24	85	miRNA
hsa-miR-3610	chr8	1.18E+08	1.18E+08	-	0	19	0	0	0	0	0	0	0	0	miRNA
hsa-miR-3614-3p	chr17	54968642	54968664	-	0	0	0	0	4	0	0	0	0	0	miRNA
hsa-miR-3614-5p	chr17	54968680	54968702	-	0	0	0	0	15	0	0	0	0	131	miRNA
hsa-miR-362-3p	chrX	49773613	49773634	+	0	0	23	0	9	0	30	0	3	61	miRNA
hsa-miR-3621	chr9	1.4E+08	1.4E+08	-	0	4	0	0	0	0	0	0	0	0	miRNA
hsa-miR-363-3p	chrX	1.33E+08	1.33E+08	-	0	9	1041	0	114	0	440	47	773	189	miRNA
hsa-miR-363-5p	chrX	1.33E+08	1.33E+08	-	0	0	29	0	0	0	0	0	0	0	miRNA
hsa-miR-3652	chr12	1.04E+08	1.04E+08	+	0	0	0	1	3	0	0	0	0	0	miRNA
hsa-miR-365a-3p	chr16	14403197	14403218	+	0	0	0	0	9	0	0	0	0	0	miRNA
hsa-miR-365a-5p	chr16	14403157	14403179	+	0	0	10	0	3	30	0	0	0	0	miRNA
hsa-miR-365b-3p	chr17	29902497	29902518	+	0	0	0	0	9	0	0	0	0	0	miRNA
hsa-miR-365b-5p	chr17	29902458	29902479	+	0	0	0	0	13	0	0	0	0	0	miRNA
hsa-miR-3661	chr5	1.34E+08	1.34E+08	+	0	0	0	0	0	0	0	1	0	0	miRNA
hsa-miR-3663-5p	chr10	1.19E+08	1.19E+08	-	0	0	0	0	0	0	1	0	0	0	miRNA
hsa-miR-3667-5p	chr22	49937085	49937106	-	0	0	0	0	2	0	0	0	0	0	miRNA
hsa-miR-3674	chr8	1749299	1749320	+	0	0	0	1	0	0	0	0	0	0	miRNA
hsa-miR-3679-5p	chr2	1.35E+08	1.35E+08	+	0	0	309	0	50	20	0	0	0	37	miRNA
hsa-miR-3680-3p	chr16	21517385	21517407	-	0	0	0	0	1	0	0	0	0	0	miRNA
hsa-miR-3682-3p	chr2	54076273	54076293	-	0	0	120	0	25	0	39	0	71	0	miRNA
hsa-miR-3688-3p	chr4	1.6E+08	1.6E+08	-	0	51	133	0	30	0	0	0	40	55	miRNA
hsa-miR-369-3p	chr14	1.02E+08	1.02E+08	+	0	15	165	67	152	0	36	1	0	1	miRNA
hsa-miR-369-5p	chr14	1.02E+08	1.02E+08	+	0	0	0	0	25	0	0	0	0	0	miRNA
hsa-miR-3691-5p	chr6	5148520	5148542	-	0	0	0	0	3	0	37	0	0	0	miRNA
hsa-miR-370-5p	chr14	1.01E+08	1.01E+08	+	0	0	0	0	40	0	0	0	0	0	miRNA
hsa-miR-371a-5p	chr19	54290934	54290953	+	0	0	0	0	4	0	0	0	0	0	miRNA
hsa-miR-371b-3p	chr19	54290932	54290954	-	0	0	0	0	0	0	0	0	1	0	miRNA
hsa-miR-372-3p	chr19	54291185	54291207	+	0	0	0	0	5	0	0	0	0	0	miRNA
hsa-miR-373-3p	chr19	54292002	54292024	+	0	0	38	0	0	1	0	68	0	0	miRNA
hsa-miR-374b-3p	chrX	73438392	73438413	-	0	23	0	0	9	0	0	0	0	0	miRNA
hsa-miR-374b-5p	chrX	73438422	73438443	-	0	46	43	0	133	37	41	0	7	0	miRNA
hsa-miR-374c-5p	chrX	73438396	73438417	+	0	0	1	0	1	0	1	0	0	0	miRNA
hsa-miR-376a-3p	chr14	1.02E+08	1.02E+08	+	0	16	0	0	34	36	0	0	0	73	miRNA
hsa-miR-376b-5p	chr14	1.02E+08	1.02E+08	+	0	0	0	0	1	0	0	0	0	0	miRNA
hsa-miR-376c-3p	chr14	1.02E+08	1.02E+08	+	0	0	0	0	10	0	0	0	0	0	miRNA
hsa-miR-376c-5p	chr14	1.02E+08	1.02E+08	+	0	0	0	0	1	0	0	0	0	0	miRNA
hsa-miR-378a-5p	chr5	1.49E+08	1.49E+08	+	0	0	0	0	6	0	0	0	19	0	miRNA
hsa-miR-378f	chr1	24255611	24255630	+	0	1	4	1	7	0	0	0	2	2	miRNA
hsa-miR-379-3p	chr14	1.01E+08	1.01E+08	+	0	0	0	0	14	0	0	0	0	0	miRNA
hsa-miR-380-5p	chr14	1.01E+08	1.01E+08	+	0	0	0	0	0	19	0	0	0	0	miRNA
hsa-miR-381-3p	chr14	1.02E+08	1.02E+08	+	0	45	24	110	291	99	0	52	15	104	miRNA
hsa-miR-381-5p	chr14	1.02E+08	1.02E+08	+	0	0	0	0	0	0	0	34	0	0	miRNA
hsa-miR-382-3p	chr14	1.02E+08	1.02E+08	+	0	20	34	5	106	0	43	0	16	0	miRNA

hsa-miR-3911	chr9	1.3E+08	1.3E+08	-	0	0	72	0	0	0	0	0	0	0	miRNA
hsa-miR-3912-3p	chr5	1.71E+08	1.71E+08	-	0	0	55	0	0	0	0	0	0	48	miRNA
hsa-miR-3913-5p	chr12	69978524	69978545	+	0	0	0	0	0	22	0	0	28	34	miRNA
hsa-miR-3918	chr6	1.59E+08	1.59E+08	-	0	0	0	0	3	0	0	0	0	0	miRNA
hsa-miR-3919	chr3	1.59E+08	1.59E+08	+	0	0	0	0	1	0	0	0	1	0	miRNA
hsa-miR-3922-3p	chr12	1.05E+08	1.05E+08	+	0	0	22	0	18	37	28	0	0	93	miRNA
hsa-miR-3922-5p	chr12	1.05E+08	1.05E+08	+	0	0	0	0	4	0	0	0	0	0	miRNA
hsa-miR-3923	chr3	79557087	79557108	+	0	1	0	0	0	0	0	0	0	0	miRNA
hsa-miR-3925-5p	chr6	36590257	36590278	-	0	0	11	0	0	0	0	0	0	0	miRNA
hsa-miR-3928-3p	chr22	31556048	31556069	-	0	0	59	0	38	6	0	0	0	0	miRNA
hsa-miR-3934-5p	chr6	33665929	33665950	+	0	0	11	0	0	0	0	0	18	0	miRNA
hsa-miR-3936	chr5	1.32E+08	1.32E+08	-	0	0	0	0	0	0	0	0	23	0	miRNA
hsa-miR-3937	chrX	39520530	39520552	+	0	0	0	0	4	2	0	0	0	0	miRNA
hsa-miR-3938	chr3	55886543	55886564	-	0	0	0	0	0	0	1	0	0	0	miRNA
hsa-miR-3939	chr6	1.67E+08	1.67E+08	-	0	0	0	0	5	0	0	0	0	0	miRNA
hsa-miR-3945	chr4	1.86E+08	1.86E+08	-	0	0	0	0	8	0	0	0	0	0	miRNA
hsa-miR-3960	chr9	1.31E+08	1.31E+08	+	0	1	0	0	0	1	0	0	0	0	miRNA
hsa-miR-3975	chr18	33171746	33171767	+	0	0	0	0	1	0	0	0	0	0	miRNA
hsa-miR-411-3p	chr14	1.01E+08	1.01E+08	+	0	0	0	0	10	0	0	0	0	0	miRNA
hsa-miR-411-5p	chr14	1.01E+08	1.01E+08	+	0	17	25	253	231	143	0	0	20	0	miRNA
hsa-miR-412-3p	chr14	1.02E+08	1.02E+08	+	0	0	0	0	0	1	0	0	0	0	miRNA
hsa-miR-412-5p	chr14	1.02E+08	1.02E+08	+	0	0	108	168	19	0	0	0	0	0	miRNA
hsa-miR-422a	chr15	64163188	64163209	-	0	1	5	0	1	0	0	0	1	2	miRNA
hsa-miR-424-5p	chrX	1.34E+08	1.34E+08	-	0	27	532	55	284	31	34	0	22	175	miRNA
hsa-miR-4271	chr3	49311591	49311609	+	0	0	0	26	0	0	0	0	0	0	miRNA
hsa-miR-4289	chr9	91360792	91360810	-	0	1	0	0	0	0	0	0	0	0	miRNA
hsa-miR-429	chr1	1104435	1104456	+	0	23	7	0	19	0	0	0	0	0	miRNA
hsa-miR-4307	chr14	27377903	27377921	+	0	0	0	0	0	0	1	0	0	0	miRNA
hsa-miR-4311	chr15	66332581	66332598	+	0	0	0	0	17	0	0	0	0	0	miRNA
hsa-miR-4320	chr18	47652879	47652896	-	0	0	0	0	1	0	0	0	0	0	miRNA
hsa-miR-4326	chr20	61918170	61918189	+	0	0	0	0	9	0	0	0	0	0	miRNA
hsa-miR-433-5p	chr14	1.01E+08	1.01E+08	+	0	0	0	0	6	0	0	0	0	0	miRNA
hsa-miR-4420	chr1	31212005	31212026	-	0	0	0	0	4	0	0	0	0	0	miRNA
hsa-miR-4427	chr1	2.34E+08	2.34E+08	+	0	0	0	0	0	0	1	0	0	0	miRNA
hsa-miR-4433b-5p	chr2	64567943	64567963	-	0	122	0	32	64	26	0	0	31	30	miRNA
hsa-miR-4434	chr2	64752654	64752671	+	0	0	0	0	0	0	0	1	0	0	miRNA
hsa-miR-4435	chr2	87929283	87929304	+	0	0	0	0	7	0	34	0	0	0	miRNA
hsa-miR-4436a	chr2	89111939	89111959	+	0	0	0	0	0	0	0	0	19	0	miRNA
hsa-miR-4436b-3p	chr2	1.11E+08	1.11E+08	-	0	0	0	0	10	0	0	0	0	0	miRNA
hsa-miR-4440	chr2	2.4E+08	2.4E+08	-	0	0	0	0	8	0	0	0	18	14	miRNA
hsa-miR-4441	chr2	2.4E+08	2.4E+08	-	0	0	0	0	6	0	0	0	0	0	miRNA
hsa-miR-4444	chr2	1.78E+08	1.78E+08	+	0	0	34	0	0	0	0	0	0	0	miRNA
hsa-miR-4445-5p	chr3	1.09E+08	1.09E+08	+	0	0	0	0	0	0	0	0	0	23	miRNA
hsa-miR-4454	chr4	1.64E+08	1.64E+08	-	0	0	29	0	0	1	37	31	0	31	miRNA
hsa-miR-4458	chr5	8461046	8461064	+	0	0	0	0	1	0	0	0	0	0	miRNA
hsa-miR-4467	chr7	1.02E+08	1.02E+08	+	0	0	50	0	0	0	0	0	15	0	miRNA
hsa-miR-4471	chr8	1.01E+08	1.01E+08	+	0	0	24	0	0	0	0	0	0	0	miRNA
hsa-miR-4473	chr9	20411159	20411180	-	0	0	0	0	7	0	0	0	0	0	miRNA
hsa-miR-4477b	chr9	68415357	68415378	+	0	0	0	0	6	0	1	0	0	85	miRNA
hsa-miR-4482-3p	chr10	1.06E+08	1.06E+08	-	0	0	18	0	0	0	0	0	1	0	miRNA
hsa-miR-4482-5p	chr10	1.06E+08	1.06E+08	-	0	0	0	0	1	0	0	0	0	0	miRNA
hsa-miR-4484	chr10	1.28E+08	1.28E+08	+	0	0	0	0	2	0	0	0	0	0	miRNA
hsa-miR-4485-5p	chr11	10529852	10529867	-	0	4	0	0	0	0	0	0	0	0	miRNA
hsa-miR-4486	chr11	19596861	19596877	+	0	0	0	0	0	0	0	0	29	1	miRNA
hsa-miR-4487	chr11	47422574	47422592	+	0	0	32	0	0	0	0	0	0	0	miRNA
hsa-miR-4488	chr11	61276071	61276088	+	0	0	0	0	0	0	0	42	0	0	miRNA
hsa-miR-4489	chr11	65416668	65416688	+	0	0	0	0	0	0	0	0	0	17	miRNA
hsa-miR-4492	chr11	1.19E+08	1.19E+08	+	0	0	0	0	0	0	1	0	17	0	miRNA
hsa-miR-4497	chr12	1.1E+08	1.1E+08	+	0	0	0	0	0	0	0	0	1	0	miRNA
hsa-miR-4498	chr12	1.21E+08	1.21E+08	-	0	0	13	0	0	0	0	0	0	0	miRNA
hsa-miR-4499	chr13	21007969	21007985	-	0	0	0	0	0	0	0	0	0	1	miRNA
hsa-miR-449a	chr5	54466414	54466435	-	0	12	0	0	9	0	0	0	16	22	miRNA
hsa-miR-449b-5p	chr5	54466534	54466555	-	0	1	0	0	0	0	0	0	0	0	miRNA
hsa-miR-450a-1-3p	chrX	1.34E+08	1.34E+08	-	0	0	0	0	9	0	0	0	0	0	miRNA
hsa-miR-450a-2-3p	chrX	1.34E+08	1.34E+08	-	0	61	68	0	137	0	39	0	29	79	miRNA
hsa-miR-450a-5p	chrX	1.34E+08	1.34E+08	-	0	0	32	0	0	0	0	0	0	0	miRNA
hsa-miR-450b-5p	chrX	1.34E+08	1.34E+08	-	0	0	0	0	0	48	0	0	23	61	miRNA
hsa-miR-4514	chr15	81289791	81289808	-	0	0	0	0	4	0	0	0	0	0	miRNA
hsa-miR-4517	chr16	28969908	28969932	+	0	0	0	0	0	0	0	0	0	1	miRNA
hsa-miR-4518	chr16	30515289	30515314	+	0	0	1	0	0	0	0	0	1	0	miRNA
hsa-miR-451b	chr17	27188395	27188416	+	0	0	6	0	0	0	0	0	0	4	miRNA
hsa-miR-452-3p	chrX	1.51E+08	1.51E+08	-	0	30	6	0	0	0	0	0	0	0	miRNA
hsa-miR-4520-2-3p	chr17	6558800	6558821	+	0	0	0	0	0	0	1	0	0	0	miRNA
hsa-miR-4520-3p	chr17	6558766	6558787	-	0	0	0	0	0	0	1	0	0	0	miRNA
hsa-miR-4521	chr17	8090266	8090287	+	0	3	1	2	9	0	1	0	1	6	miRNA
hsa-miR-4525	chr17	80626157	80626177	-	0	0	0	0	5	0	0	0	19	0	miRNA
hsa-miR-4526	chr18	13611166	13611187	+	0	0	49	0	0	0	0	0	0	0	miRNA
hsa-miR-455-3p	chr9	1.17E+08	1.17E+08	+	0	0	0	0	8	0	0	76	0	0	miRNA
hsa-miR-455-5p	chr9	1.17E+08	1.17E+08	+	0	0	0	0	0	0	0	0	1	0	miRNA
hsa-miR-4632-5p	chr1	12251770	12251792	+	0	0	11	0	0	0	0	0	0	0	miRNA
hsa-miR-4638-5p	chr5	1.81E+08	1.81E+08	-	0	0	0	0	8	0	0	0	0	0	miRNA
hsa-miR-4639-5p	chr6	16141787	16141808	+	0	0	26	0	0	0	0	0	0	0	miRNA
hsa-miR-4644	chr6	1.71E+08	1.71E+08	+	0	0	0	0	0	0	0	0	1	0	miRNA

hsa-miR-4646-5p	chr6	31668847	31668868	-	0	0	226	0	5	0	140	0	78	0	miRNA
hsa-miR-4647	chr6	44221990	44222012	-	0	0	0	0	0	0	0	0	14	0	miRNA
hsa-miR-4650-3p	chr7	66579319	66579339	-	0	0	1	0	0	0	0	0	0	0	miRNA
hsa-miR-4650-5p	chr7	66579352	66579370	-	0	0	1	0	0	0	0	0	0	0	miRNA
hsa-miR-4651	chr7	75544524	75544543	+	0	0	0	0	5	0	0	0	0	0	miRNA
hsa-miR-4653-3p	chr7	1.01E+08	1.01E+08	+	0	0	18	0	0	0	0	0	1	0	miRNA
hsa-miR-4655-5p	chr7	1883859	1883880	-	0	2	1	1	0	1	0	0	0	0	miRNA
hsa-miR-4656	chr7	4828239	4828261	-	0	93	0	0	0	0	0	0	0	0	miRNA
hsa-miR-4657	chr7	44921377	44921399	-	0	0	36	0	9	0	0	0	29	0	miRNA
hsa-miR-4659b-5p	chr8	6602734	6602752	-	0	0	37	0	0	0	0	0	0	0	miRNA
hsa-miR-4660	chr8	8905963	8905985	+	0	0	0	0	0	0	0	0	12	0	miRNA
hsa-miR-4665-5p	chr9	6007835	6007857	+	0	0	0	6	4	0	0	0	0	0	miRNA
hsa-miR-4667-5p	chr9	35608093	35608114	+	0	0	20	0	0	0	0	0	0	0	miRNA
hsa-miR-4669	chr9	1.37E+08	1.37E+08	+	0	0	23	0	11	0	0	0	159	0	miRNA
hsa-miR-4671-5p	chr1	2.34E+08	2.34E+08	+	0	0	0	0	0	0	1	0	0	1	miRNA
hsa-miR-4672	chr9	1.31E+08	1.31E+08	-	0	0	0	0	0	0	0	0	0	34	miRNA
hsa-miR-4673	chr9	1.39E+08	1.39E+08	-	0	0	0	0	0	0	0	0	6	0	miRNA
hsa-miR-4676-3p	chr10	74480830	74480851	+	0	0	0	0	2	0	0	0	0	0	miRNA
hsa-miR-4677-3p	chr1	2.44E+08	2.44E+08	+	0	24	0	0	2	0	0	0	0	0	miRNA
hsa-miR-4685-3p	chr10	1E+08	1E+08	-	0	0	0	56	1	0	0	0	0	0	miRNA
hsa-miR-4687-3p	chr11	3877343	3877363	+	0	0	0	0	2	0	0	0	1	0	miRNA
hsa-miR-4689	chr1	5922771	5922792	-	0	0	2	0	1	1	0	0	0	0	miRNA
hsa-miR-4690-5p	chr11	65403781	65403802	+	0	0	0	0	0	0	0	0	0	1	miRNA
hsa-miR-4695-3p	chr1	19209698	19209719	-	0	0	1	0	0	0	0	0	0	1	miRNA
hsa-miR-4706	chr14	65511415	65511439	+	0	0	0	1	0	0	0	0	17	0	miRNA
hsa-miR-4708-3p	chr14	65801841	65801862	-	0	0	0	0	0	0	0	0	11	0	miRNA
hsa-miR-4711-5p	chr1	60198941	60198963	-	0	0	0	0	6	0	0	0	0	0	miRNA
hsa-miR-4712-3p	chr15	50652582	50652602	+	0	0	0	0	0	0	0	0	0	1	miRNA
hsa-miR-4713-5p	chr15	51534397	51534418	+	0	0	0	0	1	0	0	0	0	0	miRNA
hsa-miR-4714-3p	chr15	99327702	99327723	+	0	0	19	0	3	0	0	0	0	0	miRNA
hsa-miR-4716-3p	chr15	49461276	49461297	-	0	0	59	0	0	0	0	0	1	0	miRNA
hsa-miR-4717-3p	chr16	2324662	2324682	+	0	0	0	0	8	1	0	0	0	30	miRNA
hsa-miR-4717-5p	chr16	2324630	2324651	+	0	0	0	0	0	1	0	0	0	0	miRNA
hsa-miR-4725-3p	chr17	29902345	29902366	+	0	0	0	0	9	0	0	0	1	0	miRNA
hsa-miR-4726-5p	chr17	36875944	36875966	+	0	0	0	0	0	0	0	0	12	0	miRNA
hsa-miR-4738-5p	chr17	73780648	73780669	-	0	0	18	0	0	0	0	0	0	0	miRNA
hsa-miR-4739	chr17	77681025	77681049	-	0	0	0	0	0	0	0	0	0	43	miRNA
hsa-miR-4740-3p	chr17	79374516	79374537	-	0	0	0	0	1	0	0	0	0	0	miRNA
hsa-miR-4742-3p	chr1	2.25E+08	2.25E+08	-	0	0	0	0	5	0	0	0	43	5	miRNA
hsa-miR-4743-5p	chr18	46196973	46196995	+	0	0	0	0	0	0	0	0	21	0	miRNA
hsa-miR-4745-3p	chr19	804977	804999	+	0	0	0	0	0	0	0	0	0	1	miRNA
hsa-miR-4746-3p	chr19	4446018	4446038	+	0	0	0	0	2	0	0	0	0	0	miRNA
hsa-miR-4746-5p	chr19	4445984	4446006	+	0	0	0	0	3	0	0	0	0	0	miRNA
hsa-miR-4747-5p	chr19	4932699	4932720	+	0	0	39	0	0	0	192	0	28	40	miRNA
hsa-miR-4748	chr19	10890939	10890959	+	0	0	0	0	0	0	0	0	39	0	miRNA
hsa-miR-4749-5p	chr19	50357850	50357871	+	0	0	0	0	0	26	0	0	0	0	miRNA
hsa-miR-4750-5p	chr19	50391434	50391455	+	0	0	1	36	4	0	0	0	0	46	miRNA
hsa-miR-4762-3p	chr22	46156452	46156473	+	0	0	0	0	14	0	6	0	0	0	miRNA
hsa-miR-4764-3p	chr22	33832574	33832595	-	0	0	0	0	4	0	0	0	0	0	miRNA
hsa-miR-4766-3p	chr22	41209897	41209917	-	0	0	0	0	7	0	107	0	0	0	miRNA
hsa-miR-4769-5p	chrX	47446835	47446858	+	0	0	0	0	12	0	0	0	0	0	miRNA
hsa-miR-4772-3p	chr2	1.03E+08	1.03E+08	+	0	0	0	0	76	0	0	0	0	108	miRNA
hsa-miR-4772-5p	chr2	1.03E+08	1.03E+08	+	0	0	0	137	16	0	0	0	0	0	miRNA
hsa-miR-4773	chr2	1.52E+08	1.52E+08	-	0	4	0	0	4	0	0	0	0	0	miRNA
hsa-miR-4776-3p	chr2	2.14E+08	2.14E+08	+	0	0	1	0	0	0	0	0	0	0	miRNA
hsa-miR-4784	chr2	1.32E+08	1.32E+08	-	0	0	0	0	0	0	13	0	21	0	miRNA
hsa-miR-4791	chr3	19356399	19356416	-	0	0	0	0	1	0	0	0	0	0	miRNA
hsa-miR-4795-3p	chr3	87275349	87275370	-	0	0	27	0	0	0	0	0	0	0	miRNA
hsa-miR-4796-3p	chr3	1.14E+08	1.14E+08	-	0	0	0	0	0	0	1	0	0	0	miRNA
hsa-miR-4800-5p	chr4	2251854	2251874	-	0	0	0	0	4	0	0	0	0	0	miRNA
hsa-miR-4804-5p	chr5	72174427	72174447	+	0	0	2	0	3	0	48	0	3	0	miRNA
hsa-miR-483-3p	chr11	2155372	2155392	-	0	0	0	0	10	0	0	0	0	0	miRNA
hsa-miR-485-3p	chr14	1.02E+08	1.02E+08	+	0	0	0	125	168	0	0	0	0	0	miRNA
hsa-miR-485-5p	chr14	1.02E+08	1.02E+08	+	0	0	0	0	8	0	0	0	0	0	miRNA
hsa-miR-487a-3p	chr14	1.02E+08	1.02E+08	+	0	0	0	0	0	0	0	0	17	0	miRNA
hsa-miR-487a-5p	chr14	1.02E+08	1.02E+08	+	0	0	0	0	21	0	0	0	18	0	miRNA
hsa-miR-487b-3p	chr14	1.02E+08	1.02E+08	+	0	30	35	294	504	0	0	0	41	0	miRNA
hsa-miR-489-3p	chr7	93113259	93113280	-	0	0	33	0	0	0	0	0	0	0	miRNA
hsa-miR-490-5p	chr7	1.37E+08	1.37E+08	+	0	0	0	0	0	0	0	0	20	0	miRNA
hsa-miR-493-3p	chr14	1.01E+08	1.01E+08	+	0	3	0	109	65	0	0	0	8	0	miRNA
hsa-miR-495-3p	chr14	1.02E+08	1.02E+08	+	0	0	0	0	30	0	0	0	0	0	miRNA
hsa-miR-496	chr14	1.02E+08	1.02E+08	+	0	0	0	0	2	0	0	0	0	0	miRNA
hsa-miR-497-5p	chr17	6921298	6921318	-	0	0	56	0	0	0	0	0	0	28	miRNA
hsa-miR-4999-5p	chr19	8454224	8454244	-	0	23	19	0	4	0	0	0	0	0	miRNA
hsa-miR-499a-5p	chr20	33578211	33578231	+	0	0	45	0	9	0	0	0	0	0	miRNA
hsa-miR-5000-3p	chr2	75318000	75318021	+	0	0	0	0	0	0	0	0	18	0	miRNA
hsa-miR-5001-3p	chr2	2.33E+08	2.33E+08	-	0	18	18	0	0	0	0	0	12	0	miRNA
hsa-miR-5003-3p	chr5	1.72E+08	1.72E+08	+	0	0	0	0	0	0	52	0	0	0	miRNA
hsa-miR-5003-5p	chr5	1.72E+08	1.72E+08	+	0	0	0	0	0	0	0	0	0	1	miRNA
hsa-miR-5006-5p	chr13	42142491	42142511	-	0	0	0	0	3	0	0	0	0	0	miRNA
hsa-miR-5009-5p	chr15	90427219	90427242	-	0	0	5	0	0	0	0	0	0	0	miRNA
hsa-miR-500a-3p	chrX	49773090	49773111	+	0	14	27	109	16	34	37	0	56	0	miRNA
hsa-miR-501-3p	chrX	49774380	49774401	+	0	0	76	0	0	0	0	51	0	0	miRNA

hsa-miR-501-5p	chrX	49774343	49774364	+	0	0	34	0	0	0	0	0	0	0	miRNA
hsa-miR-502-3p	chrX	49779257	49779278	+	0	14	27	109	16	34	37	0	56	0	miRNA
hsa-miR-502-5p	chrX	49779221	49779241	+	0	0	0	0	0	0	0	0	19	0	miRNA
hsa-miR-503-3p	chrX	1.34E+08	1.34E+08	-	0	0	28	0	0	0	0	0	1	0	miRNA
hsa-miR-503-5p	chrX	1.34E+08	1.34E+08	-	0	0	255	0	29	33	0	0	266	71	miRNA
hsa-miR-504-5p	chrX	1.38E+08	1.38E+08	-	0	0	0	0	24	0	0	0	0	0	miRNA
hsa-miR-505-3p	chrX	1.39E+08	1.39E+08	-	0	70	110	0	116	41	62	0	31	0	miRNA
hsa-miR-505-5p	chrX	1.39E+08	1.39E+08	-	0	0	15	0	0	0	0	0	10	0	miRNA
hsa-miR-5092	chr3	1.25E+08	1.25E+08	-	0	0	0	1	0	0	0	0	0	0	miRNA
hsa-miR-5093	chr16	85339842	85339864	-	0	0	0	0	0	0	0	1	0	0	miRNA
hsa-miR-511-3p	chr10	18134089	18134108	+	0	0	147	0	0	0	0	0	0	0	miRNA
hsa-miR-511-5p	chr10	18134051	18134071	+	0	0	39	56	17	0	0	0	0	0	miRNA
hsa-miR-516b-5p	chr19	54228711	54228732	+	0	0	0	0	5	0	0	0	0	0	miRNA
hsa-miR-5186	chr3	1.51E+08	1.51E+08	-	0	0	0	0	0	0	0	0	18	0	miRNA
hsa-miR-5187-3p	chr1	1.61E+08	1.61E+08	+	0	1	0	0	0	0	0	0	0	0	miRNA
hsa-miR-5188	chr12	1.25E+08	1.25E+08	+	0	0	0	0	0	0	1	0	0	1	miRNA
hsa-miR-5189-5p	chr16	88535351	88535374	+	0	0	67	37	11	0	0	0	30	0	miRNA
hsa-miR-518a-5p	chr19	54242602	54242621	+	0	0	0	0	0	0	0	4	1	0	miRNA
hsa-miR-526a-5p	chr19	54230182	54230203	+	0	0	0	0	0	0	0	0	0	36	miRNA
hsa-miR-539-3p	chr14	1.02E+08	1.02E+08	+	0	0	0	0	13	0	0	0	0	0	miRNA
hsa-miR-539-5p	chr14	1.02E+08	1.02E+08	+	0	0	0	0	22	0	0	0	0	0	miRNA
hsa-miR-542-5p	chrX	1.34E+08	1.34E+08	-	0	0	34	0	6	0	0	0	0	0	miRNA
hsa-miR-545-5p	chrX	73506999	73507020	-	0	0	0	0	14	0	0	0	0	0	miRNA
hsa-miR-548ab	chr3	1.03E+08	1.03E+08	-	0	5	6	0	1	0	3	0	4	1	miRNA
hsa-miR-548ag	chr20	59139623	59139643	+	0	0	0	0	9	0	0	0	0	0	miRNA
hsa-miR-548ah-3p	chr4	77496750	77496771	+	0	0	0	0	11	0	0	0	0	0	miRNA
hsa-miR-548ak	chr10	12172791	12172811	-	0	0	23	0	4	0	0	0	18	43	miRNA
hsa-miR-548am-3p	chrX	16645142	16645163	-	0	16	0	0	0	0	0	0	0	0	miRNA
hsa-miR-548am-5p	chrX	16645178	16645199	-	0	9	236	1	17	39	41	0	75	46	miRNA
hsa-miR-548aq-5p	chr3	1.85E+08	1.85E+08	-	0	0	12	0	0	0	0	0	15	0	miRNA
hsa-miR-548as-5p	chr13	93142416	93142437	+	0	0	37	0	0	0	0	0	0	0	miRNA
hsa-miR-548at-3p	chr17	40646828	40646848	+	0	0	0	0	0	0	0	0	1	0	miRNA
hsa-miR-548at-5p	chr17	40646791	40646812	+	0	0	160	0	0	0	23	0	75	0	miRNA
hsa-miR-548au-3p	chr9	96357156	96357176	+	0	0	7	0	0	0	0	0	0	0	miRNA
hsa-miR-548av-3p	chr18	70520556	70520575	-	0	0	58	0	0	0	0	0	74	23	miRNA
hsa-miR-548aw	chr9	1.36E+08	1.36E+08	+	0	0	23	0	0	0	0	0	0	0	miRNA
hsa-miR-548ax	chrX	11336776	11336797	-	0	0	59	0	1	0	0	0	0	0	miRNA
hsa-miR-548az-5p	chr8	1.2E+08	1.2E+08	+	0	0	24	0	0	0	0	0	0	0	miRNA
hsa-miR-548b-5p	chr6	1.19E+08	1.19E+08	-	0	1	41	1	1	47	1	0	29	45	miRNA
hsa-miR-548c-5p	chr12	65016313	65016334	+	0	9	236	0	17	39	41	0	75	45	miRNA
hsa-miR-548e-3p	chr10	1.13E+08	1.13E+08	+	0	23	0	0	10	0	0	0	11	0	miRNA
hsa-miR-548e-5p	chr10	1.13E+08	1.13E+08	+	0	0	85	0	10	32	3	0	1	0	miRNA
hsa-miR-548f-3p	chr2	2.13E+08	2.13E+08	-	0	0	0	0	0	45	0	0	0	0	miRNA
hsa-miR-548f-5p	chr10	56367686	56367707	-	0	0	1	0	0	0	1	0	0	0	miRNA
hsa-miR-548g-3p	chr4	1.48E+08	1.48E+08	-	0	0	0	0	0	0	0	0	1	0	miRNA
hsa-miR-548i	chr3	1.26E+08	1.26E+08	-	0	0	3	0	1	0	0	0	1	0	miRNA
hsa-miR-548k	chr11	70130092	70130113	+	0	0	0	0	0	0	49	0	28	0	miRNA
hsa-miR-548l	chr11	94199711	94199732	-	0	18	204	0	92	0	46	0	198	47	miRNA
hsa-miR-548n	chr7	34980416	34980437	-	0	28	6	0	10	0	37	0	4	1	miRNA
hsa-miR-548o-3p	chr20	37145250	37145271	+	0	0	58	0	27	0	0	0	74	23	miRNA
hsa-miR-548o-5p	chr20	37145212	37145233	+	0	9	236	0	17	39	41	0	75	45	miRNA
hsa-miR-548q	chr10	12767324	12767345	-	0	0	275	0	28	4	0	0	16	0	miRNA
hsa-miR-548t-5p	chr4	1.74E+08	1.74E+08	+	0	0	0	0	1	0	0	0	0	0	miRNA
hsa-miR-548u	chr6	57254978	57255000	+	0	0	0	0	0	0	0	0	0	6	miRNA
hsa-miR-548w	chr16	26036567	26036589	+	0	0	23	0	4	0	0	0	18	43	miRNA
hsa-miR-548y	chr14	48230264	48230285	-	0	0	0	0	0	0	0	1	0	0	miRNA
hsa-miR-550a-3-5p	chr7	29720405	29720424	-	0	0	1	0	1	0	0	0	0	53	miRNA
hsa-miR-550a-5p	chr7	30329431	30329453	+	0	0	58	1	40	0	0	0	53	94	miRNA
hsa-miR-550b-2-5p	chr7	30329470	30329491	-	0	0	0	0	0	0	0	0	0	50	miRNA
hsa-miR-550b-3p	chr7	30329430	30329449	-	0	0	0	0	0	0	0	0	3	0	miRNA
hsa-miR-551a	chr1	3477274	3477294	-	0	25	0	0	3	0	0	0	0	0	miRNA
hsa-miR-551b-3p	chr3	1.68E+08	1.68E+08	+	0	1	0	0	4	0	0	0	0	0	miRNA
hsa-miR-552-3p	chr1	35135215	35135235	-	0	0	0	0	8	0	0	0	0	0	miRNA
hsa-miR-556-3p	chr1	1.62E+08	1.62E+08	+	0	0	0	0	6	0	0	0	0	0	miRNA
hsa-miR-5583-3p	chr18	37256683	37256704	-	0	0	0	0	7	0	0	0	0	0	miRNA
hsa-miR-5584-5p	chr1	45011165	45011186	+	0	0	0	72	17	0	49	0	0	0	miRNA
hsa-miR-5586-5p	chr14	60113717	60113738	-	0	25	0	0	24	0	0	0	0	0	miRNA
hsa-miR-5588-3p	chr3	1.85E+08	1.85E+08	-	0	0	0	0	3	0	0	0	0	0	miRNA
hsa-miR-5589-5p	chr19	10149030	10149050	+	0	0	0	0	4	0	29	0	0	0	miRNA
hsa-miR-5591-5p	chr4	39413530	39413550	+	0	0	0	34	0	0	0	0	0	0	miRNA
hsa-miR-5680	chr8	1.03E+08	1.03E+08	+	0	0	0	0	0	0	0	0	6	0	miRNA
hsa-miR-5697	chr1	10027448	10027469	+	0	0	0	0	0	0	0	0	15	0	miRNA
hsa-miR-5698	chr1	1.54E+08	1.54E+08	-	0	0	0	0	0	0	46	0	0	0	miRNA
hsa-miR-5699-3p	chr10	687639	687660	-	0	0	0	0	6	0	0	0	0	0	miRNA
hsa-miR-5699-5p	chr10	687688	687709	-	0	16	0	0	0	0	0	0	0	0	miRNA
hsa-miR-5739	chr22	28855913	28855932	+	0	0	0	0	0	0	0	0	0	1	miRNA
hsa-miR-574-3p	chr4	38869713	38869734	+	0	45	53	69	244	0	77	0	0	53	miRNA
hsa-miR-5787	chr3	50264870	50264889	+	0	0	0	0	0	0	0	0	1	0	miRNA
hsa-miR-581	chr5	53247394	53247414	-	0	0	0	0	11	0	0	0	0	0	miRNA
hsa-miR-582-3p	chr5	58999456	58999477	-	0	59	0	66	71	0	0	0	0	23	miRNA
hsa-miR-582-5p	chr5	58999492	58999514	-	0	0	21	0	61	0	36	43	0	38	miRNA
hsa-miR-587	chr6	1.07E+08	1.07E+08	+	0	0	0	0	0	0	0	0	0	1	miRNA
hsa-miR-589-3p	chr7	5535465	5535488	-	0	12	0	0	2	0	0	0	9	0	miRNA

hsa-miR-589-5p	chr7	5535504	5535525	-	0	42	121	0	40	0	41	0	88	43	miRNA
hsa-miR-590-3p	chr7	73605583	73605603	+	0	0	81	45	29	0	0	0	0	97	miRNA
hsa-miR-590-5p	chr7	73605543	73605564	+	0	0	0	0	0	0	0	0	14	0	miRNA
hsa-miR-592	chr7	1.27E+08	1.27E+08	-	0	0	22	0	30	0	0	0	0	0	miRNA
hsa-miR-593-3p	chr7	1.28E+08	1.28E+08	+	0	0	0	0	0	0	0	0	1	0	miRNA
hsa-miR-597-3p	chr8	9599239	9599261	+	0	0	52	0	10	0	0	0	0	19	miRNA
hsa-miR-598-3p	chr8	10892731	10892752	-	0	104	599	0	239	154	130	0	129	240	miRNA
hsa-miR-598-5p	chr8	10892767	10892789	-	0	0	0	0	1	0	0	0	15	0	miRNA
hsa-miR-607	chr10	98588441	98588461	-	0	0	56	0	0	0	0	0	3	0	miRNA
hsa-miR-6076	chr14	50433117	50433137	+	0	0	0	0	12	0	0	0	39	0	miRNA
hsa-miR-6080	chr17	62776914	62776932	+	0	0	0	0	1	0	0	0	0	0	miRNA
hsa-miR-6085	chr15	62635310	62635328	+	0	0	0	0	9	0	0	0	0	0	miRNA
hsa-miR-6086	chrX	13608412	13608431	+	0	0	0	0	8	0	0	0	0	0	miRNA
hsa-miR-610	chr11	28078377	28078397	+	0	0	9	0	0	0	0	0	24	12	miRNA
hsa-miR-6125	chr12	62654199	62654218	+	0	0	0	0	1	0	0	0	1	0	miRNA
hsa-miR-6127	chr1	22959821	22959839	-	0	0	0	1	0	0	0	0	0	0	miRNA
hsa-miR-6130	chr21	24451676	24451694	+	0	0	1	0	4	0	0	0	1	3	miRNA
hsa-miR-616-3p	chr12	57912966	57912987	-	0	5	45	3	45	0	34	0	19	0	miRNA
hsa-miR-616-5p	chr12	57913006	57913027	-	0	12	0	0	29	0	0	0	0	16	miRNA
hsa-miR-618	chr12	81329575	81329597	-	0	0	0	70	31	0	0	0	0	0	miRNA
hsa-miR-624-3p	chr14	31483875	31483895	-	0	0	0	0	0	0	0	0	29	0	miRNA
hsa-miR-624-5p	chr14	31483912	31483933	-	0	0	11	0	4	0	0	0	0	0	miRNA
hsa-miR-627-3p	chr15	42491791	42491810	-	0	19	0	0	0	0	0	0	0	0	miRNA
hsa-miR-627-5p	chr15	42491828	42491849	-	0	5	127	0	46	0	46	0	60	118	miRNA
hsa-miR-628-3p	chr15	55665152	55665172	-	0	0	29	0	5	0	0	0	13	13	miRNA
hsa-miR-628-5p	chr15	55665189	55665210	-	0	0	0	0	9	0	0	0	0	37	miRNA
hsa-miR-629-3p	chr15	70371726	70371747	-	0	0	17	0	0	0	0	0	0	0	miRNA
hsa-miR-636	chr17	74732548	74732570	-	0	0	0	0	3	0	73	0	20	0	miRNA
hsa-miR-638	chr19	10829095	10829119	+	0	12	0	0	0	0	0	0	0	0	miRNA
hsa-miR-639	chr19	14640415	14640437	+	0	0	0	0	7	0	0	0	0	0	miRNA
hsa-miR-641	chr19	40788510	40788533	-	0	27	49	0	35	41	0	0	92	66	miRNA
hsa-miR-642a-3p	chr19	46178236	46178257	+	0	0	0	57	12	0	0	0	0	0	miRNA
hsa-miR-642a-5p	chr19	46178201	46178222	+	0	0	0	0	23	0	0	0	0	51	miRNA
hsa-miR-642b-5p	chr19	46178236	46178257	-	0	0	0	0	0	0	0	0	0	4	miRNA
hsa-miR-643	chr19	52785110	52785131	+	0	0	0	0	1	0	0	0	0	0	miRNA
hsa-miR-645	chr20	49202383	49202401	+	0	0	0	0	0	0	0	0	0	44	miRNA
hsa-miR-6499-5p	chr5	1.51E+08	1.51E+08	-	0	1	0	0	0	0	0	0	0	0	miRNA
hsa-miR-6501-5p	chr21	34922970	34922991	+	0	0	0	0	0	0	0	0	5	0	miRNA
hsa-miR-6503-3p	chr11	59976557	59976577	-	0	0	27	0	8	0	0	48	0	0	miRNA
hsa-miR-6505-3p	chr12	48526630	48526650	+	0	0	0	0	0	0	0	0	0	30	miRNA
hsa-miR-6509-5p	chr7	1.35E+08	1.35E+08	-	0	0	0	53	9	0	0	0	0	50	miRNA
hsa-miR-651-5p	chrX	8095021	8095042	+	0	57	369	0	138	0	42	0	183	71	miRNA
hsa-miR-6511a-3p	chr16	15019837	15019858	+	0	41	35	0	7	0	39	0	35	0	miRNA
hsa-miR-6511b-3p	chr16	2156680	2156702	-	0	4	0	0	0	0	1	0	0	0	miRNA
hsa-miR-6513-3p	chr2	2.19E+08	2.19E+08	-	0	0	24	0	46	1	8	0	0	0	miRNA
hsa-miR-6513-5p	chr2	2.19E+08	2.19E+08	-	0	0	22	0	0	0	0	0	0	0	miRNA
hsa-miR-6514-5p	chr11	62560221	62560243	-	0	15	12	0	0	0	36	0	39	0	miRNA
hsa-miR-6516-3p	chr17	75085551	75085572	+	0	0	0	0	3	0	0	0	0	0	miRNA
hsa-miR-6516-5p	chr17	75085510	75085531	+	0	26	0	0	0	0	0	0	0	0	miRNA
hsa-miR-652-5p	chrX	1.09E+08	1.09E+08	+	0	0	0	0	0	24	0	0	0	0	miRNA
hsa-miR-659-5p	chr22	38243739	38243760	-	0	0	0	0	5	0	0	0	0	0	miRNA
hsa-miR-660-3p	chrX	49777900	49777920	+	0	0	0	0	7	0	56	0	24	0	miRNA
hsa-miR-664a-3p	chr1	2.2E+08	2.2E+08	-	0	14	0	0	28	0	0	0	0	0	miRNA
hsa-miR-664a-5p	chr1	2.2E+08	2.2E+08	-	0	36	140	54	44	1	4	0	44	112	miRNA
hsa-miR-664b-5p	chrX	1.54E+08	1.54E+08	+	0	0	0	0	5	58	0	0	13	0	miRNA
hsa-miR-670-5p	chr11	43581225	43581244	+	0	0	0	0	0	0	0	0	1	0	miRNA
hsa-miR-671-5p	chr7	1.51E+08	1.51E+08	+	0	0	23	0	22	0	0	0	0	13	miRNA
hsa-miR-6716-5p	chr11	1.19E+08	1.19E+08	+	0	0	0	0	0	0	0	0	18	0	miRNA
hsa-miR-6717-5p	chr14	21491514	21491535	-	0	0	0	0	7	0	0	0	0	0	miRNA
hsa-miR-6720-5p	chr6	1390594	1390616	-	0	0	0	0	0	0	0	0	2	0	miRNA
hsa-miR-6721-5p	chr6	32137861	32137883	-	0	0	0	47	30	23	0	0	19	57	miRNA
hsa-miR-6724-5p	chrUn_gl0(	104742	104764	+	0	0	0	0	0	0	0	0	20	0	miRNA
hsa-miR-6726-5p	chr1	1231525	1231545	-	0	0	0	0	0	0	4	0	0	0	miRNA
hsa-miR-6730-5p	chr1	12639024	12639046	-	0	0	0	0	1	0	0	0	37	0	miRNA
hsa-miR-6731-5p	chr1	25245881	25245902	-	0	0	0	0	0	0	0	0	15	0	miRNA
hsa-miR-6732-3p	chr1	37945868	37945890	+	0	0	0	0	6	0	0	0	0	0	miRNA
hsa-miR-6734-5p	chr1	43830359	43830381	-	0	0	84	0	22	24	99	0	0	1	miRNA
hsa-miR-6741-3p	chr1	2.26E+08	2.26E+08	-	0	9	0	0	5	0	39	0	0	46	miRNA
hsa-miR-6742-3p	chr1	2.29E+08	2.29E+08	-	0	0	0	0	1	0	0	0	0	0	miRNA
hsa-miR-6744-5p	chr11	1277840	1277858	+	0	23	0	0	0	0	0	0	0	0	miRNA
hsa-miR-6745	chr11	47201166	47201186	-	0	1	0	1	0	1	0	0	0	0	miRNA
hsa-miR-6747-3p	chr11	62334483	62334503	-	0	0	0	0	21	0	0	0	44	0	miRNA
hsa-miR-6748-3p	chr11	62557337	62557357	+	0	0	0	0	0	42	0	0	0	0	miRNA
hsa-miR-675-3p	chr11	2017998	2018017	-	0	0	0	0	0	0	0	0	0	21	miRNA
hsa-miR-675-5p	chr11	2018030	2018052	-	0	0	13	0	0	0	0	0	0	0	miRNA
hsa-miR-6750-5p	chr11	64665881	64665904	-	0	15	0	0	0	0	1	0	3	0	miRNA
hsa-miR-6751-5p	chr11	64897423	64897445	-	0	0	12	0	1	0	19	0	0	0	miRNA
hsa-miR-6752-5p	chr11	67257721	67257742	+	0	0	1	0	0	0	0	0	0	0	miRNA
hsa-miR-6753-3p	chr11	67812399	67812420	+	0	0	0	0	2	0	0	0	0	0	miRNA
hsa-miR-6754-5p	chr11	71184554	71184575	+	0	0	0	0	0	0	8	0	0	0	miRNA
hsa-miR-6755-5p	chr11	85989380	85989401	+	0	0	0	0	11	0	0	0	0	0	miRNA
hsa-miR-6758-5p	chr12	57906476	57906498	+	0	0	9	0	0	69	0	0	0	0	miRNA
hsa-miR-6761-5p	chr12	1.12E+08	1.12E+08	+	0	0	0	0	5	0	0	0	0	0	miRNA

hsa-miR-6762-3p	chr12	1.14E+08	1.14E+08	+	0	0	0	0	0	0	0	0	14	0	miRNA
hsa-miR-6763-5p	chr12	1.33E+08	1.33E+08	+	0	0	0	0	8	17	0	0	0	47	miRNA
hsa-miR-6764-3p	chr14	1.01E+08	1.01E+08	+	0	0	0	0	3	0	0	0	0	0	miRNA
hsa-miR-6764-5p	chr14	1.01E+08	1.01E+08	+	0	0	0	0	0	0	0	0	0	47	miRNA
hsa-miR-6767-5p	chr16	2495398	2495420	+	0	0	0	0	0	0	0	0	13	0	miRNA
hsa-miR-6769a-5p	chr16	4721324	4721344	+	0	0	20	0	0	0	0	0	0	0	miRNA
hsa-miR-6771-3p	chr16	50326566	50326586	+	0	1	0	0	0	0	0	0	0	0	miRNA
hsa-miR-6777-5p	chr17	17716832	17716854	-	0	0	0	0	5	14	6	0	22	0	miRNA
hsa-miR-6779-5p	chr17	37071237	37071257	+	0	0	16	0	0	0	0	0	0	0	miRNA
hsa-miR-6780a-5p	chr17	40860142	40860164	-	0	0	35	0	5	0	157	0	57	0	miRNA
hsa-miR-6781-5p	chr17	40975936	40975956	-	0	0	0	0	0	0	0	0	27	0	miRNA
hsa-miR-6782-3p	chr17	42285140	42285162	-	0	0	1	0	0	0	0	1	0	0	miRNA
hsa-miR-6783-3p	chr17	43011986	43012007	-	0	0	0	0	9	0	0	0	0	0	miRNA
hsa-miR-6785-5p	chr17	73494634	73494655	+	0	0	14	0	0	0	29	1	27	0	miRNA
hsa-miR-6786-5p	chr17	79660839	79660859	+	0	0	0	0	3	0	0	0	0	0	miRNA
hsa-miR-6790-3p	chr19	6392936	6392956	-	0	0	0	1	0	0	0	0	0	0	miRNA
hsa-miR-6791-3p	chr19	6736723	6736743	-	0	0	0	0	0	39	0	0	0	0	miRNA
hsa-miR-6793-5p	chr19	10939654	10939675	+	0	0	64	0	27	23	0	0	0	0	miRNA
hsa-miR-6800-5p	chr19	50335279	50335299	+	0	0	1	0	0	0	0	0	0	0	miRNA
hsa-miR-6802-5p	chr19	55751320	55751339	-	0	0	53	2	9	0	1	0	0	0	miRNA
hsa-miR-6804-5p	chr19	55742295	55742315	-	0	0	0	0	0	0	0	0	43	0	miRNA
hsa-miR-6805-5p	chr19	55899554	55899575	+	0	13	23	0	2	0	0	0	27	29	miRNA
hsa-miR-6807-3p	chr19	59061721	59061743	+	0	0	0	0	6	0	0	0	0	0	miRNA
hsa-miR-6808-5p	chr1	1275062	1275083	-	0	0	0	0	1	0	0	0	0	0	miRNA
hsa-miR-6810-5p	chr2	2.19E+08	2.19E+08	+	0	0	0	0	25	0	0	0	0	0	miRNA
hsa-miR-6812-5p	chr20	44054155	44054179	+	0	0	0	0	0	0	31	0	0	0	miRNA
hsa-miR-6813-5p	chr20	62708336	62708358	-	0	0	10	0	30	0	0	0	0	0	miRNA
hsa-miR-6815-5p	chr21	46898185	46898207	+	0	0	1	0	0	0	0	0	0	0	miRNA
hsa-miR-6817-5p	chr22	25851618	25851641	+	0	0	0	0	0	0	0	0	1	0	miRNA
hsa-miR-6819-3p	chr22	36682893	36682913	-	0	0	0	0	0	0	0	1	0	0	miRNA
hsa-miR-6819-5p	chr22	36682927	36682948	-	0	0	16	0	6	0	0	0	0	0	miRNA
hsa-miR-6820-3p	chr22	38363610	38363631	+	0	0	0	0	0	1	0	0	0	0	miRNA
hsa-miR-6820-5p	chr22	38363575	38363593	+	0	0	0	0	7	0	0	0	0	0	miRNA
hsa-miR-6823-3p	chr3	48587394	48587414	-	0	0	1	0	0	0	0	0	0	0	miRNA
hsa-miR-6824-5p	chr3	48671105	48671126	-	0	0	0	0	6	0	21	0	13	0	miRNA
hsa-miR-6825-5p	chr3	1.27E+08	1.27E+08	-	0	0	6	0	0	0	0	0	4	0	miRNA
hsa-miR-6826-5p	chr3	1.29E+08	1.29E+08	+	0	0	0	0	9	0	0	0	29	0	miRNA
hsa-miR-6830-5p	chr5	1.32E+08	1.32E+08	-	0	0	0	0	0	0	0	0	1	0	miRNA
hsa-miR-6831-5p	chr5	1.4E+08	1.4E+08	-	0	0	0	0	1	0	0	0	0	0	miRNA
hsa-miR-6832-5p	chr6	31601569	31601591	+	0	0	19	0	20	0	0	0	0	0	miRNA
hsa-miR-6833-5p	chr6	32147598	32147619	+	0	0	87	0	10	0	0	0	17	0	miRNA
hsa-miR-6835-5p	chr6	34208455	34208476	+	0	0	0	0	0	0	0	0	8	0	miRNA
hsa-miR-6837-3p	chr7	44091406	44091428	+	0	0	0	0	4	0	0	0	0	0	miRNA
hsa-miR-6838-5p	chr7	44113006	44113027	-	0	0	0	0	0	0	0	0	2	0	miRNA
hsa-miR-6842-3p	chr8	27290930	27290951	+	0	7	53	0	6	46	0	0	14	74	miRNA
hsa-miR-6843-3p	chr8	27468118	27468138	-	0	0	0	0	4	0	0	0	0	0	miRNA
hsa-miR-6845-5p	chr8	1.45E+08	1.45E+08	-	0	0	0	0	0	36	0	0	0	0	miRNA
hsa-miR-6847-5p	chr8	1.45E+08	1.45E+08	+	0	0	0	0	0	0	36	0	0	0	miRNA
hsa-miR-6849-5p	chr8	1.46E+08	1.46E+08	-	0	0	0	0	0	0	0	0	0	30	miRNA
hsa-miR-6850-5p	chr8	1.46E+08	1.46E+08	-	0	0	0	0	0	0	0	0	21	0	miRNA
hsa-miR-6852-3p	chr9	35710681	35710697	-	0	0	0	0	14	0	0	0	0	1	miRNA
hsa-miR-6858-5p	chrX	1.54E+08	1.54E+08	+	0	0	0	0	0	0	0	0	11	0	miRNA
hsa-miR-6859-5p	chr1	17409	17431	-	0	0	56	0	0	0	0	0	0	24	miRNA
hsa-miR-6860	chr11	66813154	66813175	+	0	0	0	0	4	0	0	0	0	0	miRNA
hsa-miR-6862-5p	chr16	28402346	28402367	-	0	0	1	0	0	0	0	0	0	0	miRNA
hsa-miR-6866-5p	chr17	38318191	38318213	+	0	0	0	0	0	0	0	0	14	0	miRNA
hsa-miR-6867-3p	chr17	38349895	38349915	+	0	0	1	0	0	0	0	0	0	0	miRNA
hsa-miR-6867-5p	chr17	38349854	38349876	+	0	0	28	0	6	0	0	0	0	0	miRNA
hsa-miR-6868-3p	chr17	74094100	74094120	-	0	0	17	0	0	0	0	0	0	0	miRNA
hsa-miR-6872-5p	chr3	50310672	50310691	+	0	0	0	0	0	0	0	0	0	58	miRNA
hsa-miR-6873-5p	chr6	33255040	33255061	-	0	0	1	0	0	0	0	0	0	0	miRNA
hsa-miR-6876-5p	chr8	25202923	25202944	+	0	0	0	0	13	0	0	0	0	0	miRNA
hsa-miR-6879-3p	chr11	64786022	64786042	+	0	0	0	0	0	1	0	0	0	0	miRNA
hsa-miR-6881-5p	chr15	74703747	74703768	-	0	0	0	0	0	0	20	0	0	0	miRNA
hsa-miR-6882-5p	chr15	75133022	75133043	-	0	20	17	64	33	0	44	0	0	39	miRNA
hsa-miR-6883-5p	chr17	8048363	8048384	-	0	0	0	0	0	0	2	0	0	0	miRNA
hsa-miR-6884-5p	chr17	38182636	38182657	-	0	0	57	0	0	0	0	0	22	0	miRNA
hsa-miR-6889-5p	chr22	41649027	41649049	-	0	1	0	0	0	0	0	0	0	0	miRNA
hsa-miR-6891-5p	chr6	31323074	31323092	-	0	0	71	0	0	0	0	0	0	2	miRNA
hsa-miR-6894-3p	chrX	53228071	53228091	-	0	0	0	0	0	0	40	0	0	0	miRNA
hsa-miR-6894-5p	chrX	53228099	53228122	-	0	0	44	0	0	0	1	0	16	0	miRNA
hsa-miR-7-2-3p	chr15	89155127	89155148	+	0	1	0	0	0	0	0	0	0	0	miRNA
hsa-miR-708-3p	chr11	79113076	79113097	-	0	0	58	0	0	0	0	0	0	0	miRNA
hsa-miR-708-5p	chr11	79113121	79113143	-	0	0	0	0	0	0	0	0	0	8	miRNA
hsa-miR-7108-5p	chr19	2434978	2434998	-	0	15	0	0	0	0	0	0	0	0	miRNA
hsa-miR-711	chr3	48616347	48616368	-	0	0	0	0	0	0	0	0	23	0	miRNA
hsa-miR-7155-5p	chr11	64109358	64109376	-	0	0	9	0	6	0	0	0	19	14	miRNA
hsa-miR-7156-5p	chr1	77525828	77525850	+	0	0	0	0	0	0	0	0	0	1	miRNA
hsa-miR-7159-5p	chr6	33866912	33866932	+	0	0	0	0	2	0	0	0	0	0	miRNA
hsa-miR-7161-3p	chr6	1.59E+08	1.59E+08	+	0	0	0	0	0	0	0	0	1	0	miRNA
hsa-miR-718	chrX	1.53E+08	1.53E+08	-	0	0	0	0	0	0	0	0	0	1	miRNA
hsa-miR-744-3p	chr17	11985283	11985304	+	0	0	0	0	36	0	0	0	0	0	miRNA
hsa-miR-758-3p	chr14	1.01E+08	1.01E+08	+	0	0	0	0	18	0	0	0	0	0	miRNA

hsa-miR-762	chr16	30905272	30905293	+	0	0	0	0	0	0	0	0	15	0	miRNA
hsa-miR-766-3p	chrX	1.19E+08	1.19E+08	-	0	0	0	0	19	0	0	0	17	0	miRNA
hsa-miR-769-3p	chr19	46522258	46522280	+	0	21	0	0	0	0	0	0	31	0	miRNA
hsa-miR-7705	chr8	1.02E+08	1.02E+08	-	0	0	0	0	0	0	0	0	0	38	miRNA
hsa-miR-7706	chr15	85923870	85923893	+	0	37	32	0	32	0	75	0	0	0	miRNA
hsa-miR-7843-5p	chr14	72983539	72983560	+	0	1	0	0	0	0	0	0	0	0	miRNA
hsa-miR-7845-5p	chr2	2.08E+08	2.08E+08	+	0	0	0	0	3	0	0	0	0	0	miRNA
hsa-miR-7846-3p	chr1	12227067	12227087	+	0	1	0	0	4	0	0	0	0	0	miRNA
hsa-miR-7847-3p	chr11	1901335	1901355	+	0	0	0	0	0	0	0	0	0	2	miRNA
hsa-miR-7849-3p	chr4	1.47E+08	1.47E+08	+	0	0	0	0	6	0	0	0	0	0	miRNA
hsa-miR-7850-5p	chr19	2630721	2630741	+	0	0	0	0	23	0	0	0	0	0	miRNA
hsa-miR-7854-3p	chr16	81567547	81567567	+	0	0	0	0	29	15	0	0	1	0	miRNA
hsa-miR-7975	chr19	55634593	55634610	-	0	0	0	0	0	0	1	0	0	0	miRNA
hsa-miR-7977	chr3	1.76E+08	1.76E+08	+	0	11	0	0	0	0	0	0	0	0	miRNA
hsa-miR-7978	chr4	21466361	21466381	-	0	0	10	0	0	0	0	0	0	0	miRNA
hsa-miR-8060	chr3	96078850	96078873	+	0	0	0	0	1	0	0	0	0	0	miRNA
hsa-miR-8072	chr12	1.24E+08	1.24E+08	-	0	1	0	0	0	0	46	0	25	0	miRNA
hsa-miR-8077	chr19	42855326	42855347	+	0	0	0	0	0	0	0	0	13	0	miRNA
hsa-miR-8080	chr2	80093677	80093699	-	0	0	0	0	0	0	0	0	16	0	miRNA
hsa-miR-8083	chr1	1.54E+08	1.54E+08	-	0	0	2	0	0	0	0	0	0	0	miRNA
hsa-miR-873-5p	chr9	28888923	28888943	-	0	12	160	0	8	43	0	0	0	33	miRNA
hsa-miR-874-3p	chr5	1.37E+08	1.37E+08	-	0	0	0	0	0	0	0	0	0	45	miRNA
hsa-miR-876-3p	chr9	28863634	28863655	-	0	0	0	0	4	0	0	0	0	0	miRNA
hsa-miR-877-3p	chr6	30552174	30552194	+	0	0	0	0	2	0	0	0	0	0	miRNA
hsa-miR-885-3p	chr3	10436183	10436204	-	0	0	0	0	140	0	10	0	13	0	miRNA
hsa-miR-885-5p	chr3	10436215	10436236	-	0	0	0	0	31	0	0	0	0	0	miRNA
hsa-miR-889-3p	chr14	1.02E+08	1.02E+08	+	0	0	0	154	71	0	0	0	0	0	miRNA
hsa-miR-892a	chrX	1.45E+08	1.45E+08	-	0	0	0	0	0	0	0	0	12	0	miRNA
hsa-miR-92a-1-5p	chr13	92003578	92003600	+	0	0	32	0	24	0	0	0	19	127	miRNA
hsa-miR-92b-5p	chr1	1.55E+08	1.55E+08	+	0	16	76	0	6	1	56	0	94	0	miRNA
hsa-miR-93-3p	chr7	99691400	99691421	-	0	0	29	0	50	3	0	0	49	0	miRNA
hsa-miR-937-3p	chr8	1.45E+08	1.45E+08	-	0	10	0	0	0	37	0	0	20	68	miRNA
hsa-miR-939-5p	chr8	1.46E+08	1.46E+08	-	0	0	79	0	0	14	5	0	33	17	miRNA
hsa-miR-940	chr16	2321807	2321827	+	0	0	26	0	0	0	0	0	0	0	miRNA
hsa-miR-942-3p	chr1	1.18E+08	1.18E+08	+	0	0	0	0	1	0	0	0	1	0	miRNA
hsa-miR-942-5p	chr1	1.18E+08	1.18E+08	+	0	40	388	0	90	31	125	0	218	79	miRNA
hsa-miR-95-3p	chr4	8007039	8007060	-	0	0	27	0	27	0	0	0	17	46	miRNA
hsa-miR-95-5p	chr4	8007074	8007094	-	0	0	10	0	0	0	0	0	0	0	miRNA
hsa-miR-9500	chr2	2.2E+08	2.2E+08	+	0	0	0	0	8	0	0	0	0	0	miRNA
hsa-miR-96-5p	chr7	1.29E+08	1.29E+08	-	0	0	1	0	10	0	61	0	24	0	miRNA
hsa-miR-98-3p	chrX	53583202	53583223	-	0	0	0	0	0	0	0	0	11	0	miRNA
hsa-miR-9903	chr8	99405895	99405916	-	0	0	0	0	6	0	0	0	0	0	miRNA
hsa-miR-9985	chrY	4474181	4474199	+	0	18	18	0	4	0	2	0	0	1	miRNA
hsa-miR-99a-3p	chr21	17911458	17911479	+	0	0	0	0	0	0	0	1	0	0	miRNA
hsa-miR-99a-5p	chr21	17911421	17911442	+	0	0	39	0	0	0	39	0	24	0	miRNA
hsa-miR-99b-3p	chr19	52195909	52195930	+	0	42	32	149	78	51	0	0	24	54	miRNA
hsa-mir-100	chr11	1.22E+08	1.22E+08	-	0	8	346	0	0	1	2	124	1	82	miRNA
hsa-mir-10226	chr17	46110248	46110317	+	0	0	0	0	0	0	1	1	0	0	miRNA
hsa-mir-10393	chr15	45010042	45010091	+	0	0	0	60	26	2	1	0	15	0	miRNA
hsa-mir-10396a	chr21	9826450	9826498	+	0	0	0	35	0	0	0	0	0	1	miRNA
hsa-mir-10397	chr5	10402476	10402543	+	0	1	0	0	0	0	0	0	0	0	miRNA
hsa-mir-10399	chr7	1.39E+08	1.39E+08	-	0	32	68	8	169	40	72	69	28	52	miRNA
hsa-mir-103b-1	chr5	1.68E+08	1.68E+08	+	0	0	0	0	0	0	0	0	2	0	miRNA
hsa-mir-11400	chr7	1.42E+08	1.42E+08	-	0	171	49	53	297	38	22	0	19	82	miRNA
hsa-mir-11401	chr16	87887502	87887570	+	0	0	35	0	0	0	0	0	195	0	miRNA
hsa-mir-1179	chr15	89151338	89151428	+	0	0	30	57	15	0	0	0	0	0	miRNA
hsa-mir-1181	chr19	10514134	10514214	-	0	31	2	7	103	49	39	0	4	49	miRNA
hsa-mir-1185-1	chr14	1.02E+08	1.02E+08	+	0	14	0	57	37	0	0	0	10	0	miRNA
hsa-mir-1185-2	chr14	1.02E+08	1.02E+08	+	0	14	0	0	18	0	0	0	1	0	miRNA
hsa-mir-1199	chr19	14184173	14184291	+	0	0	0	0	0	0	1	0	0	0	miRNA
hsa-mir-1202	chr6	1.56E+08	1.56E+08	+	0	0	0	0	8	0	0	0	0	0	miRNA
hsa-mir-1204	chr8	1.29E+08	1.29E+08	+	0	1	0	0	0	0	0	0	0	0	miRNA
hsa-mir-1207	chr8	1.29E+08	1.29E+08	+	0	23	0	0	0	0	0	0	0	0	miRNA
hsa-mir-12114	chr22	50845871	50845953	+	0	0	0	0	0	0	0	0	24	0	miRNA
hsa-mir-12119	chr7	99006795	99006887	+	0	0	0	0	0	0	0	0	2	2	miRNA
hsa-mir-12120	chrY	15591057	15591146	+	0	0	0	0	0	56	22	0	0	0	miRNA
hsa-mir-12121	chr14	1.05E+08	1.05E+08	+	0	0	0	0	0	0	1	0	0	0	miRNA
hsa-mir-12124	chr3	1.29E+08	1.29E+08	+	0	0	0	0	5	0	0	0	0	0	miRNA
hsa-mir-12125	chr5	1.68E+08	1.68E+08	+	0	1	1	1	0	0	1	0	0	1	miRNA
hsa-mir-12126	chr9	1.33E+08	1.33E+08	+	0	2	0	0	0	0	0	0	0	0	miRNA
hsa-mir-12135	chr15	73592251	73592320	+	0	0	0	0	6	0	0	0	0	0	miRNA
hsa-mir-1224	chr3	1.84E+08	1.84E+08	+	0	24	102	0	9	0	37	0	43	0	miRNA
hsa-mir-1226	chr3	47891045	47891119	+	0	0	1	0	6	0	0	0	21	0	miRNA
hsa-mir-1229	chr5	1.79E+08	1.79E+08	-	0	0	0	0	12	0	0	1	0	0	miRNA
hsa-mir-122b	chr18	56118312	56118384	-	0	0	1	0	0	0	0	0	0	1	miRNA
hsa-mir-1236	chr6	31924616	31924717	-	0	0	0	0	0	0	22	0	40	0	miRNA
hsa-mir-1237	chr11	64136074	64136175	+	0	1	0	0	0	0	0	0	0	0	miRNA
hsa-mir-124-1	chr8	9760898	9760982	-	0	0	0	0	0	0	0	0	0	3	miRNA
hsa-mir-124-2	chr8	65291706	65291814	+	0	0	0	0	0	0	0	0	0	3	miRNA
hsa-mir-124-3	chr20	61809852	61809938	+	0	0	0	0	0	0	0	0	0	3	miRNA
hsa-mir-1245b	chr2	1.9E+08	1.9E+08	-	0	0	0	0	1	0	0	0	0	0	miRNA
hsa-mir-1247	chr14	1.02E+08	1.02E+08	-	0	0	0	0	0	0	0	0	18	0	miRNA
hsa-mir-1248	chr3	1.87E+08	1.87E+08	+	0	0	0	0	10	35	0	0	0	0	miRNA

hsa-mir-1250	chr17	79106996	79107108	-	0	13	0	0	6	0	0	0	0	1	miRNA
hsa-mir-1255a	chr4	1.02E+08	1.02E+08	-	0	1	44	0	0	0	1	0	1	0	miRNA
hsa-mir-1258	chr2	1.81E+08	1.81E+08	-	0	0	0	0	7	0	0	0	0	0	miRNA
hsa-mir-125b-2	chr21	17962557	17962645	+	0	40	188	0	10	14	0	118	0	0	miRNA
hsa-mir-1260b	chr11	96074602	96074690	+	0	7	0	0	172	1	0	0	1	6	miRNA
hsa-mir-1262	chr1	68649201	68649293	-	0	20	0	0	13	0	0	0	7	0	miRNA
hsa-mir-1268a	chr15	22513229	22513280	-	0	0	0	0	0	0	0	0	1	0	miRNA
hsa-mir-1268b	chr17	78072627	78072676	+	0	0	0	0	0	0	0	0	1	0	miRNA
hsa-mir-1272	chr15	65054586	65054714	-	0	0	0	0	0	0	2	0	0	0	miRNA
hsa-mir-1273c	chr6	1.55E+08	1.55E+08	+	0	0	5	0	1	0	0	0	0	0	miRNA
hsa-mir-1275	chr6	33967749	33967828	-	0	0	31	2	16	26	24	0	0	0	miRNA
hsa-mir-1277	chrX	1.18E+08	1.18E+08	+	0	0	0	1	0	0	0	0	0	0	miRNA
hsa-mir-1285-1	chr7	91833329	91833412	-	0	23	157	0	0	0	0	0	91	0	miRNA
hsa-mir-1285-2	chr2	70480050	70480137	-	0	23	143	0	0	0	0	0	91	0	miRNA
hsa-mir-1288	chr17	16185328	16185402	+	0	18	0	0	0	0	0	0	0	0	miRNA
hsa-mir-129-1	chr7	1.28E+08	1.28E+08	+	0	0	1	0	9	0	2	0	0	0	miRNA
hsa-mir-129-2	chr11	43602944	43603033	+	0	0	1	0	8	42	2	0	0	0	miRNA
hsa-mir-1295a	chr1	1.71E+08	1.71E+08	-	0	0	0	0	14	0	0	0	0	0	miRNA
hsa-mir-1295b	chr1	1.71E+08	1.71E+08	+	0	0	0	0	0	0	1	0	0	0	miRNA
hsa-mir-1296	chr10	65132717	65132808	-	0	0	0	0	6	0	0	0	0	0	miRNA
hsa-mir-1299	chr9	69002239	69002321	-	0	0	240	0	0	0	153	0	61	0	miRNA
hsa-mir-1301	chr2	25551509	25551590	-	0	0	88	0	12	0	41	0	4	20	miRNA
hsa-mir-1303	chr5	1.54E+08	1.54E+08	+	0	0	26	0	5	24	16	0	27	14	miRNA
hsa-mir-1304	chr11	93466840	93466930	-	0	49	118	138	166	0	0	0	77	111	miRNA
hsa-mir-1306	chr22	20073581	20073665	+	0	20	22	0	53	0	0	0	57	70	miRNA
hsa-mir-132	chr17	1953202	1953302	-	0	53	180	35	115	25	31	3	97	60	miRNA
hsa-mir-1321	chrX	85090785	85090863	+	0	0	0	0	0	0	0	2	0	0	miRNA
hsa-mir-133a-1	chr18	19405659	19405746	-	0	36	63	1	59	89	0	0	46	103	miRNA
hsa-mir-133a-2	chr20	61162119	61162220	+	0	36	63	0	59	89	0	0	46	103	miRNA
hsa-mir-133b	chr6	52013721	52013839	+	0	0	0	0	0	2	0	0	2	4	miRNA
hsa-mir-135b	chr1	2.05E+08	2.05E+08	-	0	0	0	0	1	0	0	0	0	0	miRNA
hsa-mir-136	chr14	1.01E+08	1.01E+08	+	0	24	10	93	38	0	0	0	0	29	miRNA
hsa-mir-137	chr1	98511626	98511727	-	0	0	0	0	9	0	0	1	0	0	miRNA
hsa-mir-138-1	chr3	44155704	44155802	+	0	0	0	0	0	0	0	0	3	0	miRNA
hsa-mir-138-2	chr16	56892430	56892513	+	0	1	0	0	0	0	0	0	0	0	miRNA
hsa-mir-139	chr11	72326107	72326174	-	0	70	67	41	348	92	57	0	12	196	miRNA
hsa-mir-141	chr12	7073260	7073354	+	0	0	119	0	10	0	0	38	0	0	miRNA
hsa-mir-145	chr5	1.49E+08	1.49E+08	+	0	0	308	0	15	29	70	0	34	23	miRNA
hsa-mir-1469	chr15	96876490	96876536	+	0	3	0	0	0	0	0	0	0	0	miRNA
hsa-mir-147b	chr15	45725248	45725327	+	0	0	32	0	6	0	0	0	0	0	miRNA
hsa-mir-149	chr2	2.41E+08	2.41E+08	+	0	0	0	0	0	0	1	0	0	0	miRNA
hsa-mir-153-1	chr2	2.2E+08	2.2E+08	-	0	0	0	0	0	0	0	0	0	23	miRNA
hsa-mir-153-2	chr7	1.57E+08	1.57E+08	-	0	0	0	0	0	0	0	0	0	23	miRNA
hsa-mir-1538	chr16	69599711	69599771	-	0	4	5	0	5	0	0	0	25	1	miRNA
hsa-mir-1539	chr18	47013743	47013792	+	0	0	0	0	1	0	1	0	0	0	miRNA
hsa-mir-154	chr14	1.02E+08	1.02E+08	+	0	0	9	75	46	0	0	0	26	0	miRNA
hsa-mir-184	chr15	79502130	79502213	+	0	1	0	0	7	0	0	0	0	0	miRNA
hsa-mir-188	chrX	49768109	49768194	+	0	0	33	0	11	0	0	0	0	0	miRNA
hsa-mir-18a	chr13	92003005	92003075	+	0	268	527	23	428	77	208	0	599	392	miRNA
hsa-mir-18b	chrX	1.33E+08	1.33E+08	-	0	92	380	0	54	1	122	0	362	164	miRNA
hsa-mir-190a	chr15	63116156	63116240	+	0	61	0	0	13	0	21	0	19	15	miRNA
hsa-mir-1910	chr16	85775227	85775306	-	0	0	25	0	0	0	0	0	0	0	miRNA
hsa-mir-193b	chr16	14397824	14397906	+	0	0	196	32	54	12	41	0	0	36	miRNA
hsa-mir-194-1	chr1	2.2E+08	2.2E+08	-	0	20	165	17	98	0	0	0	18	0	miRNA
hsa-mir-194-2	chr11	64658827	64658911	-	0	45	195	17	126	0	1	0	18	40	miRNA
hsa-mir-195	chr17	6920934	6921020	-	0	59	58	0	7	2	2	46	9	4	miRNA
hsa-mir-196a-1	chr17	46709852	46709921	-	0	0	55	0	25	0	38	0	0	0	miRNA
hsa-mir-196a-2	chr12	54385522	54385631	+	0	24	55	0	37	0	73	0	0	0	miRNA
hsa-mir-196b	chr7	27209099	27209182	-	0	101	792	59	207	49	99	0	190	274	miRNA
hsa-mir-197	chr1	1.1E+08	1.1E+08	+	0	35	133	0	142	21	101	0	71	176	miRNA
hsa-mir-1972-1	chr16	15104178	15104254	-	0	8	0	0	44	30	0	0	0	1	miRNA
hsa-mir-1972-2	chr16	70064249	70064325	+	0	0	0	0	0	0	0	0	0	1	miRNA
hsa-mir-200b	chr1	1102484	1102578	+	0	22	38	64	23	0	0	0	0	0	miRNA
hsa-mir-200c	chr12	7072862	7072929	+	0	43	58	99	218	122	59	0	14	66	miRNA
hsa-mir-202	chr10	1.35E+08	1.35E+08	-	0	0	0	0	0	3	0	0	7	0	miRNA
hsa-mir-205	chr1	2.1E+08	2.1E+08	+	0	19	129	61	23	0	38	1	28	62	miRNA
hsa-mir-208b	chr14	23887196	23887272	-	0	0	159	0	0	0	47	0	0	0	miRNA
hsa-mir-2110	chr10	1.16E+08	1.16E+08	-	0	22	106	81	77	26	68	0	91	44	miRNA
hsa-mir-2116	chr15	59463382	59463461	-	0	0	0	0	0	0	0	0	0	19	miRNA
hsa-mir-214	chr1	1.72E+08	1.72E+08	-	0	0	23	0	8	0	0	0	0	0	miRNA
hsa-mir-215	chr1	2.2E+08	2.2E+08	-	0	2	68	5	18	46	10	2	5	6	miRNA
hsa-mir-216b	chr2	56227849	56227930	-	0	1	18	1	0	1	18	2	0	2	miRNA
hsa-mir-217	chr2	56210102	56210211	-	0	0	57	0	0	0	0	44	0	60	miRNA
hsa-mir-219a-1	chr6	33175612	33175721	+	0	0	0	0	18	0	0	0	0	0	miRNA
hsa-mir-219b	chr9	1.31E+08	1.31E+08	+	0	1	0	0	4	1	15	1	0	0	miRNA
hsa-mir-224	chrX	1.51E+08	1.51E+08	-	0	0	15	0	31	0	0	0	0	0	miRNA
hsa-mir-2276	chr13	24736555	24736643	+	0	0	0	0	0	0	49	0	0	0	miRNA
hsa-mir-2277	chr5	92956402	92956494	-	0	10	62	0	6	0	0	0	0	24	miRNA
hsa-mir-2355	chr2	2.08E+08	2.08E+08	-	0	0	0	0	26	0	0	0	15	0	miRNA
hsa-mir-2392	chr14	1.01E+08	1.01E+08	+	0	0	0	0	1	0	0	0	0	1	miRNA
hsa-mir-2467	chr2	2.4E+08	2.4E+08	-	0	0	0	0	0	0	0	95	0	0	miRNA
hsa-mir-2682	chr1	98510798	98510907	-	0	0	0	2	0	0	0	0	0	0	miRNA
hsa-mir-299	chr14	1.01E+08	1.01E+08	+	0	0	0	0	28	0	0	0	22	0	miRNA

hsa-mir-29b-1	chr7	1.31E+08	1.31E+08	-	0	0	21	2	22	0	40	0	17	19	miRNA
hsa-mir-301b	chr22	22007270	22007347	+	0	0	0	0	10	59	0	0	0	0	miRNA
hsa-mir-302e	chr11	7255997	7256068	+	0	0	0	0	0	28	0	0	24	130	miRNA
hsa-mir-3064	chr17	62496892	62496957	-	0	0	0	0	0	0	0	0	30	0	miRNA
hsa-mir-3065	chr17	79099677	79099755	+	0	0	23	0	10	58	0	0	0	0	miRNA
hsa-mir-3074	chr9	97848296	97848376	-	0	0	0	0	8	2	0	0	13	37	miRNA
hsa-mir-3085	chr10	99635570	99635654	-	0	1	0	0	0	0	0	0	0	0	miRNA
hsa-mir-31	chr9	21512114	21512184	-	0	26	25	0	37	25	0	0	3	78	miRNA
hsa-mir-3115	chr1	23370798	23370865	+	0	0	0	0	24	0	0	0	0	0	miRNA
hsa-mir-3117	chr1	67094123	67094200	+	0	0	0	0	6	0	0	0	0	0	miRNA
hsa-mir-3120	chr1	1.72E+08	1.72E+08	+	0	50	57	0	117	0	0	0	0	15	miRNA
hsa-mir-3122	chr1	2.12E+08	2.12E+08	+	0	0	0	0	0	0	0	0	6	0	miRNA
hsa-mir-3127	chr2	97464015	97464090	+	0	0	95	0	7	0	0	0	95	0	miRNA
hsa-mir-3128	chr2	1.78E+08	1.78E+08	-	0	0	21	0	0	0	0	0	0	0	miRNA
hsa-mir-3129	chr2	1.9E+08	1.9E+08	-	0	0	0	0	0	0	37	0	0	0	miRNA
hsa-mir-3130-1	chr2	2.08E+08	2.08E+08	-	0	0	14	0	1	0	0	0	74	42	miRNA
hsa-mir-3130-2	chr2	2.08E+08	2.08E+08	+	0	0	14	0	5	0	0	0	74	42	miRNA
hsa-mir-3131	chr2	2.2E+08	2.2E+08	-	0	0	38	0	0	0	0	0	0	0	miRNA
hsa-mir-3135a	chr3	20179057	20179133	+	0	0	33	0	0	0	0	0	0	0	miRNA
hsa-mir-3135b	chr6	32717689	32717756	-	0	1	0	0	0	0	0	1	0	1	miRNA
hsa-mir-3136	chr3	69098109	69098186	-	0	0	36	0	6	0	0	0	21	0	miRNA
hsa-mir-3139	chr4	1.44E+08	1.44E+08	+	0	0	0	0	0	0	29	0	0	0	miRNA
hsa-mir-3143	chr6	27115405	27115467	+	0	20	96	0	0	0	1	24	93	0	miRNA
hsa-mir-3146	chr7	19744981	19745059	-	0	0	0	0	11	0	0	0	0	0	miRNA
hsa-mir-3147	chr7	57472731	57472796	+	0	26	0	0	0	0	0	0	0	0	miRNA
hsa-mir-3150a	chr8	96085142	96085221	+	0	0	0	0	14	0	0	0	17	0	miRNA
hsa-mir-3150b	chr8	96085139	96085224	-	0	0	34	0	5	0	0	0	0	0	miRNA
hsa-mir-3153	chr9	91927140	91927221	+	0	0	0	0	0	0	0	0	9	1	miRNA
hsa-mir-3154	chr9	1.31E+08	1.31E+08	-	0	0	64	0	8	0	0	0	0	94	miRNA
hsa-mir-3155a	chr10	6194159	6194240	+	0	0	18	0	0	0	0	0	0	0	miRNA
hsa-mir-3157	chr10	97824072	97824156	-	0	0	12	0	11	0	0	0	12	0	miRNA
hsa-mir-3158-1	chr10	1.03E+08	1.03E+08	+	0	0	128	0	19	0	0	0	19	122	miRNA
hsa-mir-3158-2	chr10	1.03E+08	1.03E+08	-	0	0	128	0	19	0	0	0	19	122	miRNA
hsa-mir-3160-1	chr11	46473355	46473439	-	0	0	0	0	11	0	0	0	0	0	miRNA
hsa-mir-3160-2	chr11	46473357	46473437	+	0	0	0	0	11	0	0	0	0	0	miRNA
hsa-mir-3164	chr11	68850644	68850726	+	0	0	71	0	12	0	0	0	30	0	miRNA
hsa-mir-3165	chr11	71783274	71783348	-	0	0	0	0	9	0	0	0	19	0	miRNA
hsa-mir-3170	chr13	98860778	98860854	+	0	0	0	0	0	0	0	0	1	0	miRNA
hsa-mir-3173	chr14	95604256	95604323	-	0	0	53	0	24	0	1	0	25	0	miRNA
hsa-mir-3174	chr15	90549987	90550073	+	0	0	0	0	22	0	0	0	0	0	miRNA
hsa-mir-3175	chr15	93447629	93447705	+	0	0	0	0	0	0	1	0	0	0	miRNA
hsa-mir-3176	chr16	593277	593366	+	0	0	0	0	4	0	0	0	0	0	miRNA
hsa-mir-3178	chr16	2581923	2582006	-	0	1	1	0	0	0	0	0	0	0	miRNA
hsa-mir-3179-1	chr16	14995365	14995448	+	0	0	0	0	3	0	0	0	0	0	miRNA
hsa-mir-3179-2	chr16	16394016	16394099	+	0	0	0	0	3	0	0	0	0	0	miRNA
hsa-mir-3179-3	chr16	18505751	18505834	-	0	0	0	0	3	0	0	0	0	0	miRNA
hsa-mir-3179-4	chr16	14995365	14995448	+	0	0	0	0	3	0	0	0	0	0	miRNA
hsa-mir-3182	chr16	83541951	83542013	+	0	0	0	1	3	1	0	0	1	0	miRNA
hsa-mir-3185	chr17	46801770	46801837	-	0	1	0	0	0	0	0	0	0	0	miRNA
hsa-mir-3186	chr17	79418130	79418214	-	0	0	0	0	8	0	0	0	0	0	miRNA
hsa-mir-3187	chr19	813584	813653	+	0	0	35	0	8	0	0	0	49	0	miRNA
hsa-mir-3190	chr19	47730199	47730278	+	0	1	0	0	11	0	0	0	0	0	miRNA
hsa-mir-3191	chr19	47730201	47730276	-	0	6	0	0	0	7	5	0	21	0	miRNA
hsa-mir-3192	chr20	18451259	18451335	+	0	20	16	0	26	0	0	0	0	0	miRNA
hsa-mir-3193	chr20	30194989	30195043	+	0	0	0	0	0	1	0	0	0	0	miRNA
hsa-mir-3194	chr20	50069442	50069514	-	0	0	0	0	0	0	0	0	11	0	miRNA
hsa-mir-3197	chr21	42539484	42539556	+	0	0	0	0	0	0	1	0	0	0	miRNA
hsa-mir-3198-1	chr22	18246946	18247025	-	0	0	0	1	15	0	0	0	0	0	miRNA
hsa-mir-3198-2	chr12	54625181	54625260	-	0	0	0	0	15	0	0	0	0	0	miRNA
hsa-mir-3199-2	chr22	28316514	28316599	+	0	0	19	0	0	0	0	0	0	0	miRNA
hsa-mir-32	chr9	1.12E+08	1.12E+08	-	0	71	586	0	65	0	129	0	132	187	miRNA
hsa-mir-3200	chr22	31127544	31127628	+	0	0	47	1	23	0	166	0	123	0	miRNA
hsa-mir-3202-1	chrX	1.53E+08	1.53E+08	+	0	0	0	16	0	2	23	0	0	0	miRNA
hsa-mir-3202-2	chrX	1.53E+08	1.53E+08	-	0	0	0	16	0	0	23	0	0	0	miRNA
hsa-mir-323b	chr14	1.02E+08	1.02E+08	+	0	88	74	218	314	35	0	0	41	0	miRNA
hsa-mir-326	chr11	75046136	75046230	-	0	0	0	0	55	0	32	0	0	43	miRNA
hsa-mir-329-1	chr14	1.01E+08	1.01E+08	+	0	0	0	61	4	0	0	0	0	0	miRNA
hsa-mir-329-2	chr14	1.01E+08	1.01E+08	+	0	0	0	61	4	0	0	0	0	0	miRNA
hsa-mir-330	chr19	46142252	46142345	-	0	51	83	0	121	45	0	0	0	76	miRNA
hsa-mir-331	chr12	95702196	95702289	+	0	0	40	0	8	32	0	0	31	0	miRNA
hsa-mir-337	chr14	1.01E+08	1.01E+08	+	0	0	0	61	25	41	0	0	0	1	miRNA
hsa-mir-346	chr10	88024451	88024545	-	0	1	0	0	0	0	0	0	0	0	miRNA
hsa-mir-34c	chr11	1.11E+08	1.11E+08	+	0	73	73	55	23	0	0	10	34	0	miRNA
hsa-mir-3610	chr8	1.18E+08	1.18E+08	-	0	19	0	0	0	0	0	0	0	0	miRNA
hsa-mir-3613	chr13	50570551	50570637	-	0	0	0	0	0	0	16	0	0	0	miRNA
hsa-mir-3614	chr17	54968631	54968716	-	0	0	0	0	19	0	0	0	0	131	miRNA
hsa-mir-3618	chr22	20073269	20073356	+	0	0	0	0	1	0	0	0	0	0	miRNA
hsa-mir-3619	chr22	46486924	46487006	+	0	0	0	0	0	0	44	0	0	0	miRNA
hsa-mir-362	chrX	49773572	49773636	+	0	0	23	0	9	0	30	0	3	61	miRNA
hsa-mir-3621	chr9	1.4E+08	1.4E+08	-	0	4	0	0	0	0	0	0	0	0	miRNA
hsa-mir-363	chrX	1.33E+08	1.33E+08	-	0	9	1070	0	114	0	440	47	773	189	miRNA
hsa-mir-3649	chr12	1769481	1769546	-	0	0	0	0	0	0	0	0	1	0	miRNA
hsa-mir-3652	chr12	1.04E+08	1.04E+08	+	0	0	0	1	13	34	0	0	0	0	miRNA

hsa-mir-365a	chr16	14403142	14403228	+	0	0	10	0	12	30	0	0	0	0	miRNA
hsa-mir-365b	chr17	29902430	29902540	+	0	0	0	0	22	0	0	40	0	0	miRNA
hsa-mir-3661	chr5	1.34E+08	1.34E+08	+	0	0	0	0	0	0	0	1	0	0	miRNA
hsa-mir-3663	chr10	1.19E+08	1.19E+08	-	0	0	0	0	0	0	1	0	0	0	miRNA
hsa-mir-3667	chr22	49937041	49937114	-	0	0	0	0	2	0	0	0	0	0	miRNA
hsa-mir-3674	chr8	1749291	1749358	+	0	0	0	1	0	0	0	0	0	0	miRNA
hsa-mir-3675	chr1	17185444	17185516	-	0	0	1	47	1	0	0	0	0	0	miRNA
hsa-mir-3679	chr2	1.35E+08	1.35E+08	+	0	0	309	0	50	20	0	0	0	37	miRNA
hsa-mir-3680-1	chr16	21517370	21517456	-	0	0	0	0	1	0	0	0	0	0	miRNA
hsa-mir-3680-2	chr16	29610500	29610586	-	0	0	0	0	1	0	0	0	0	0	miRNA
hsa-mir-3682	chr2	54076259	54076342	-	0	0	120	0	25	0	39	0	71	0	miRNA
hsa-mir-3688-1	chr4	1.6E+08	1.6E+08	-	0	51	133	0	30	0	0	0	40	54	miRNA
hsa-mir-3688-2	chr4	1.6E+08	1.6E+08	+	0	51	133	0	30	0	0	0	40	55	miRNA
hsa-mir-369	chr14	1.02E+08	1.02E+08	+	0	15	165	67	177	0	36	1	0	1	miRNA
hsa-mir-3690-1	chrX	1412811	1412885	+	0	0	0	0	1	0	0	0	0	0	miRNA
hsa-mir-3690-2	chrY	1362811	1362885	+	0	0	0	0	1	0	0	0	0	0	miRNA
hsa-mir-371a	chr19	54290929	54290995	+	0	0	0	0	4	0	0	0	0	0	miRNA
hsa-mir-371b	chr19	54290931	54290996	-	0	0	0	0	0	0	0	0	1	0	miRNA
hsa-mir-372	chr19	54291144	54291210	+	0	0	0	0	5	0	0	0	0	0	miRNA
hsa-mir-373	chr19	54291959	54292027	+	0	0	38	0	0	1	0	68	0	0	miRNA
hsa-mir-374b	chrX	73438382	73438453	-	0	69	43	0	142	37	41	0	7	0	miRNA
hsa-mir-374c	chrX	73438384	73438453	+	0	0	1	0	1	0	1	0	0	0	miRNA
hsa-mir-376a-1	chr14	1.02E+08	1.02E+08	+	0	16	0	0	34	36	0	0	0	73	miRNA
hsa-mir-376a-2	chr14	1.02E+08	1.02E+08	+	0	16	0	0	34	36	0	0	0	73	miRNA
hsa-mir-376c	chr14	1.02E+08	1.02E+08	+	0	0	0	0	11	0	0	0	0	0	miRNA
hsa-mir-378f	chr1	24255560	24255637	+	0	1	4	1	7	0	0	0	2	2	miRNA
hsa-mir-380	chr14	1.01E+08	1.01E+08	+	0	0	0	0	0	19	0	0	0	0	miRNA
hsa-mir-381	chr14	1.02E+08	1.02E+08	+	0	45	24	110	291	99	0	86	15	104	miRNA
hsa-mir-3911	chr9	1.3E+08	1.3E+08	-	0	0	72	0	0	0	0	0	0	0	miRNA
hsa-mir-3912	chr5	1.71E+08	1.71E+08	-	0	0	55	0	0	0	0	0	0	48	miRNA
hsa-mir-3913-1	chr12	69978502	69978603	-	0	0	0	0	0	22	0	0	28	34	miRNA
hsa-mir-3913-2	chr12	69978503	69978602	+	0	0	0	0	0	22	0	0	28	0	miRNA
hsa-mir-3914-1	chr7	70772658	70772756	-	0	0	2	0	0	0	0	1	0	0	miRNA
hsa-mir-3914-2	chr7	70772660	70772754	+	0	0	0	0	0	1	0	0	0	0	miRNA
hsa-mir-3917	chr1	26232853	26232945	-	0	0	0	0	1	0	2	0	0	0	miRNA
hsa-mir-3918	chr6	1.59E+08	1.59E+08	-	0	0	0	0	3	0	0	0	0	0	miRNA
hsa-mir-3919	chr3	1.59E+08	1.59E+08	+	0	0	0	0	1	0	0	0	1	0	miRNA
hsa-mir-3921	chr3	99683158	99683242	-	0	0	0	0	1	0	0	0	0	0	miRNA
hsa-mir-3922	chr12	1.05E+08	1.05E+08	+	0	0	22	0	22	37	28	0	0	93	miRNA
hsa-mir-3923	chr3	79557037	79557119	+	0	1	0	0	0	0	0	0	0	0	miRNA
hsa-mir-3925	chr6	36590213	36590289	-	0	0	11	0	0	0	0	0	0	0	miRNA
hsa-mir-3928	chr22	31556048	31556105	-	0	0	59	0	38	6	0	0	0	0	miRNA
hsa-mir-3934	chr6	33665905	33666011	+	0	0	11	0	0	0	0	0	18	0	miRNA
hsa-mir-3935	chr16	56279432	56279535	+	0	0	0	0	1	0	0	0	0	0	miRNA
hsa-mir-3936	chr5	1.32E+08	1.32E+08	-	0	0	0	0	7	0	0	0	23	0	miRNA
hsa-mir-3937	chrX	39520470	39520575	+	0	0	0	0	4	2	0	0	0	0	miRNA
hsa-mir-3938	chr3	55886520	55886622	-	0	0	0	0	0	0	1	0	0	0	miRNA
hsa-mir-3939	chr6	1.67E+08	1.67E+08	-	0	0	0	0	5	0	0	0	0	0	miRNA
hsa-mir-3945	chr4	1.86E+08	1.86E+08	-	0	0	0	0	10	0	0	0	0	0	miRNA
hsa-mir-3960	chr9	1.31E+08	1.31E+08	+	0	1	0	0	0	1	0	0	0	2	miRNA
hsa-mir-3975	chr18	33171701	33171770	+	0	0	0	0	1	0	0	0	0	0	miRNA
hsa-mir-411	chr14	1.01E+08	1.01E+08	+	0	17	25	253	241	143	0	0	20	0	miRNA
hsa-mir-412	chr14	1.02E+08	1.02E+08	+	0	0	108	168	19	1	0	0	0	0	miRNA
hsa-mir-422a	chr15	64163129	64163218	-	0	1	5	0	1	0	0	0	1	2	miRNA
hsa-mir-4267	chr2	1.11E+08	1.11E+08	-	0	0	0	0	0	0	40	0	0	0	miRNA
hsa-mir-4270	chr3	15537746	15537815	-	0	0	0	0	1	0	0	0	0	0	miRNA
hsa-mir-4271	chr3	49311553	49311619	+	0	0	0	26	0	0	0	0	0	0	miRNA
hsa-mir-4278	chr5	6827966	6828034	-	0	0	1	0	0	0	0	0	0	0	miRNA
hsa-mir-4282	chr6	73677410	73677476	-	0	0	0	0	8	0	0	0	0	0	miRNA
hsa-mir-4284	chr7	73125647	73125727	+	0	0	0	0	0	0	0	0	1	0	miRNA
hsa-mir-4289	chr9	91360751	91360820	-	0	1	0	0	0	0	0	0	0	0	miRNA
hsa-mir-429	chr1	1104385	1104467	+	0	23	7	0	19	0	0	0	0	0	miRNA
hsa-mir-4294	chr10	50193557	50193632	-	0	0	0	1	0	0	0	0	0	0	miRNA
hsa-mir-4297	chr10	1.32E+08	1.32E+08	-	0	1	0	0	0	0	0	0	0	0	miRNA
hsa-mir-4307	chr14	27377848	27377931	+	0	0	0	0	0	0	1	0	0	0	miRNA
hsa-mir-4311	chr15	66332571	66332670	+	0	0	0	0	17	0	0	0	0	1	miRNA
hsa-mir-4313	chr15	76054556	76054656	-	0	0	0	0	1	0	0	0	0	0	miRNA
hsa-mir-4314	chr17	7991374	7991465	+	0	0	0	1	0	0	0	0	0	0	miRNA
hsa-mir-4320	chr18	47652869	47652933	-	0	0	0	0	1	0	0	0	0	0	miRNA
hsa-mir-4325	chr20	55896558	55896647	-	0	0	1	0	11	0	0	0	1	0	miRNA
hsa-mir-4326	chr20	61918160	61918218	+	0	0	0	0	9	0	0	0	0	0	miRNA
hsa-mir-4330	chrX	1.5E+08	1.5E+08	+	0	0	0	0	0	0	0	0	23	0	miRNA
hsa-mir-4420	chr1	31212003	31212079	-	0	0	0	0	4	0	0	0	0	0	miRNA
hsa-mir-4424	chr1	1.79E+08	1.79E+08	+	0	0	0	1	0	0	0	0	0	0	miRNA
hsa-mir-4427	chr1	2.34E+08	2.34E+08	+	0	0	0	0	0	0	1	0	0	0	miRNA
hsa-mir-4434	chr2	64752647	64752699	+	0	0	0	0	0	0	0	1	0	0	miRNA
hsa-mir-4435-1	chr2	87929274	87929353	+	0	0	0	0	7	0	34	0	0	0	miRNA
hsa-mir-4435-2	chr2	1.12E+08	1.12E+08	-	0	0	0	0	7	0	34	0	0	0	miRNA
hsa-mir-4436a	chr2	89111884	89111968	+	0	0	0	0	0	0	0	0	19	0	miRNA
hsa-mir-4436b-1	chr2	1.11E+08	1.11E+08	-	0	0	0	0	10	0	0	0	0	0	miRNA
hsa-mir-4436b-2	chr2	1.11E+08	1.11E+08	+	0	0	0	0	10	0	0	0	0	0	miRNA
hsa-mir-4437	chr2	1.82E+08	1.82E+08	-	0	0	0	0	1	0	0	0	0	0	miRNA
hsa-mir-4440	chr2	2.4E+08	2.4E+08	-	0	0	0	0	8	0	0	0	18	14	miRNA

hsa-mir-4441	chr2	2.4E+08	2.4E+08	-	0	0	0	0	6	0	0	0	0	0	miRNA
hsa-mir-4444-1	chr2	1.78E+08	1.78E+08	+	0	0	34	0	0	0	0	0	0	0	miRNA
hsa-mir-4444-2	chr3	75263627	75263700	+	0	0	34	0	0	0	0	0	0	0	miRNA
hsa-mir-4445	chr3	1.09E+08	1.09E+08	+	0	0	0	0	0	0	0	0	0	23	miRNA
hsa-mir-4454	chr4	1.64E+08	1.64E+08	-	0	0	29	0	0	1	37	31	0	31	miRNA
hsa-mir-4458	chr5	8461038	8461112	+	0	0	0	0	1	0	0	0	0	0	miRNA
hsa-mir-4467	chr7	1.02E+08	1.02E+08	+	0	0	50	0	0	0	0	0	15	0	miRNA
hsa-mir-4471	chr8	1.01E+08	1.01E+08	+	0	0	24	0	0	0	0	0	0	0	miRNA
hsa-mir-4473	chr9	20411146	20411236	-	0	0	0	0	7	0	0	0	0	0	miRNA
hsa-mir-4477b	chr9	68415308	68415388	+	0	0	0	0	6	0	1	0	0	85	miRNA
hsa-mir-4481	chr10	12695137	12695196	-	0	0	0	0	1	0	0	0	0	0	miRNA
hsa-mir-4482	chr10	1.06E+08	1.06E+08	-	0	0	18	0	1	0	0	0	1	0	miRNA
hsa-mir-4484	chr10	1.28E+08	1.28E+08	+	0	0	0	0	2	0	0	0	0	0	miRNA
hsa-mir-4486	chr11	19596857	19596919	+	0	0	0	0	1	0	0	0	30	1	miRNA
hsa-mir-4487	chr11	47422521	47422593	+	0	0	64	0	0	0	0	0	0	0	miRNA
hsa-mir-4488	chr11	61276068	61276129	+	0	0	0	0	0	0	0	42	0	0	miRNA
hsa-mir-4489	chr11	65416663	65416724	+	0	0	0	0	0	0	0	0	0	17	miRNA
hsa-mir-4492	chr11	1.19E+08	1.19E+08	+	0	0	0	0	0	0	2	0	17	0	miRNA
hsa-mir-4497	chr12	1.1E+08	1.1E+08	+	0	0	0	0	0	0	0	0	1	0	miRNA
hsa-mir-4498	chr12	1.21E+08	1.21E+08	-	0	0	13	0	0	0	0	0	0	0	miRNA
hsa-mir-4499	chr13	21007917	21007985	-	0	0	0	0	0	0	0	0	0	1	miRNA
hsa-mir-449a	chr5	54466360	54466450	-	0	12	0	0	9	0	0	0	16	22	miRNA
hsa-mir-449b	chr5	54466474	54466570	-	0	1	0	0	0	0	0	0	0	0	miRNA
hsa-mir-450a-1	chrX	1.34E+08	1.34E+08	-	0	1	31	0	10	0	0	0	0	0	miRNA
hsa-mir-450a-2	chrX	1.34E+08	1.34E+08	-	0	61	100	0	137	0	39	0	29	79	miRNA
hsa-mir-450b	chrX	1.34E+08	1.34E+08	-	0	0	0	0	0	48	0	0	23	61	miRNA
hsa-mir-4514	chr15	81289758	81289814	-	0	0	0	0	4	0	0	0	0	0	miRNA
hsa-mir-4518	chr16	30515240	30515322	+	0	0	1	0	0	0	0	0	1	0	miRNA
hsa-mir-451b	chr17	27188389	27188456	+	0	0	6	0	0	0	0	0	0	4	miRNA
hsa-mir-4520-1	chr17	6558759	6558828	-	0	0	0	0	0	0	1	0	0	0	miRNA
hsa-mir-4520-2	chr17	6558768	6558821	+	0	0	0	0	0	0	1	0	0	0	miRNA
hsa-mir-4521	chr17	8090263	8090322	+	0	3	1	2	9	0	1	0	6	6	miRNA
hsa-mir-4525	chr17	80626109	80626183	-	0	0	0	0	5	0	0	0	19	0	miRNA
hsa-mir-4526	chr18	13611113	13611199	+	0	0	49	0	0	0	0	0	0	0	miRNA
hsa-mir-4527	chr18	44906867	44906936	+	0	0	0	0	0	0	0	1	0	0	miRNA
hsa-mir-455	chr9	1.17E+08	1.17E+08	+	0	0	0	0	8	0	0	76	1	0	miRNA
hsa-mir-4632	chr1	12251770	12251830	+	0	0	11	0	0	0	0	0	0	0	miRNA
hsa-mir-4635	chr5	1063011	1063089	-	0	0	0	0	0	0	0	0	0	1	miRNA
hsa-mir-4638	chr5	1.81E+08	1.81E+08	-	0	0	0	0	8	0	0	0	0	0	miRNA
hsa-mir-4639	chr6	16141787	16141855	+	0	0	26	0	0	0	0	0	0	0	miRNA
hsa-mir-4640	chr6	30858660	30858749	+	0	0	0	0	0	0	1	0	0	0	miRNA
hsa-mir-4644	chr6	1.71E+08	1.71E+08	+	0	0	0	0	0	0	0	0	1	0	miRNA
hsa-mir-4646	chr6	31668806	31668868	-	0	0	226	0	5	0	140	0	78	0	miRNA
hsa-mir-4647	chr6	44221943	44222022	-	0	0	0	0	0	0	0	0	14	0	miRNA
hsa-mir-4650-1	chr7	66579309	66579384	-	0	0	1	0	0	0	0	0	0	0	miRNA
hsa-mir-4650-2	chr7	72162874	72162949	+	0	0	1	0	0	0	0	0	0	0	miRNA
hsa-mir-4651	chr7	75544515	75544587	+	0	0	0	0	5	0	0	0	0	0	miRNA
hsa-mir-4653	chr7	1.01E+08	1.01E+08	+	0	0	18	0	0	0	0	0	1	0	miRNA
hsa-mir-4655	chr7	1883816	1883889	-	0	2	1	1	0	1	0	0	0	0	miRNA
hsa-mir-4656	chr7	4828196	4828270	-	0	93	0	0	0	0	0	0	0	0	miRNA
hsa-mir-4657	chr7	44921347	44921399	-	0	0	36	0	9	0	0	0	29	0	miRNA
hsa-mir-4659b	chr8	6602689	6602761	-	0	0	37	0	0	0	0	0	0	0	miRNA
hsa-mir-4660	chr8	8905955	8906028	+	0	0	0	0	12	0	0	0	12	0	miRNA
hsa-mir-4665	chr9	6007826	6007904	+	0	17	0	6	4	0	0	0	0	0	miRNA
hsa-mir-4667	chr9	35608091	35608156	+	0	0	20	0	0	0	0	0	0	0	miRNA
hsa-mir-4669	chr9	1.37E+08	1.37E+08	+	0	0	23	0	11	0	0	0	159	0	miRNA
hsa-mir-4671	chr1	2.34E+08	2.34E+08	+	0	0	0	0	0	0	1	0	0	1	miRNA
hsa-mir-4672	chr9	1.31E+08	1.31E+08	-	0	1	0	0	0	0	0	0	0	34	miRNA
hsa-mir-4673	chr9	1.39E+08	1.39E+08	-	0	0	0	0	0	0	0	0	6	0	miRNA
hsa-mir-4676	chr10	74480787	74480858	+	0	0	0	0	2	0	0	0	0	0	miRNA
hsa-mir-4677	chr1	2.44E+08	2.44E+08	+	0	24	0	0	2	0	0	0	0	0	miRNA
hsa-mir-4679-1	chr10	90823093	90823167	+	0	0	0	0	0	0	0	0	0	18	miRNA
hsa-mir-4683	chr10	35930100	35930180	-	0	0	0	0	9	0	0	0	0	0	miRNA
hsa-mir-4685	chr10	1E+08	1E+08	-	0	0	0	56	1	0	0	0	0	0	miRNA
hsa-mir-4687	chr11	3877292	3877371	+	0	0	0	0	2	0	0	0	1	0	miRNA
hsa-mir-4689	chr1	5922732	5922801	-	0	0	2	0	1	1	0	0	0	0	miRNA
hsa-mir-4690	chr11	65403781	65403840	+	0	0	0	0	0	0	0	0	0	1	miRNA
hsa-mir-4695	chr1	19209696	19209769	-	0	0	1	0	0	0	0	0	0	1	miRNA
hsa-mir-4701	chr12	49165758	49165820	-	0	0	0	0	0	0	0	0	12	0	miRNA
hsa-mir-4708	chr14	65801835	65801901	-	0	0	0	0	0	0	0	0	11	0	miRNA
hsa-mir-4711	chr1	60198899	60198968	-	0	0	0	0	6	0	0	0	0	0	miRNA
hsa-mir-4712	chr15	50652526	50652607	+	0	0	0	0	0	0	0	0	0	1	miRNA
hsa-mir-4713	chr15	51534387	51534461	+	0	0	0	0	1	0	0	0	0	0	miRNA
hsa-mir-4714	chr15	99327655	99327731	+	0	0	19	0	3	0	0	0	0	0	miRNA
hsa-mir-4716	chr15	49461267	49461350	-	0	0	59	0	0	0	0	0	1	0	miRNA
hsa-mir-4717	chr16	2324621	2324692	+	0	0	0	0	8	1	0	0	0	30	miRNA
hsa-mir-4725	chr17	29902288	29902377	+	0	0	0	0	9	0	0	0	1	0	miRNA
hsa-mir-4726	chr17	36875944	36876001	+	0	0	0	0	0	0	0	0	12	0	miRNA
hsa-mir-4734	chr17	36858515	36858584	-	0	0	0	0	0	1	0	0	0	0	miRNA
hsa-mir-4736	chr17	56413337	56413383	-	0	0	0	0	0	0	0	0	1	0	miRNA
hsa-mir-4738	chr17	73780602	73780688	-	0	0	18	0	0	0	0	0	0	0	miRNA
hsa-mir-4739	chr17	77680985	77681058	-	0	0	0	0	0	0	0	0	0	43	miRNA
hsa-mir-4740	chr17	79374516	79374578	-	0	0	0	0	1	0	0	0	0	0	miRNA

hsa-mir-4742	chr1	2.25E+08	2.25E+08	-	0	0	0	0	5	0	0	0	43	5	miRNA
hsa-mir-4743	chr18	46196971	46197039	+	0	0	0	0	0	0	0	0	21	0	miRNA
hsa-mir-4745	chr19	804940	805001	+	0	0	0	0	0	0	0	0	0	1	miRNA
hsa-mir-4746	chr19	4445975	4446045	+	0	0	0	0	5	0	0	0	0	0	miRNA
hsa-mir-4747	chr19	4932699	4932752	+	0	0	39	0	0	0	192	0	28	40	miRNA
hsa-mir-4748	chr19	10890930	10891011	+	0	0	0	0	0	0	0	0	39	0	miRNA
hsa-mir-4749	chr19	50357848	50357908	+	0	0	0	0	0	26	0	0	0	0	miRNA
hsa-mir-4750	chr19	50391432	50391487	+	0	0	1	36	4	0	0	0	0	46	miRNA
hsa-mir-4761	chr22	19951276	19951357	+	0	0	0	0	4	0	0	0	0	0	miRNA
hsa-mir-4762	chr22	46156404	46156478	+	0	0	0	0	14	0	6	0	0	0	miRNA
hsa-mir-4764	chr22	33832568	33832655	-	0	0	0	0	4	0	0	0	0	0	miRNA
hsa-mir-4766	chr22	41209887	41209962	-	0	0	0	0	7	0	107	0	0	0	miRNA
hsa-mir-4769	chrX	47446828	47446904	+	0	0	0	0	12	0	0	0	0	0	miRNA
hsa-mir-4772	chr2	1.03E+08	1.03E+08	+	0	0	0	137	92	0	0	0	0	108	miRNA
hsa-mir-4773-1	chr2	1.52E+08	1.52E+08	+	0	4	58	0	7	0	0	0	0	0	miRNA
hsa-mir-4773-2	chr2	1.52E+08	1.52E+08	-	0	4	58	0	7	0	0	0	0	0	miRNA
hsa-mir-4776-1	chr2	2.14E+08	2.14E+08	+	0	0	1	0	0	0	0	0	0	0	miRNA
hsa-mir-4784	chr2	1.32E+08	1.32E+08	-	0	0	0	0	0	0	13	0	21	0	miRNA
hsa-mir-4786	chr2	2.41E+08	2.41E+08	-	0	0	0	0	3	0	0	0	0	0	miRNA
hsa-mir-4791	chr3	19356340	19356423	-	0	0	0	0	3	0	0	0	0	0	miRNA
hsa-mir-4793	chr3	48681627	48681713	-	0	0	0	1	0	0	0	0	0	0	miRNA
hsa-mir-4795	chr3	87275339	87275427	-	0	0	27	0	0	0	0	0	0	0	miRNA
hsa-mir-4796	chr3	1.14E+08	1.14E+08	-	0	0	0	0	0	0	1	0	0	0	miRNA
hsa-mir-4800	chr4	2251804	2251883	-	0	0	0	0	4	0	0	0	0	0	miRNA
hsa-mir-4802	chr4	40504057	40504136	-	0	0	0	1	0	0	0	0	0	0	miRNA
hsa-mir-4804	chr5	72174418	72174490	+	0	0	2	0	3	0	48	0	3	0	miRNA
hsa-mir-485	chr14	1.02E+08	1.02E+08	+	0	0	0	125	176	0	0	0	0	0	miRNA
hsa-mir-487a	chr14	1.02E+08	1.02E+08	+	0	0	0	0	21	0	0	0	35	0	miRNA
hsa-mir-487b	chr14	1.02E+08	1.02E+08	+	0	30	35	294	504	0	0	0	41	0	miRNA
hsa-mir-489	chr7	93113248	93113331	-	0	0	33	0	0	0	0	0	0	0	miRNA
hsa-mir-490	chr7	1.37E+08	1.37E+08	+	0	0	0	0	0	0	0	0	20	0	miRNA
hsa-mir-495	chr14	1.02E+08	1.02E+08	+	0	0	0	0	30	0	0	0	0	0	miRNA
hsa-mir-496	chr14	1.02E+08	1.02E+08	+	0	0	0	0	2	0	0	0	0	0	miRNA
hsa-mir-497	chr17	6921230	6921341	-	0	0	56	0	0	0	0	0	0	28	miRNA
hsa-mir-4999	chr19	8454174	8454264	-	0	23	19	0	4	0	0	0	0	0	miRNA
hsa-mir-499a	chr20	33578179	33578300	+	0	0	45	0	9	0	0	0	0	0	miRNA
hsa-mir-5000	chr2	75317939	75318041	+	0	0	0	0	0	0	0	0	18	0	miRNA
hsa-mir-5001	chr2	2.33E+08	2.33E+08	-	0	18	18	0	0	0	0	0	12	0	miRNA
hsa-mir-5003	chr5	1.72E+08	1.72E+08	+	0	0	0	0	0	0	52	0	0	1	miRNA
hsa-mir-5006	chr13	42142422	42142531	-	0	0	0	0	3	0	0	0	0	0	miRNA
hsa-mir-5008	chr1	2.28E+08	2.28E+08	-	0	0	0	0	4	1	0	0	0	0	miRNA
hsa-mir-5009	chr15	90427163	90427262	-	0	0	5	0	0	0	0	0	0	0	miRNA
hsa-mir-500a	chrX	49773039	49773122	+	0	14	27	109	16	34	37	0	56	0	miRNA
hsa-mir-501	chrX	49774330	49774413	+	0	0	110	0	0	0	0	51	0	0	miRNA
hsa-mir-502	chrX	49779206	49779291	+	0	14	27	109	16	34	37	0	75	0	miRNA
hsa-mir-503	chrX	1.34E+08	1.34E+08	-	0	0	283	0	29	33	0	0	267	71	miRNA
hsa-mir-504	chrX	1.38E+08	1.38E+08	-	0	0	0	0	24	0	0	0	0	0	miRNA
hsa-mir-505	chrX	1.39E+08	1.39E+08	-	0	70	125	0	116	41	62	0	41	0	miRNA
hsa-mir-506	chrX	1.46E+08	1.46E+08	-	0	0	0	0	5	0	0	0	0	0	miRNA
hsa-mir-509-2	chrX	1.46E+08	1.46E+08	-	0	0	0	0	0	0	0	0	1	0	miRNA
hsa-mir-509-3	chrX	1.46E+08	1.46E+08	-	0	0	0	0	0	0	0	0	1	0	miRNA
hsa-mir-5092	chr3	1.25E+08	1.25E+08	-	0	0	0	1	0	0	0	0	18	0	miRNA
hsa-mir-5093	chr16	85339832	85339931	-	0	0	0	0	0	0	0	1	0	0	miRNA
hsa-mir-5094	chr15	90393869	90393953	-	0	14	0	0	0	0	0	0	0	0	miRNA
hsa-mir-5100	chr10	43493011	43493129	+	0	0	0	0	7	0	0	0	0	0	miRNA
hsa-mir-511	chr10	18134036	18134122	+	0	0	186	56	17	0	0	0	0	0	miRNA
hsa-mir-512-2	chr19	54172411	54172508	+	0	0	0	0	1	0	0	0	0	0	miRNA
hsa-mir-516b-1	chr19	54240099	54240188	+	0	0	0	0	5	0	0	0	0	0	miRNA
hsa-mir-516b-2	chr19	54228696	54228780	+	0	0	0	0	5	0	0	0	0	0	miRNA
hsa-mir-5186	chr3	1.51E+08	1.51E+08	-	0	0	0	0	0	0	0	0	18	0	miRNA
hsa-mir-5188	chr12	1.25E+08	1.25E+08	+	0	0	0	0	0	0	1	0	0	1	miRNA
hsa-mir-5189	chr16	88535326	88535439	+	0	0	67	37	11	0	0	0	30	0	miRNA
hsa-mir-518a-2	chr19	54242587	54242673	+	0	0	0	0	0	0	0	4	1	0	miRNA
hsa-mir-5191	chr1	2.02E+08	2.02E+08	+	0	0	7	0	0	0	0	0	0	0	miRNA
hsa-mir-526a-2	chr19	54230176	54230240	+	0	0	0	0	0	0	0	0	0	36	miRNA
hsa-mir-539	chr14	1.02E+08	1.02E+08	+	0	0	0	0	35	0	0	0	0	0	miRNA
hsa-mir-544a	chr14	1.02E+08	1.02E+08	+	0	0	0	0	0	1	0	0	0	0	miRNA
hsa-mir-544b	chr3	1.24E+08	1.24E+08	+	0	0	0	0	0	0	0	0	12	0	miRNA
hsa-mir-545	chrX	73506939	73507044	-	0	0	0	0	14	0	0	0	0	0	miRNA
hsa-mir-548ab	chr3	1.03E+08	1.03E+08	-	0	5	6	0	1	0	3	0	4	1	miRNA
hsa-mir-548ac	chr1	1.17E+08	1.17E+08	-	0	22	0	0	29	0	0	0	0	0	miRNA
hsa-mir-548ae-1	chr2	1.85E+08	1.85E+08	+	0	0	0	0	0	0	1	0	0	0	miRNA
hsa-mir-548ag-1	chr4	61788337	61788402	+	0	0	0	0	9	0	0	0	0	0	miRNA
hsa-mir-548ag-2	chr20	59139620	59139683	+	0	0	0	0	9	0	0	0	0	32	miRNA
hsa-mir-548ah	chr4	77496704	77496779	+	0	0	0	0	11	0	0	0	0	0	miRNA
hsa-mir-548aj-1	chr6	1.32E+08	1.32E+08	-	0	0	0	0	0	0	1	0	0	0	miRNA
hsa-mir-548ak	chr10	12172759	12172815	-	0	0	23	0	4	0	0	0	18	43	miRNA
hsa-mir-548al	chr11	74110282	74110378	+	0	0	0	0	8	0	0	0	0	0	miRNA
hsa-mir-548am	chrX	16645135	16645208	-	0	25	236	1	17	39	41	0	75	46	miRNA
hsa-mir-548aq	chr3	1.85E+08	1.85E+08	-	0	0	12	0	0	0	0	0	15	0	miRNA
hsa-mir-548as	chr13	93142416	93142473	+	0	0	37	0	0	0	0	0	0	0	miRNA
hsa-mir-548at	chr17	40646791	40646848	+	0	0	160	0	0	0	23	0	76	0	miRNA
hsa-mir-548av	chr18	70520556	70520617	-	0	0	58	0	0	0	0	0	74	23	miRNA

hsa-mir-548aw	chr9	1.36E+08	1.36E+08	+	0	0	23	0	0	0	0	0	0	0	miRNA
hsa-mir-548ax	chrX	11336734	11336806	-	0	0	59	0	1	0	0	0	0	0	miRNA
hsa-mir-548az	chr8	1.2E+08	1.2E+08	+	0	0	24	0	0	0	0	0	0	0	miRNA
hsa-mir-548b	chr6	1.19E+08	1.19E+08	-	0	1	41	1	1	47	1	0	29	45	miRNA
hsa-mir-548bc	chr17	37453653	37453762	+	0	0	1	0	0	0	0	0	0	0	miRNA
hsa-mir-548c	chr12	65016289	65016385	+	0	9	236	0	17	39	41	0	75	45	miRNA
hsa-mir-548e	chr10	1.13E+08	1.13E+08	+	0	23	85	0	20	32	3	0	12	0	miRNA
hsa-mir-548f-1	chr10	56367634	56367717	-	0	0	1	0	0	0	1	0	0	0	miRNA
hsa-mir-548f-2	chr2	2.13E+08	2.13E+08	-	0	0	0	0	0	45	0	0	0	0	miRNA
hsa-mir-548f-3	chr5	1.1E+08	1.1E+08	-	0	0	0	0	0	45	0	0	0	0	miRNA
hsa-mir-548f-4	chr7	1.47E+08	1.47E+08	-	0	0	7	0	0	0	3	0	5	7	miRNA
hsa-mir-548g	chr4	1.48E+08	1.48E+08	-	0	0	0	0	0	0	0	0	1	0	miRNA
hsa-mir-548i-1	chr3	1.26E+08	1.26E+08	-	0	0	3	0	1	0	0	0	1	0	miRNA
hsa-mir-548i-2	chr4	9557789	9557937	-	0	0	3	0	1	0	0	0	1	0	miRNA
hsa-mir-548i-3	chr8	7946463	7946611	-	0	0	3	0	1	0	0	0	1	0	miRNA
hsa-mir-548i-4	chrX	83480760	83480836	-	0	0	3	0	5	0	0	0	1	0	miRNA
hsa-mir-548k	chr11	70130061	70130176	+	0	0	0	0	0	0	49	0	28	0	miRNA
hsa-mir-548l	chr11	94199661	94199746	-	0	18	204	0	92	0	46	0	198	47	miRNA
hsa-mir-548n	chr7	34980372	34980446	-	0	28	6	0	10	0	37	0	4	1	miRNA
hsa-mir-548o	chr7	1.02E+08	1.02E+08	-	0	0	58	0	27	0	0	0	74	23	miRNA
hsa-mir-548o-2	chr20	37145206	37145275	+	0	9	294	0	44	39	41	0	149	68	miRNA
hsa-mir-548q	chr10	12767253	12767352	-	0	0	275	0	28	4	0	0	16	0	miRNA
hsa-mir-548t	chr4	1.74E+08	1.74E+08	+	0	0	0	0	1	0	0	0	0	0	miRNA
hsa-mir-548u	chr6	57254930	57255010	+	0	0	0	0	0	0	0	0	0	6	miRNA
hsa-mir-548v	chr8	17539087	17539166	-	0	0	8	0	0	0	0	0	1	0	miRNA
hsa-mir-548w	chr16	26036558	26036631	+	0	0	23	0	4	0	0	0	18	43	miRNA
hsa-mir-548x-2	chr13	66540462	66540561	-	0	0	0	0	0	1	0	0	0	0	miRNA
hsa-mir-548y	chr14	48230198	48230307	-	0	0	0	0	0	0	0	1	0	0	miRNA
hsa-mir-550a-1	chr7	30329410	30329506	+	0	0	102	28	55	0	27	0	181	192	miRNA
hsa-mir-550a-3	chr7	29720350	29720444	-	0	0	45	27	16	0	27	0	128	151	miRNA
hsa-mir-550b-1	chr7	30329410	30329506	-	0	0	0	0	0	0	0	0	3	50	miRNA
hsa-mir-550b-2	chr7	32772593	32772689	-	0	0	0	0	0	0	0	0	3	50	miRNA
hsa-mir-551a	chr1	3477259	3477354	-	0	25	0	0	3	0	0	0	0	0	miRNA
hsa-mir-551b	chr3	1.68E+08	1.68E+08	+	0	1	0	0	4	0	0	0	0	0	miRNA
hsa-mir-552	chr1	35135200	35135295	-	0	0	0	0	8	2	0	0	0	0	miRNA
hsa-mir-554	chr1	1.52E+08	1.52E+08	+	0	0	0	0	0	1	0	0	0	0	miRNA
hsa-mir-555	chr1	1.55E+08	1.55E+08	-	0	0	0	0	1	0	0	0	0	0	miRNA
hsa-mir-556	chr1	1.62E+08	1.62E+08	+	0	0	0	0	6	0	0	0	0	0	miRNA
hsa-mir-5583-1	chr18	37256685	37256743	+	0	0	0	0	7	0	0	0	0	0	miRNA
hsa-mir-5583-2	chr18	37256683	37256741	-	0	0	0	0	7	0	0	0	0	0	miRNA
hsa-mir-5584	chr1	45011165	45011224	+	0	0	0	72	17	0	49	0	0	0	miRNA
hsa-mir-5586	chr14	60113680	60113738	-	0	25	0	0	24	0	0	0	0	0	miRNA
hsa-mir-5588	chr3	1.85E+08	1.85E+08	-	0	0	0	0	3	0	0	0	0	0	miRNA
hsa-mir-5589	chr19	10149030	10149089	+	0	0	0	0	4	0	29	0	0	0	miRNA
hsa-mir-559	chr2	47604814	47604909	+	0	0	28	0	0	0	0	0	0	0	miRNA
hsa-mir-5591	chr4	39413530	39413594	+	0	0	0	34	0	0	0	0	0	0	miRNA
hsa-mir-5680	chr8	1.03E+08	1.03E+08	+	0	0	0	0	0	0	0	0	6	0	miRNA
hsa-mir-5685	chr6	53141791	53141869	+	0	16	0	0	0	0	0	0	0	0	miRNA
hsa-mir-5692b	chr21	44371038	44371124	-	0	0	0	0	0	1	0	0	0	0	miRNA
hsa-mir-5695	chr19	13031134	13031218	+	0	0	21	0	0	0	0	0	0	0	miRNA
hsa-mir-5696	chr2	1.02E+08	1.02E+08	+	0	0	0	0	0	0	0	0	0	56	miRNA
hsa-mir-5697	chr1	10027439	10027516	+	0	0	0	0	0	0	0	0	15	0	miRNA
hsa-mir-5698	chr1	1.54E+08	1.54E+08	-	0	0	0	0	0	0	46	0	0	0	miRNA
hsa-mir-5699	chr10	687629	687718	-	0	16	0	0	6	0	0	0	0	0	miRNA
hsa-mir-5706	chr5	1.18E+08	1.18E+08	+	0	0	0	0	0	0	0	0	0	1	miRNA
hsa-mir-572	chr4	11370451	11370545	+	0	245	0	0	0	0	0	0	0	0	miRNA
hsa-mir-5739	chr22	28855857	28855936	+	0	0	0	0	0	0	0	0	0	1	miRNA
hsa-mir-574	chr4	38869653	38869748	+	0	45	53	69	244	0	77	0	0	53	miRNA
hsa-mir-5787	chr3	50264868	50264922	+	0	0	0	0	0	0	0	0	1	0	miRNA
hsa-mir-581	chr5	53247334	53247429	-	0	0	0	0	11	0	0	0	0	0	miRNA
hsa-mir-582	chr5	58999432	58999529	-	0	59	21	66	132	0	36	43	0	61	miRNA
hsa-mir-587	chr6	1.07E+08	1.07E+08	+	0	0	0	0	0	0	0	0	0	1	miRNA
hsa-mir-589	chr7	5535450	5535548	-	0	54	121	0	42	0	41	0	97	43	miRNA
hsa-mir-590	chr7	73605528	73605624	+	0	0	81	45	29	0	0	0	14	97	miRNA
hsa-mir-592	chr7	1.27E+08	1.27E+08	-	0	0	22	2	30	0	0	0	0	0	miRNA
hsa-mir-593	chr7	1.28E+08	1.28E+08	+	0	0	0	0	0	0	0	0	1	0	miRNA
hsa-mir-597	chr8	9599182	9599278	+	0	0	52	0	10	0	0	0	0	19	miRNA
hsa-mir-598	chr8	10892716	10892812	-	0	104	599	0	240	154	130	0	144	240	miRNA
hsa-mir-604	chr10	29833933	29834026	-	0	0	0	0	8	0	0	0	0	0	miRNA
hsa-mir-6069	chr22	35732714	35732792	-	0	1	0	0	0	0	0	0	0	0	miRNA
hsa-mir-607	chr10	98588426	98588521	-	0	0	56	0	0	0	0	0	3	0	miRNA
hsa-mir-6074	chr12	66417400	66417506	-	0	0	0	0	0	0	0	0	1	0	miRNA
hsa-mir-6076	chr14	50433117	50433229	+	0	0	0	0	12	0	0	0	39	0	miRNA
hsa-mir-6080	chr17	62776877	62776942	+	0	0	0	0	2	0	0	0	0	0	miRNA
hsa-mir-6085	chr15	62635228	62635337	+	0	0	0	0	9	0	0	0	0	0	miRNA
hsa-mir-6086	chrX	13608411	13608465	+	0	0	0	0	8	0	0	0	0	0	miRNA
hsa-mir-610	chr11	28078362	28078457	+	0	0	9	0	0	0	0	0	24	12	miRNA
hsa-mir-6125	chr12	62654140	62654235	+	0	23	83	57	38	59	1	0	4	0	miRNA
hsa-mir-6127	chr1	22959751	22959859	-	0	0	0	1	0	0	0	0	0	0	miRNA
hsa-mir-6128	chr11	56511349	56511457	+	0	0	0	0	0	0	0	1	0	0	miRNA
hsa-mir-6130	chr21	24451606	24451714	+	0	0	1	0	4	0	0	0	1	3	miRNA
hsa-mir-616	chr12	57912946	57913042	-	0	17	45	3	74	0	34	0	19	16	miRNA
hsa-mir-618	chr12	81329515	81329612	-	0	0	0	127	93	33	4	0	0	0	miRNA

hsa-mir-622	chr13	90883436	90883531	+	0	0	0	0	17	0	0	0	0	0	miRNA
hsa-mir-624	chr14	31483852	31483948	-	0	0	11	0	4	0	0	0	29	0	miRNA
hsa-mir-627	chr15	42491768	42491864	-	0	24	127	0	53	0	46	0	60	118	miRNA
hsa-mir-632	chr17	30677128	30677221	+	0	0	0	0	0	0	26	0	0	0	miRNA
hsa-mir-634	chr17	64783190	64783286	+	0	0	0	0	0	0	1	0	0	0	miRNA
hsa-mir-636	chr17	74732532	74732630	-	0	0	0	0	3	0	73	0	20	0	miRNA
hsa-mir-638	chr19	10829080	10829179	+	0	12	0	0	0	0	0	0	0	0	miRNA
hsa-mir-639	chr19	14640355	14640452	+	0	0	0	0	7	0	0	0	0	0	miRNA
hsa-mir-641	chr19	40788450	40788548	-	0	27	49	0	35	41	0	0	92	66	miRNA
hsa-mir-642a	chr19	46178186	46178282	+	0	0	0	57	35	0	0	0	0	51	miRNA
hsa-mir-642b	chr19	46178190	46178266	-	0	0	0	0	0	0	0	0	0	4	miRNA
hsa-mir-643	chr19	52785050	52785146	+	0	0	0	0	1	0	0	0	0	0	miRNA
hsa-mir-645	chr20	49202323	49202416	+	0	0	0	0	0	0	0	0	0	44	miRNA
hsa-mir-647	chr20	62573984	62574079	-	0	0	6	0	0	0	0	0	0	0	miRNA
hsa-mir-648	chr22	18463634	18463727	-	0	0	0	0	0	1	0	0	0	0	miRNA
hsa-mir-6499	chr5	1.51E+08	1.51E+08	-	0	1	0	0	0	0	0	0	0	0	miRNA
hsa-mir-6501	chr21	34922968	34923034	+	0	0	0	0	0	0	0	0	5	0	miRNA
hsa-mir-6505	chr12	48526580	48526650	+	0	0	0	0	0	0	0	0	0	30	miRNA
hsa-mir-6509	chr7	1.35E+08	1.35E+08	-	0	0	0	53	9	0	0	0	0	50	miRNA
hsa-mir-651	chrX	8095006	8095102	+	0	57	369	0	138	0	42	0	183	71	miRNA
hsa-mir-6513	chr2	2.19E+08	2.19E+08	-	0	0	46	0	46	1	8	0	0	0	miRNA
hsa-mir-6514	chr11	62560174	62560243	-	0	15	12	0	0	0	36	0	39	0	miRNA
hsa-mir-6516	chr17	75085499	75085579	+	0	26	0	0	3	0	0	0	0	0	miRNA
hsa-mir-659	chr22	38243685	38243781	-	0	0	0	0	5	0	0	0	0	0	miRNA
hsa-mir-662	chr16	820183	820277	+	0	1	0	0	0	0	0	0	0	0	miRNA
hsa-mir-664a	chr1	2.2E+08	2.2E+08	-	0	50	140	54	72	1	4	0	44	112	miRNA
hsa-mir-664b	chrX	1.54E+08	1.54E+08	+	0	0	0	0	5	58	0	0	13	0	miRNA
hsa-mir-670	chr11	43581206	43581303	+	0	0	0	0	0	0	0	0	1	0	miRNA
hsa-mir-6715a	chr10	1.14E+08	1.14E+08	+	0	0	0	0	7	0	0	0	0	0	miRNA
hsa-mir-6716	chr11	1.19E+08	1.19E+08	+	0	0	0	0	0	0	0	0	18	0	miRNA
hsa-mir-6717	chr14	21491473	21491545	-	0	0	0	0	7	0	0	0	0	0	miRNA
hsa-mir-6720	chr6	1390549	1390646	-	0	0	0	0	0	0	0	0	2	0	miRNA
hsa-mir-6721	chr6	32137807	32137893	-	0	0	0	47	43	23	0	0	19	57	miRNA
hsa-mir-6724-1	chrUn_gl0(	104732	104823	+	0	14	59	15	23	17	0	22	51	0	miRNA
hsa-mir-6724-2	chrUn_gl0(	148704	148795	+	0	14	59	15	23	17	0	22	51	0	miRNA
hsa-mir-6724-3	chrUn_gl0(	104732	104823	+	0	14	59	15	23	17	0	22	51	0	miRNA
hsa-mir-6724-4	chrUn_gl0(	148704	148795	+	0	14	59	15	23	17	0	22	51	0	miRNA
hsa-mir-6726	chr1	1231490	1231550	-	0	0	0	0	0	0	4	0	0	0	miRNA
hsa-mir-6730	chr1	12638985	12639051	-	0	0	0	0	1	0	0	0	37	0	miRNA
hsa-mir-6731	chr1	25245836	25245907	-	0	0	0	0	0	0	0	0	15	0	miRNA
hsa-mir-6732	chr1	37945831	37945890	+	0	0	0	0	6	0	0	0	0	0	miRNA
hsa-mir-6734	chr1	43830319	43830386	-	0	0	84	0	22	24	99	0	0	1	miRNA
hsa-mir-6742	chr1	2.29E+08	2.29E+08	-	0	0	0	0	1	0	0	0	0	0	miRNA
hsa-mir-6744	chr11	1277835	1277900	+	0	23	0	0	0	0	0	0	0	0	miRNA
hsa-mir-6745	chr11	47201162	47201288	-	0	1	0	1	0	1	0	0	0	0	miRNA
hsa-mir-6747	chr11	62334483	62334543	-	0	0	0	0	21	0	0	0	44	0	miRNA
hsa-mir-6748	chr11	62557287	62557357	+	0	0	0	0	0	42	0	0	0	0	miRNA
hsa-mir-675	chr11	2017989	2018061	-	0	0	13	0	0	0	0	0	0	21	miRNA
hsa-mir-6750	chr11	64665835	64665909	-	0	15	0	0	0	0	1	0	3	0	miRNA
hsa-mir-6751	chr11	64897388	64897450	-	0	0	12	0	1	0	19	0	0	0	miRNA
hsa-mir-6752	chr11	67257716	67257786	+	0	0	1	0	0	0	1	0	0	0	miRNA
hsa-mir-6754	chr11	71184549	71184614	+	0	0	0	0	0	0	8	0	0	0	miRNA
hsa-mir-6755	chr11	85989375	85989440	+	0	0	0	0	11	0	0	0	0	0	miRNA
hsa-mir-6758	chr12	57906471	57906533	+	0	0	9	0	0	69	0	0	0	0	miRNA
hsa-mir-6761	chr12	1.12E+08	1.12E+08	+	0	0	0	0	5	0	0	0	0	0	miRNA
hsa-mir-6762	chr12	1.14E+08	1.14E+08	+	0	0	1	0	0	0	0	0	14	0	miRNA
hsa-mir-6763	chr12	1.33E+08	1.33E+08	+	0	0	0	0	8	17	0	0	0	47	miRNA
hsa-mir-6764	chr14	1.01E+08	1.01E+08	+	0	0	0	0	3	0	0	0	0	47	miRNA
hsa-mir-6767	chr16	2495393	2495458	+	0	0	0	0	0	0	0	0	13	0	miRNA
hsa-mir-6769a	chr16	4721319	4721391	+	0	0	20	0	0	0	0	0	0	0	miRNA
hsa-mir-6771	chr16	50326527	50326586	+	0	1	0	0	0	0	0	0	0	0	miRNA
hsa-mir-6777	chr17	17716794	17716859	-	0	0	0	0	5	14	6	0	22	0	miRNA
hsa-mir-6780a	chr17	40860102	40860169	-	0	0	35	0	5	0	157	0	57	0	miRNA
hsa-mir-6781	chr17	40975898	40975961	-	0	0	0	0	0	0	0	0	27	0	miRNA
hsa-mir-6783	chr17	43011986	43012049	-	0	0	0	0	9	0	0	0	0	0	miRNA
hsa-mir-6785	chr17	73494629	73494709	+	0	0	14	0	0	0	29	1	27	0	miRNA
hsa-mir-6786	chr17	79660787	79660899	+	0	0	0	0	3	3	0	0	0	0	miRNA
hsa-mir-6790	chr19	6392932	6392994	-	0	0	0	1	0	0	0	0	0	0	miRNA
hsa-mir-6791	chr19	6736723	6736789	-	0	0	0	0	0	39	0	0	0	0	miRNA
hsa-mir-6793	chr19	10939649	10939711	+	0	0	64	0	27	23	0	0	0	0	miRNA
hsa-mir-6794	chr19	12963074	12963141	+	0	0	0	0	0	0	36	0	0	0	miRNA
hsa-mir-6800	chr19	50335275	50335356	+	0	0	1	0	0	0	0	0	0	0	miRNA
hsa-mir-6802	chr19	55751280	55751344	-	0	0	53	2	9	0	1	0	0	0	miRNA
hsa-mir-6804	chr19	55742253	55742320	-	0	0	0	0	0	0	0	0	43	0	miRNA
hsa-mir-6805	chr19	55899549	55899610	+	0	13	23	0	2	0	0	0	27	29	miRNA
hsa-mir-6807	chr19	59061652	59061743	+	0	0	0	0	6	0	0	0	0	0	miRNA
hsa-mir-6808	chr1	1275030	1275088	-	0	0	0	0	1	0	0	0	0	0	miRNA
hsa-mir-6810	chr2	2.19E+08	2.19E+08	+	0	0	0	0	25	0	0	0	0	0	miRNA
hsa-mir-6812	chr20	44054150	44054213	+	0	0	0	0	0	0	31	0	0	0	miRNA
hsa-mir-6813	chr20	62708308	62708363	-	0	0	10	0	30	0	0	0	0	0	miRNA
hsa-mir-6815	chr21	46898180	46898240	+	0	0	1	0	0	0	0	0	0	0	miRNA
hsa-mir-6817	chr22	25851613	25851678	+	0	0	0	0	0	0	0	0	1	0	miRNA
hsa-mir-6819	chr22	36682893	36682953	-	0	0	16	0	6	0	0	1	0	0	miRNA

hsa-mir-6820	chr22	38363570	38363631	+	0	0	0	0	7	1	0	0	0	0	miRNA
hsa-mir-6823	chr3	48587394	48587454	-	0	0	1	0	0	0	0	0	0	0	miRNA
hsa-mir-6824	chr3	48671069	48671131	-	0	0	0	0	6	0	21	0	13	0	miRNA
hsa-mir-6825	chr3	1.27E+08	1.27E+08	-	0	0	6	0	0	0	0	0	4	0	miRNA
hsa-mir-6826	chr3	1.29E+08	1.29E+08	+	0	0	0	0	9	0	0	0	29	0	miRNA
hsa-mir-6830	chr5	1.32E+08	1.32E+08	-	0	0	0	0	0	0	0	0	1	0	miRNA
hsa-mir-6831	chr5	1.4E+08	1.4E+08	-	0	0	0	0	1	0	0	0	0	0	miRNA
hsa-mir-6832	chr6	31601564	31601635	+	0	0	19	0	20	0	0	0	0	0	miRNA
hsa-mir-6833	chr6	32147593	32147653	+	0	0	87	0	10	0	0	0	17	0	miRNA
hsa-mir-6835	chr6	34208450	34208513	+	0	0	0	0	0	0	0	0	8	0	miRNA
hsa-mir-6837	chr7	44091365	44091428	+	0	0	0	0	4	0	0	0	0	0	miRNA
hsa-mir-6838	chr7	44112977	44113032	-	0	0	0	0	0	0	0	0	2	0	miRNA
hsa-mir-6843	chr8	27468118	27468268	-	0	0	0	0	10	0	0	0	0	0	miRNA
hsa-mir-6845	chr8	1.45E+08	1.45E+08	-	0	0	0	0	0	36	0	0	0	0	miRNA
hsa-mir-6847	chr8	1.45E+08	1.45E+08	+	0	0	0	0	0	0	36	0	0	0	miRNA
hsa-mir-6849	chr8	1.46E+08	1.46E+08	-	0	0	0	0	0	0	0	0	0	30	miRNA
hsa-mir-6850	chr8	1.46E+08	1.46E+08	-	0	0	0	0	0	0	0	0	21	0	miRNA
hsa-mir-6858	chrX	1.54E+08	1.54E+08	+	0	0	0	0	0	0	0	0	11	0	miRNA
hsa-mir-6859-1	chr1	17369	17436	-	0	0	56	0	0	0	0	0	0	24	miRNA
hsa-mir-6859-3	chr15	1.03E+08	1.03E+08	+	0	0	56	0	0	0	0	0	0	24	miRNA
hsa-mir-6859-4	chr16	67052	67119	-	0	0	56	0	0	0	0	0	0	24	miRNA
hsa-mir-6860	chr11	66813114	66813179	+	0	0	0	0	4	0	0	0	0	0	miRNA
hsa-mir-6862-1	chr16	28402303	28402372	-	0	0	1	0	0	0	0	0	0	0	miRNA
hsa-mir-6862-2	chr16	28735573	28735642	+	0	0	1	0	0	0	0	0	0	0	miRNA
hsa-mir-6866	chr17	38318186	38318254	+	0	0	0	0	0	0	0	0	14	0	miRNA
hsa-mir-6867	chr17	38349849	38349915	+	0	0	29	0	6	0	0	0	0	0	miRNA
hsa-mir-6868	chr17	74094100	74094157	-	0	0	17	0	0	0	0	0	0	0	miRNA
hsa-mir-6872	chr3	50310667	50310728	+	0	0	0	0	0	0	0	0	0	58	miRNA
hsa-mir-6873	chr6	33255004	33255066	-	0	0	1	0	0	0	0	0	0	0	miRNA
hsa-mir-6879	chr11	64785977	64786042	+	0	0	0	0	0	1	0	0	0	0	miRNA
hsa-mir-6881	chr15	74703698	74703773	-	0	0	0	0	0	0	20	0	0	0	miRNA
hsa-mir-6882	chr15	75132983	75133048	-	0	20	17	64	33	0	44	0	0	39	miRNA
hsa-mir-6883	chr17	8048312	8048389	-	0	0	0	0	0	0	2	0	0	0	miRNA
hsa-mir-6889	chr22	41648996	41649054	-	0	1	0	0	0	0	0	0	0	0	miRNA
hsa-mir-6891	chr6	31323001	31323093	-	0	0	71	0	0	0	0	0	0	2	miRNA
hsa-mir-6892	chr7	1.43E+08	1.43E+08	+	0	0	0	0	0	0	0	0	35	0	miRNA
hsa-mir-6894	chrX	53228071	53228127	-	0	0	44	0	0	0	41	0	16	0	miRNA
hsa-mir-708	chr11	79113066	79113153	-	0	0	58	0	0	0	0	0	0	8	miRNA
hsa-mir-7108	chr19	2434912	2434998	-	0	15	0	0	0	0	0	0	0	0	miRNA
hsa-mir-711	chr3	48616335	48616410	-	0	0	0	0	0	0	0	0	23	0	miRNA
hsa-mir-7150	chr9	1.26E+08	1.26E+08	-	0	0	0	1	0	1	0	0	0	1	miRNA
hsa-mir-7155	chr11	64109321	64109376	-	0	0	9	0	6	0	0	0	19	14	miRNA
hsa-mir-7156	chr1	77525828	77525887	+	0	0	0	0	0	0	0	0	0	1	miRNA
hsa-mir-7159	chr6	33866912	33866977	+	0	0	0	0	2	0	0	0	0	0	miRNA
hsa-mir-7161	chr6	1.59E+08	1.59E+08	+	0	0	0	0	0	0	0	0	1	1	miRNA
hsa-mir-718	chrX	1.53E+08	1.53E+08	-	0	0	0	0	0	0	0	0	0	1	miRNA
hsa-mir-758	chr14	1.01E+08	1.01E+08	+	0	0	0	0	18	0	0	0	0	0	miRNA
hsa-mir-762	chr16	30905224	30905306	+	0	0	0	0	0	0	0	0	15	0	miRNA
hsa-mir-764	chrX	1.14E+08	1.14E+08	+	0	0	0	0	4	0	0	0	0	0	miRNA
hsa-mir-7705	chr8	1.02E+08	1.02E+08	-	0	0	0	0	0	0	0	0	0	38	miRNA
hsa-mir-7706	chr15	85923827	85923893	+	0	37	32	0	32	0	75	0	0	0	miRNA
hsa-mir-7843	chr14	72983528	72983606	+	0	1	0	0	2	0	0	0	0	0	miRNA
hsa-mir-7845	chr2	2.08E+08	2.08E+08	+	0	0	0	0	3	0	1	0	0	0	miRNA
hsa-mir-7846	chr1	12227000	12227095	+	0	1	0	0	12	0	0	1	0	0	miRNA
hsa-mir-7847	chr11	1901275	1901377	+	0	0	0	0	7	0	0	0	0	2	miRNA
hsa-mir-7849	chr4	1.47E+08	1.47E+08	+	0	0	0	0	6	0	0	0	0	0	miRNA
hsa-mir-7850	chr19	2630713	2630791	+	0	0	0	0	23	0	0	0	0	0	miRNA
hsa-mir-7851	chr12	42717502	42717661	-	0	0	0	0	1	0	0	0	0	0	miRNA
hsa-mir-7854	chr16	81567507	81567571	+	0	0	0	0	29	15	0	0	1	0	miRNA
hsa-mir-7973-1	chr15	51606231	51606306	+	0	1	0	0	12	0	0	0	0	0	miRNA
hsa-mir-7973-2	chr15	51606229	51606304	-	0	1	0	0	0	0	0	0	0	0	miRNA
hsa-mir-7975	chr19	55634593	55634660	-	0	0	0	0	0	0	1	0	0	0	miRNA
hsa-mir-7977	chr3	1.76E+08	1.76E+08	+	0	11	0	0	0	0	0	0	0	0	miRNA
hsa-mir-7978	chr4	21466323	21466381	-	0	0	10	0	0	0	0	0	0	0	miRNA
hsa-mir-8053	chr4	47654686	47654760	+	0	0	0	0	2	0	0	0	0	0	miRNA
hsa-mir-8060	chr3	96078808	96078883	+	0	0	0	0	1	0	0	0	0	0	miRNA
hsa-mir-8072	chr12	1.24E+08	1.24E+08	-	0	1	0	0	0	0	46	0	25	0	miRNA
hsa-mir-8077	chr19	42855283	42855357	+	0	0	0	0	0	0	0	0	13	0	miRNA
hsa-mir-8080	chr2	80093621	80093709	-	0	0	0	0	10	0	0	0	16	0	miRNA
hsa-mir-8083	chr1	1.54E+08	1.54E+08	-	0	0	2	0	0	0	0	0	0	0	miRNA
hsa-mir-873	chr9	28888877	28888953	-	0	12	160	0	8	43	0	0	0	33	miRNA
hsa-mir-874	chr5	1.37E+08	1.37E+08	-	0	0	0	0	0	0	0	0	0	45	miRNA
hsa-mir-876	chr9	28863624	28863704	-	0	0	0	0	4	0	0	0	0	0	miRNA
hsa-mir-885	chr3	10436173	10436246	-	0	0	0	0	171	0	10	0	13	0	miRNA
hsa-mir-889	chr14	1.02E+08	1.02E+08	+	0	0	0	154	71	0	0	0	0	0	miRNA
hsa-mir-890	chrX	1.45E+08	1.45E+08	-	0	0	1	0	0	0	0	0	0	0	miRNA
hsa-mir-892a	chrX	1.45E+08	1.45E+08	-	0	0	0	0	0	0	0	0	12	0	miRNA
hsa-mir-921	chr1	1.66E+08	1.66E+08	-	0	0	0	0	0	1	0	0	0	0	miRNA
hsa-mir-937	chr8	1.45E+08	1.45E+08	-	0	10	0	0	0	37	0	0	20	68	miRNA
hsa-mir-939	chr8	1.46E+08	1.46E+08	-	0	0	79	0	0	14	5	0	33	17	miRNA
hsa-mir-940	chr16	2321748	2321841	+	0	0	26	0	0	0	0	0	0	0	miRNA
hsa-mir-942	chr1	1.18E+08	1.18E+08	+	0	40	388	0	91	31	125	0	219	79	miRNA
hsa-mir-943	chr4	1988111	1988204	-	0	1	0	0	0	0	0	0	0	0	miRNA

hsa-mir-95	chr4	8007028	8007108	-	0	0	37	0	27	0	0	0	17	46	miRNA
hsa-mir-9500	chr2	2.2E+08	2.2E+08	+	0	0	0	0	8	0	0	0	0	0	miRNA
hsa-mir-96	chr7	1.29E+08	1.29E+08	-	0	0	1	0	10	0	61	0	24	0	miRNA
hsa-mir-9903	chr8	99405873	99405966	-	0	0	0	0	6	0	0	0	0	0	miRNA
hsa-mir-9985	chrY	4474161	4474269	+	0	18	18	0	4	0	2	0	0	1	miRNA
hsa-mir-99a	chr21	17911409	17911489	+	0	0	39	0	0	0	39	1	24	0	miRNA

Supplementary Table-S3: mi-RNAs associated with mitochondrial biogenesis:

Common Identified mi-RNA in our List vs mi-RNA Data Base
hsa-miR-16-5p
hsa-miR-22-3p
hsa-miR-340-5p
hsa-miR-15b-5p
hsa-miR-182-5p
hsa-miR-203a-3p
hsa-miR-433-3p
hsa-miR-1291-5p
hsa-miR-1291
hsa-miR-323a-3p
hsa-miR-200a-3p
hsa-miR-6876-3p
hsa-miR-1185-1-3p
hsa-miR-1185-2-3p
hsa-miR-132-3p
hsa-miR-16-1-3p
hsa-miR-181c-3p
hsa-miR-195-5p
hsa-miR-26b-3p
hsa-miR-3120-3p
hsa-miR-3146
hsa-miR-3158-3p
hsa-miR-34a-3p
hsa-miR-362-3p
hsa-miR-424-5p
hsa-miR-4712-3p
hsa-miR-4746-3p
hsa-miR-497-5p
hsa-miR-5001-3p
hsa-miR-503-5p
hsa-miR-6762-3p
hsa-miR-6813-5p
hsa-miR-6826-5p
hsa-miR-6838-5p
hsa-miR-885-5p
hsa-miR-3146
hsa-miR-5696

Supplementary Figure S1

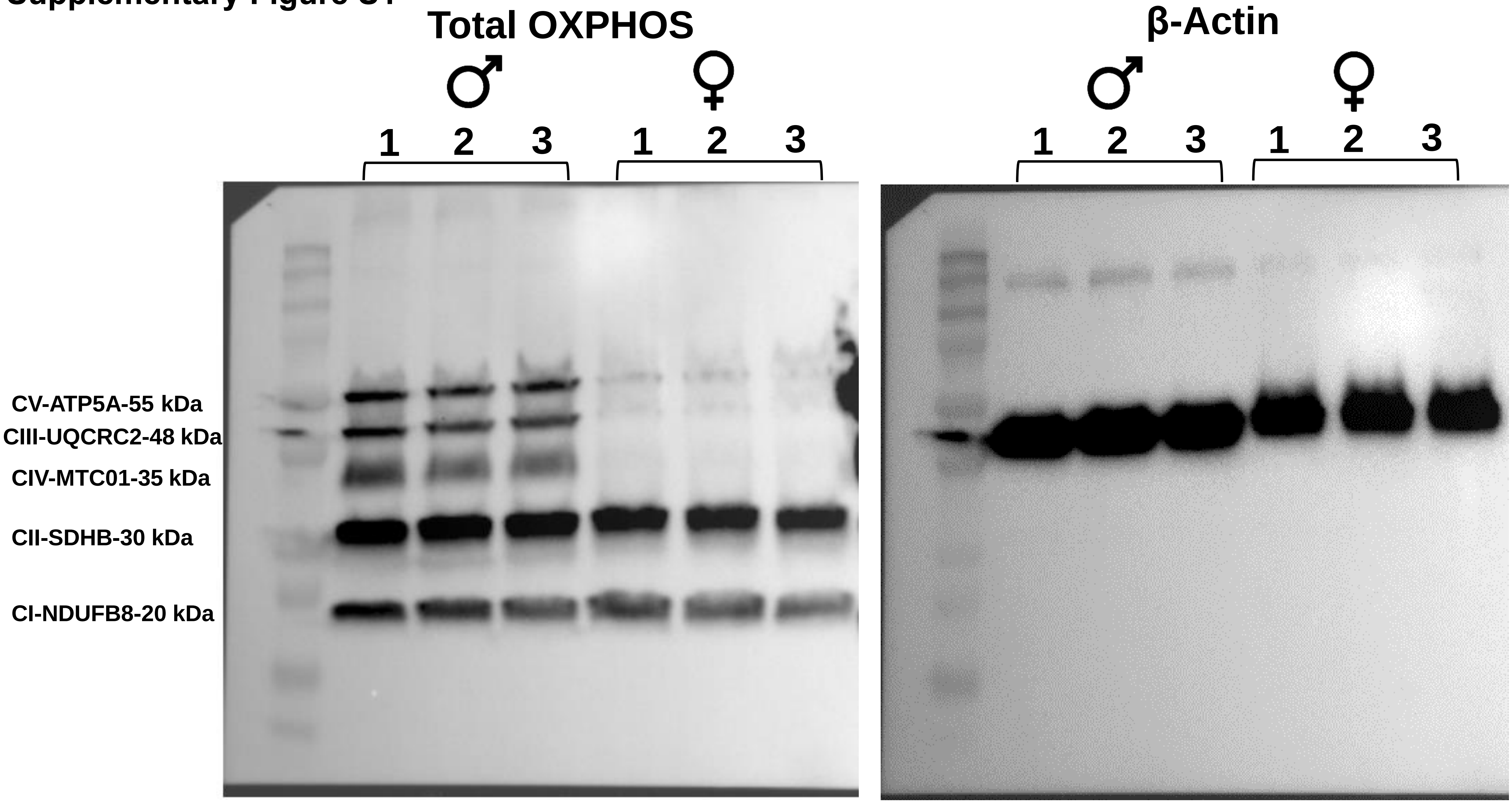

Supplement: Supplementary file 1 [file cells-13-00046-s001.zip › cells-2746512-supplementary.pdf]
